# Synthesis, docking study and biological evaluation of ᴅ-fructofuranosyl and ᴅ-tagatofuranosyl sulfones as potential inhibitors of the mycobacterial galactan synthesis targeting the galactofuranosyltransferase GlfT2

**DOI:** 10.3762/bjoc.16.152

**Published:** 2020-07-27

**Authors:** Marek Baráth, Jana Jakubčinová, Zuzana Konyariková, Stanislav Kozmon, Katarína Mikušová, Maroš Bella

**Affiliations:** 1 Institute of Chemistry Slovak Academy of Sciences, Dúbravská cesta 9, SK-845 38 Bratislava, Slovakia; 2 Department of Biochemistry, Faculty of Natural Sciences, Comenius University in Bratislava, Ilkovičova 6, SK-842 15 Bratislava, Slovakia

**Keywords:** GlfT2, molecular modeling, *mycobacterium tuberculosis*, synthesis, transition state inhibitors

## Abstract

A series of ten novel ᴅ-fructofuranosyl and ᴅ-tagatofuranosyl sulfones bearing a 1-*O*-phosphono moiety and three different substituents at C-2 has been prepared. Due to the structural similarities of these scaffolds to the native substrate of mycobacterial galactofuranosyltransferase GlfT2 in the transition state, we evaluated these compounds by computational methods, as well as in an enzyme assay for the possible inhibition of the mycobacterial galactan biosynthesis. Our data show that despite favorable docking scores to the active site of GlfT2, none of these compounds serve as efficient inhibitors of the enzymes involved in the mycobacterial galactan biosynthesis.

## Introduction

Tuberculosis (TB) is one of the most prevalent infectious diseases, with estimated 10 million new cases each year. It causes about 1.5 million deaths annually and is amongst the top 10 causes of death worldwide. The emergence of multidrug-resistant and extensively drug-resistant TB has become a major public health concern as the success rate of the treatment in these cases is only 56% and 39%, respectively [1]. Clearly, the need for new and more efficient TB drugs is pressing. Among the novel therapeutic agents developed for TB, there are several compounds that target the cell wall of *Mycobacterium tuberculosis*, the causative agent of the disease [2]. The mycobacterial cell wall core is formed by the mycolyl–arabinogalactan–peptidoglycan (mAGP) complex, which provides a highly hydrophobic and resistant barrier surrounding the bacterium [3]. The galactan component of the cell wall consists of polysaccharide chains composed of approximately 22 galactofuranose (Gal*f*) residues linked by alternating β-(1→5)- and β-(1→6)-glycosidic bonds [4]. The Gal*f* monomer is restricted to some bacteria, fungi and a few protozoan species, and it seems to be absent in humans [5]. The enzymes participating in the galactan build-up could thus be considered as potential targets for new antitubercular drug developments [6]. Mycobacterial galactan is synthesized by two bifunctional galactofuranosyltransferases, GlfT1 and GlfT2 (Supporting Information File 1, Figure S1). The former one initiates the galactan biosynthesis by addition of the first two Gal*f* residues to decaprenyl-P-P-GlcNAc-Rha, (glycolipid 2, GL2), which serves as a lipid carrier for arabinogalactan polymerization [7–8]. The latter enzyme, GlfT2, extends the product of GlfT1-catalyzed reaction, decaprenyl-P-P-GlcNAc-Rha-Gal*f*_2_ (glycolipid 4, GL4), producing the lipid-linked galactan polymer [8]. Both enzymes require an activated sugar donor uridine diphosphate (UDP)-Gal*f*, which is synthesized from UDP-galactopyranose (UDP-Gal*p*) by the enzyme UDP-Gal*p* mutase [9] (Supporting Information File 1, Figure S1).

The recently published GlfT2 X-ray structure with a UDP donor part [10] was used in the reaction mechanism studies using computational chemistry methods. The probable reaction mechanisms were studied by hybrid DFT QM/MM molecular dynamics simulations [11] where the possible transition state (TS) structures were localized. The observation of the possible TS structure opens the opportunities for the in silico based design of possible GlfT2 inhibitors that mimic the TS structure.

**Figure 1:**
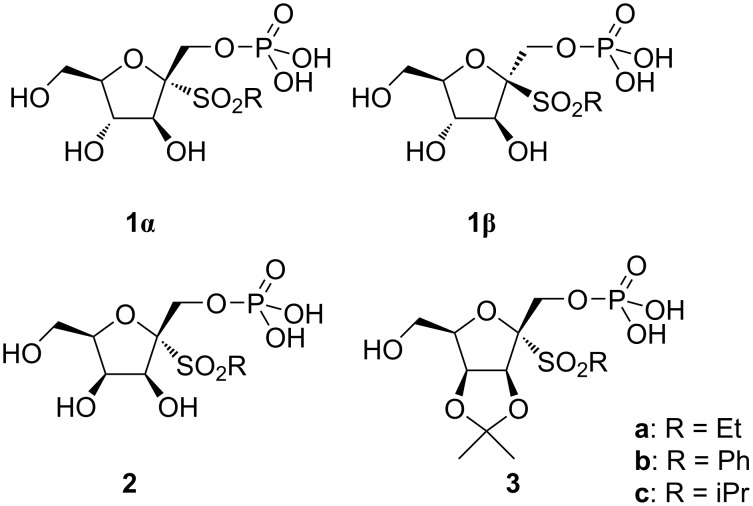
Target ᴅ-fructofuranosyl and ᴅ-tagatofuranosyl sulfones **1**‒**3**.

The galactofuranosyltransferase GlfT2 is a bisubstrate enzyme with a single catalytic domain and its catalytic reaction transition state shares some structural similarities with the previously modeled *N*-acetylglucosaminyltransferase I (GnT-I) reaction transition state [11]. Previously, we have designed 2-thiohexofuranoside skeletons bearing a phosphate group in position 1 by molecular modeling as potential transition state inhibitors of human glycosyltransferase I (GnT-I) [12], the bisubstrate enzyme requiring a metal co-factor for the proper synthesis of *N*-glycans [13]. Subsequently, pioneer structures based on ᴅ-fructofuranose [14], ᴅ-tagatofuranose or ᴅ-psicofuranose [15–16] have been prepared, but their inhibitory activity could not have been tested due to their low stability under standard conditions [16]. Further investigations on their more stable analogs resulted in the synthesis of stable 1-*O*-phoshono-β-ᴅ-psicofuranosyl sulfone, which was prepared by a simultaneous phosphorylation and oxidation of ethyl 2-thio-β-ᴅ-psicofuranoside with dibenzyl *N*,*N*-dimethylphosphoramidite catalyzed by 1*H*-tetrazole and followed by treatment with an excess of 3-chloroperbenzoic acid [16]. Fortunately, the molecular modeling studies did not bring any significant contention about the negative influence of the SO_2_ group in the target molecule on the standard molecular docking parameters [16]. Based on this fact, a new generation of stable possible inhibitors of GnT-I, comprising structures derived from the ᴅ-fructofuranose skeleton bearing an SO_2_ group and sulfated at the position C-1 were prepared [17]. However, these compounds did not show any significant inhibitory activity [17]. Therefore, we decided to prepare structures originally suggested by molecular modeling having the 1-*O*-phosphate group stabilized by anomeric sulfones on the ᴅ-fructofuranose and ᴅ-tagatofuranose skeleton (**1**–**3**, Figure 1). Similarities in the TS structures of GnT-I and GlfT2 prompted us to examine these molecules against GlfT2, a possible target for the development of the drugs against tuberculosis. Despite that the studied structures do not contain the galacto configuration on the furanose ring, the studied compounds mimic the TS structure of the GlfT2 catalytic reaction. The TS structure mimetics are stable compounds designed to mimic the unstable TS complex structure and also mimic its partial charges and charge distribution. Herein, the molecular docking, synthesis and inhibitory activity of ᴅ-fructofuranosyl and ᴅ-tagatofuranosyl sulfones **1**–**3** against GlfT2 are discussed. Moreover, we extended the scope of the simultaneous phosphorylation and oxidation to 2-thio-ᴅ-fructo- and 2-thio-ᴅ-tagatofuranosides as the key step in the synthesis of target structures.

## Results and Discussion

### Docking study

In the presented study, the compounds based on the fructofuranose and tagatofuranose derivatives (Figure 1) were docked into the GlfT2 structure. The proposed structures nicely follow the probable TS structure of the GlfT2 catalytic reaction as is shown in Supporting Information File 1, Figure S2. The available GlfT2 X-ray structure contains only the UDP part of the native UDP-Gal*f* donor substrate (PDB ID: 4FIY). The structure of the UDP-Gal*f* was docked into the GlfT2 structure to obtain the docking score of the native substrate. The observed UDP-Gal*f* docking score serves as a reference for the studied compounds. The obtained UDP-Gal*f* docking pose showed almost the same binding mode of the UDP moiety as UDP present in the X-ray structure (Supporting Information File 1, Figure S3). A small movement was observed only for the diphosphate part due to the proper accommodation of the Gal*f* moiety within the GlfT2 binding pocket. This suggested that the UDP-Gal*f* docked binding pose was reliable and could be used as a reference for the docking of the studied structures. All fructofuranose and tagatofuranose derivatives were docked into the GlfT2 structure obtained by QM/MM molecular dynamics simulations, which representes the structure close to the transition state structure of the GlfT2 catalytic reaction. The best ten docking poses of each docked molecule were saved for further analyses.

**Figure 2:**
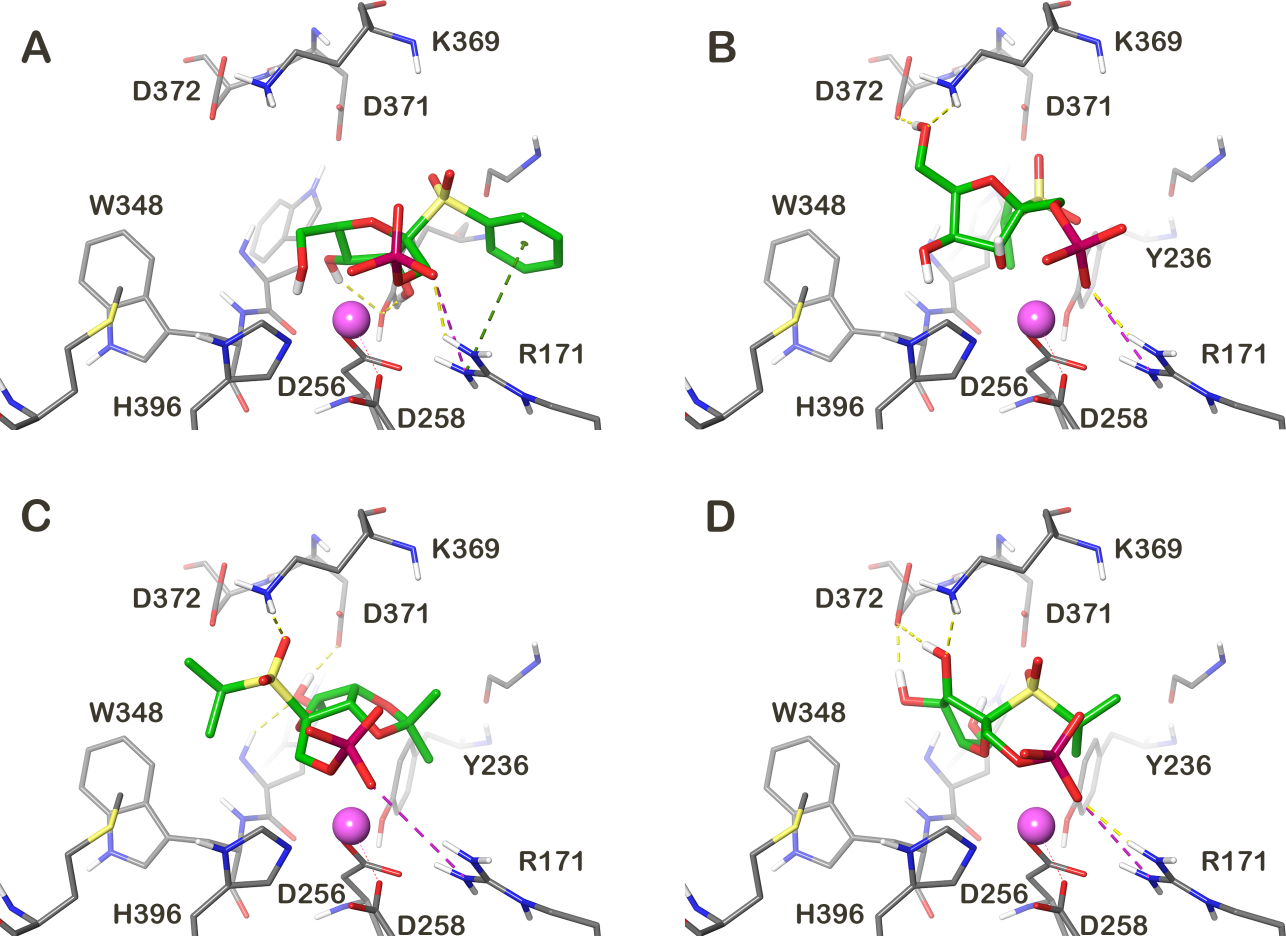
Molecular representation of the best binding poses of the four compounds with the predicted highest affinity. (A) **1bα**, (B) **2c**, (C) **3c** and (D) **1cβ**. The molecule–GlfT2 interactions are depicted as dashed lines (yellow – hydrogen bonds, magenta – salt bridge, green – cation/π interaction). The Mg^2+^ ion is shown as a purple sphere.

The docking predicted one or two main binding modes for each docked molecule. The most populated binding mode also contained the binding pose with the lowest docking score (for which the highest affinity was predicted). Only the binding modes with the lowest docking score will be discussed below. The observed binding modes of the docked molecules revealed some common interaction patterns. Interactions of the PO_4_ group with a metal ion and arginine R171 were observed in almost all docking poses and in some cases the C2 hydroxy group also interacted with a metal ion. The C5 hydroxy group very often interacts with the catalytic base aspartate D372 side chain. Moreover, in the poses where this interaction has been found, the C5 hydroxy group creates a hydrogen bond with the ammonium group of the lysine K369 residue. In many docking poses the interaction of the C3 hydroxy group with the aspartate D256 has also been seen. The SO_2_ unit attached to alkyl/aryl substituents have been found in two main binding modes. In one binding mode the alkyl/aryl part is bound towards the uridine binding pocket of the UDP-Gal*f*. The second binding mode was found in the place where the acyclic tail of the native Gal*f* is bound. The binding poses with the highest docking score of the four compounds with the highest predicted affinity are shown in Figure 2. The highest binding affinity was predicted for compounds **1bα**, **2c**, **3c** and **1cβ**. It can be seen that the compounds **2c**, **3c** and **1cβ** share the common binding interactions described above. However, the **1bα** binding mode slightly differed from the modes of the other compounds. In this case the PO_4_ and hydroxy group on C5 interacted with a metal ion and the hydroxy groups on C2 and C3 created the hydrogen bond with the tyrosine Y236 hydroxy group and the phenyl ring created a cation–π stacking interaction with the arginine R171 side chain (Figure 2).

The observed theoretical binding energy in kcal/mol represented by the docking scores and the predicted binding affinities for the UDP-Gal*f*, fructofuranose, and tagatofuranose compounds are listed in Table 1.

**Table 1:** Observed docking score and predicted *K*_i_ values of the docked compounds **1**‒**3**.

Compound	Docking score [kcal/mol]	Predicted *K*_i_ [µM]

UDP-Gal*f*	−9.192	0.333^a^
**1aα**	−7.505	5.148
**1bα**	−8.678	0.767
**1cα**	−7.952	2.492
**1cβ**	−8.648	0.805
**2a**	−7.701	3.745
**2b**	−8.388	1.228
**2c**	−8.389	1.226
**3a**	−6.382	31.839
**3b**	−6.900	13.739
**3c**	−8.549	0.946

^a^Experiment: 250 or 380 µM at 37 °C [10,18–19].

Firstly, the UDP-Gal*f* native donor substrate was docked into the GlfT2 crystal structure to observe the reference docking score and to predict the *K*_i_ value for the native donor substrate. The predicted *K*_i_ value of the UDP-Gal*f* is 0.333 µM, however, the experimentally observed *K*_m_ values of the UDP-Gal*f* binding were 250 or 380 µM at 37 °C [10,18–19]. All the predicted values were calculated for this temperature. This observation suggested that the calculated docking score overestimated the predicted binding affinities by three orders of magnitude. Unfortunately, the experimental *K*_m_ or *K*_i_ values of the structures similar to the studied compounds were not available and therefore we were not able to create a more precise linear regression model for the binding affinity prediction. For this reason, we could use only a coarse correction for the calculated *K*_i_ values and the experimental *K*_i_ values can be expected in the mM and sub-mM range rather than in the µM and sub-µM range. However, the obtained predicted *K*_i_ values were reliable for the comparison of the fructofuranose and tagatofuranose compounds binding affinities with the UDP-Gal*f* binding affinity. Moreover, the *K*_i_ values gave a more realistic image about the affinity differences between the compounds than only the predicted binding energies. The observed docking score of the studied compounds ranged from −6.4 to −8.7 kcal/mol. All these values were higher than the observed docking score for the UDP-Gal*f*. This means that the docking predicted a lower affinity for the studied compound than for the native substrate. Recalculation of the observed docking scores to the *K*_i_ values showed that the predicted affinity of the studied compounds ranged from 0.7 µM to 32 µM. In case of the best four compounds with the highest affinity (**1bα, 2c, 3c, 1cβ**) the *K*_i_ values ranged up to 1.2 µM. The predicted affinity of these compounds was just three to four times lower than the predicted affinity for the UDP-Gal*f*. Such observation is very promising, due to the fact that the studied molecules were smaller, they did not show as many interactions as the native donor substrate and created only one strong interaction with the metal ion cofactor. So further modification of these four molecules can lead to the compounds with similar or higher affinities to the GlfT2 than UDP-Gal*f*.

### Chemistry

Within the structure of target compounds **1–3**, the carbohydrate skeletons resembled the donor substrate: various RSO_2_ aglycons mimic an approaching acceptor substrate while the 1-*O*-phosphono moiety represents the leaving UDP [20].

**Scheme 1:**
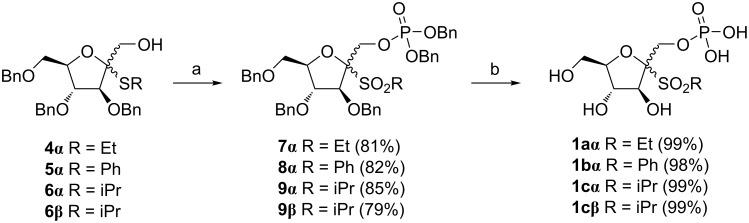
Reagents and conditions: a) 1. (BnO)_2_P-NMe_2_, 1*H*-tetrazole, 0 °C→rt, 1 h, 2. *m*-CPBA, 0 °C→rt, 1.5‒2 h; b) H_2_, 10% Pd/C, MeOH, rt, 3‒4 h.

The synthesis of target compounds **1**–**3** consisted of two different strategies. The first one was applied to a series of ᴅ-fructofuranose derivatives **1** and started from the four known alcohols **4α**, **5α**, **5β** and **6α** [17]. Initial treatment of these alcohols with commercially available dibenzyl *N*,*N*-dimethylphosphoramidite as phosphorylating agent catalyzed by 1*H*-tetrazole and followed by direct oxidation with an excess of 3-chloroperbenzoic acid (*m*-CPBA, 12 equiv) led to the simultaneous phosphorylation of the hydroxy group and to the oxidation of the sulfide to give 1-*O*-dibenzyloxyphosphoryl-ᴅ-fructofuranosyl sulfones **7α**, **8α**, **8β** and **9α** in excellent yields (Scheme 1). Final removal of benzyl protecting groups by catalytic hydrogenation provided target 1-*O*-phosphono-ᴅ-fructofuranosyl sulfones **1aα**, **1bα**, **1bβ** and **1cα** as white powders after freeze drying.

**Scheme 2:**
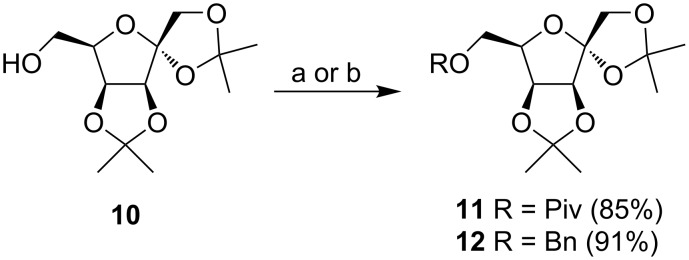
Reagents and conditions: a) PivCl, pyridine, CH_2_Cl_2_, rt, overnight; b) BnBr, NaOH, TBAB, THF, reflux, 3 h.

Based on the molecular docking results, the series of ᴅ-tagatofuranose compounds can be divided into two groups. The first group represented the more flexible structures **2** with free hydroxy groups in positions C3 and C4, while the second group of target compounds **3** has hydroxy groups on C3 and C4 protected as acetonides which made their structure more rigid. The synthesis started from the well-known diacetonide **10** [17]. Pivaloylation of the free hydroxy group at C-6 of **10** smoothly gave **11** in good yield. Likewise, 6-*O*-benzyl derivative **12** was prepared to reduce reaction steps and to ensure a clean progress of the benzyl protecting group removal by catalytic hydrogenation (Scheme 2).

**Scheme 3:**
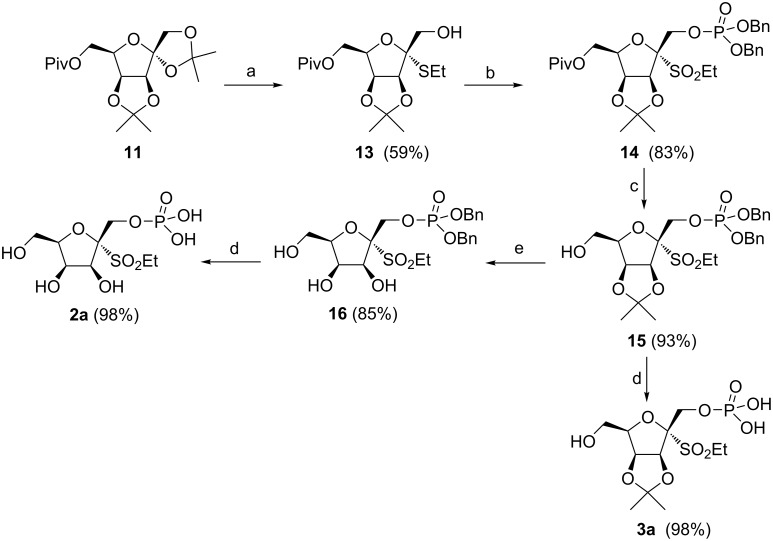
Reagents and conditions: a) EtSH, BF_3_∙OEt_2_, CH_2_Cl_2_, 0 °C, 2 h; b) 1. (BnO)_2_P-NMe_2_, 1*H*-tetrazole, 0 °C→rt, 1 h, 2. *m*-CPBA, 0 °C→rt, 1.5‒2 h; c) MeONa, MeOH, rt, 1.5 h; d) H_2_, 10% Pd/C, MeOH, rt, 3‒4 h; e) 3 M HCl, THF, 40 °C, 8 h.

The direct thioglycosylation of **11** with ethanethiol in the presence of BF_3_∙OEt_2_ afforded 2-thio-ᴅ-tagatofuranoside **13** in satisfactory yield (Scheme 3). In the course of the thioglycosylation, only the α-anomer of **13** was detected and isolated as the product. In general, the formation of α-anomers during the thioglycosylation of di-*O*-isopropylidene-ᴅ-tagatofuranoses **11** and **12** was controlled by the approach of a thiol from *exo*-face of the bicyclic skeleton. Subsequent simultaneous phosphorylation and oxidation of ethyl 2-thio-ᴅ-tagatofuranoside **13** under identical conditions as used for the fructofuranose pathway provided 1-*O*-dibenzyloxyphosphoryl-ᴅ-tagatofuranosyl sulfone **14** (Scheme 3). Removal of the pivaloyl protecting group in derivative **14** led to alcohol **15** as the common intermediate for the synthesis of target structures **2a** and **3a**. The fully deprotected target compound **2a** was obtained in good yield by acidic hydrolysis of the acetonide protecting group with 3 M HCl in THF followed by catalytic hydrogenation of **16**. On the other hand, direct catalytic hydrogenation of **15** furnished target structure **3a** with the hydroxy groups protected on C3 and C4 as an acetonide.

**Scheme 4:**
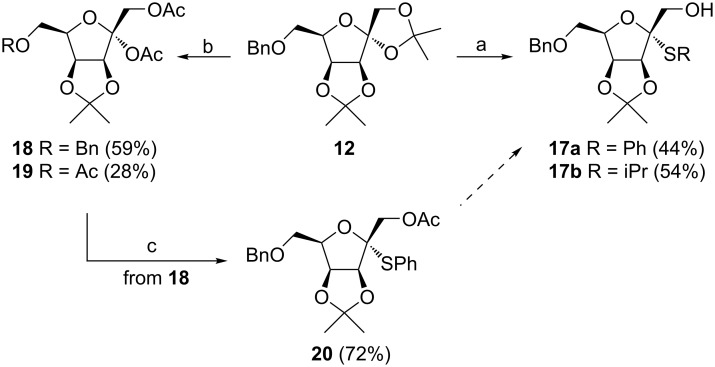
Reagents and conditions: a) PhSH, or iPrSH, BF_3_·OEt_2_, CH_2_Cl_2_, −5 °C, 1 h; b) BF_3_·OEt_2_, Ac_2_O, 0 °C, 1.5 h; c) PhSH, BF_3_·OEt_2_, CHCl_3_, −20 °C, 1.5 h.

Direct thioglycosylation of diacetonide **12** with corresponding thiols (PhSH, iPrSH) led to expected 2-thio-ᴅ-tagatofuranosides **17a** and **17b** in 44% and 54% yield, respectively. Moderate yields of 2-thio-ᴅ-tagatofuranosides **17** can be explained by cleavage of the benzyl protecting group [21] as well as further side reactions (Scheme 4).

**Scheme 5:**
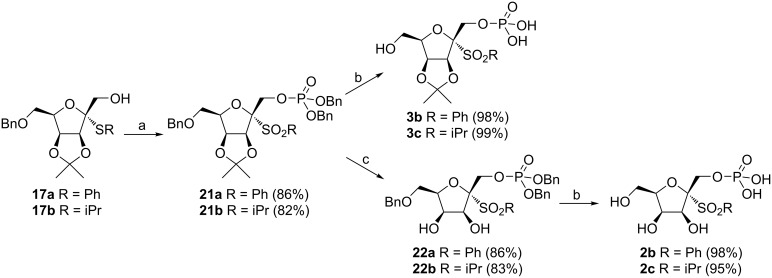
Reagents and conditions: a) 1. (BnO)_2_P-NMe_2_, 1*H*-tetrazole, 0 °C→rt, 1 h, 2. *m*-CPBA, 0 °C→rt, 1.5‒2 h; b) H_2_, 10% Pd/C, MeOH, rt, 3‒4 h; c) 3 M HCl, THF, 40 °C, 8 h.

In order to increase the yield of 2-thio-ᴅ-tagatofuranosides **17**, compound **12** was converted to the more reactive diacetate **18** under acetolysis conditions (Ac_2_O/BF_3_∙OEt_2_) [22]. However, besides the expected diacetate **18**, 1,2,6-tri-*O*-acetate **19** was observed and isolated as a byproduct (Scheme 4). The cleavage of the *O*-benzyl ethers followed by acetylation of the liberated hydroxy groups under acidic conditions was described previously, however, concentrated H_2_SO_4_ is usually employed as a promotor in acetic anhydride [23]. Although thioglycosylation of diacetate **18** with PhSH provided 2-thio-ᴅ-tagatofuranoside **20** in 72% yield, the overall synthesis of **17a** would contain two more reaction steps and, thus, be less effective. As the yields of 2-thio-ᴅ-tagatofuranosides **17** were comparable to the yield of 2-thio-ᴅ-tagatofuranoside **13** obtained by thioglycosylation of **11**, derivative **12** proved to be a more suitable substrate for the synthesis of target compounds **2** and **3** due to the possibility of simultaneous deprotection of the benzyl protecting groups in later stages of the synthesis. This advantage makes the overall synthesis of the target molecules one reaction step shorter in comparison with the synthesis starting from pivalate **11** (Scheme 3). With alcohols **17** in hand, the synthesis continued with their simultaneous phosphorylation and oxidation under the above-mentioned conditions to give 1-*O*-dibenzyloxyphosphoryl-ᴅ-tagatofuranosyl sulfones **21** (Scheme 5). Next, catalytic hydrogenation of **21** afforded the final acetonides **3b** and **3c** in excellent yields. The fully deprotected target structures **2b** and **2c** were obtained by acetonide hydrolysis under acidic conditions followed by the final catalytic hydrogenation of the benzyl protecting groups in derivatives **22**.

### Evaluation of the effects of the target compounds on the synthesis of lipid-linked galactan precursors

**Figure 3:**
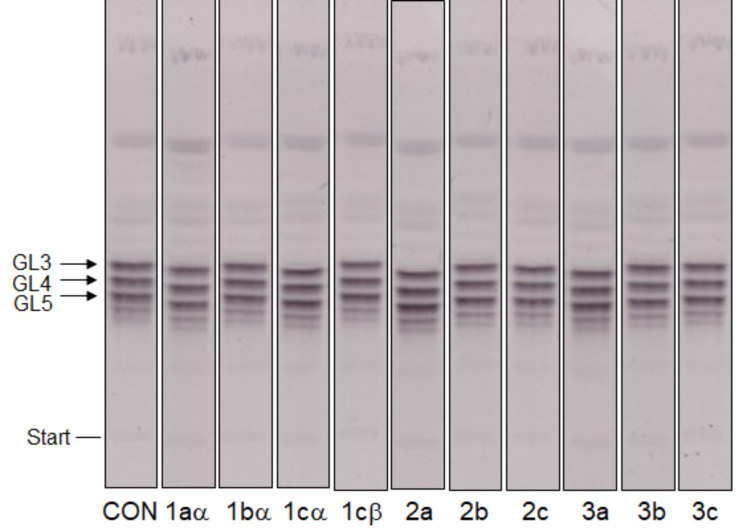
TLC analysis of the effects of the target compounds at 500 μM on the production of lower lipid-linked galactan precursors. CON, control; GL3–5, glycolipids 3–5. GL3 and GL4 are products of GlfT1, GL5 is produced by GlfT2.

The availability of a series of compounds with different predicted affinities towards GlfT2 according to the docking studies (Table 1) encouraged us to evaluate them experimentally. The compounds were tested in an assay using an enzymatically active fraction of cell envelope from *Mycobacterium smegmatis* mc^2^155 and UDP-[^14^C]Gal*p* as a tracer of the build-up of lipid-linked galactan precursors. The crude enzymes used in the assay allow for in situ synthesis of the acceptor for galactan polymerization, decaprenyl-P-P-GlcNAc-Rha (GL2), from endogenous decaprenyl phosphate and sugar nucleotides UDP-GlcNAc and TDP-Rha supplied in the reaction mixture. Conversion of UDP-[^14^C]Gal*p* to UDP-[^14^C]Gal*f*, which is required by mycobacterial galactofuranosyl transferases, was enabled by trace amounts of the cytosolic UDP-Gal*p* mutase present in the prepared enzyme fraction. Consequently, this assay monitored not only GlfT2, but also activities of other enzymes employed in the synthesis of its acceptor substrate, decaprenyl-P-P-GlcNAc-Rha-Gal*f*_2_ (GL4). Based on the previous experimental data, which established *K*_m_ values for GlfT2 250 or 380 µM at 37 °C [10,18–19], we tested the whole set of target compounds at a concentration of 500 µM. TLC analysis of the lower galactan precursors, decaprenyl-P-P-GlcNAc-Rha-Gal*f*_1-3_ (GL3-5) shows that under our experimental conditions none of the enzymes involved in the synthesis of lipid-linked galactan, including the galactofuranosyl transferases GlfT1 (produces GL3 and GL4) and GlfT2 (produces GL5), were affected (Figure 3).

**Figure 4:**
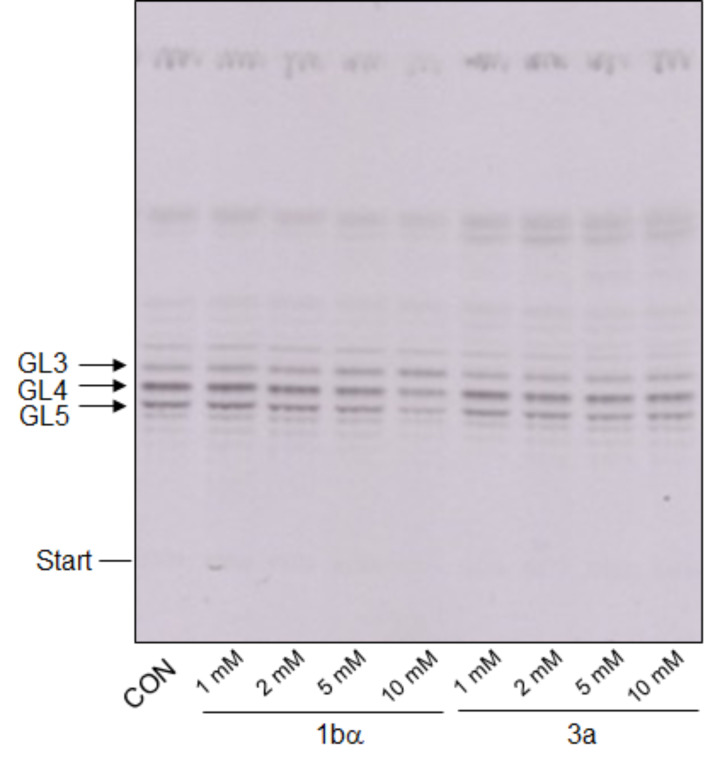
TLC analysis of the dose effects of the compounds **1bα** and **3a** on production of lower lipid-linked galactan precursors. CON, control; GL3–GL5, glycolipids 3–5. GL3 and GL4 are the products of GlfT1, GL5 is produced by GlfT2.

Despite the predicted low docking scores for the compounds **1bα**, **2c**, **3c** and **1cβ**, the quantification of the incorporation of the radioactive label to the more polymerized lipid-linked galactan precursors did not reveal inhibitory effects (Supporting Information File 1, Table S1). Nevertheless, our recent summary of the efforts towards identification of GlfT1 and GlfT2 inhibitors revealed that most compounds were reported to have effects on these enzymes at low mM concentrations (up to 8 mM) [6]. These molecules were designed as substrate mimics or transition state analogs and their IC_50_ values, ranging between 0.332–3.85 mM were established by a spectrophotometric assay with a purified recombinant GlfT2 enzyme, UDP-Gal*f* as a donor substrate and analogs of the acceptor substrate [24–30]. The best reported IC_50_ value (0.180 mM) for a putative GlfT2 inhibitor was obtained for a fluorinated exo-glycal analogue of UDP-Gal*f* in a radiometric assay with a glycolipid acceptor substrate and crude mycobacterial enzymes [31]. Therefore, we decided to examine compounds **1bα** and **3a,** representing the best and the worst inhibitors, respectively, according to the docking study, in the range of 1–10 mM. Interestingly, while compound **1bα** showed a dose-dependent inhibitory effect in this experiment, compound **3a** did not (Table 2). Although the crude system that we used in the enzymology experiments precludes precise kinetic characterization of the studied inhibitors on the target enzyme, GlfT2, it does have some advantages compared to the spectrophotometric GlfT2 assay [19]. Firstly, in the crude assay GlfT2 uses its natural acceptor, GL4, which could affect its activity and thus the outcome of the inhibition studies. Secondly, it allows to evaluate inhibition of the enzymes involved in GL4 production (WecA, WbbL, UGM, GlfT1; Supporting Information File 1, Figure S1) [6] by the tested compounds, which could indicate the unspecific effects. These cannot be precluded, especially if relatively high concentrations of the studied molecules are required for achieving substantial enzyme inhibition, which is the case for the reported GlfT2 inhibitors [6,24]. In our experiment TLC examination of the lower galactan precursors (GL3–5) points to subtle changes in their relative amounts in the presence of **1bα** in the reaction mixture (Figure 4). In the control reaction, the major radiolabeled glycolipid species are GL4, the product of GlfT1 and GL5, the product of GlfT2. In the presence of 10 mM **1bα** the predominant bands are GL3 and GL4, the products of GlfT1, which could point to a GlfT2 inhibition.

**Table 2:** Evaluation of the effects of compounds **1bα** and **3a** on the synthesis of the lipid-linked galactan polymer^a^.

Compound	Set1 (dpm)	Set2 (dpm)	Average	% of inhibition

control	11,449	9,587	10,518	0
1 mM **1bα**	10,041	11,384	10,712	−2
2 mM **1bα**	9,912	8,937	9,425	10
5 mM **1bα**	7,124	6,354	6,739	36
10 mM **1bα**	3,051	2,887	2,969	72
1 mM **3a**	8,602	8,638	8,620	18
2 mM **3a**	8,580	6,498	7,539	28
5 mM **3a**	6,054	6,029	6,041	43
10 mM **3a**	7,631	7,063	7,347	30

^a^The values in the table represent total incorporation of radioactive galactose from UDP-[^14^C]-Gal into the lipid-linked galactan polymer extracted by the solvents TT3 and E-soak (see Experimental part in Supporting Information File 1) in duplicate sets. The experiments were performed with 250 μg of the cell envelope protein in the reaction mixtures.

## Conclusion

In summary, a small library of ᴅ-fructofuranosyl and ᴅ-tagatofuranosyl sulfones **1**‒**3** was synthesized. We performed docking studies of these compounds into the active site of GlfT2 by computational chemistry methods. Although the docking study showed good binding affinities of the prepared compounds towards the GlfT2 active site, their biological evaluation revealed a very poor effect on the synthesis of lipid-linked galactan. However, the experiments evaluating the dose effects of the predicted best (**1bα**) and the worst (**3a**) inhibitor on the mycobacterial galactan synthesis indicated the possibility of GlfT2 inhibition by **1bα** at the highest tested concentration (10 mM). However, it is clear that given the high concentration of this compound required to achieve inhibitory effects on the galactan synthesis, this approach requires further optimization. Indeed, our pilot docking study was performed with compounds initially targeted at the transition state of GnT-I. Moreover, presented structures do not include the uridine mimicking part which can increase the binding affinity significantly. Attaching of this part is a topic of further synthesis and second-generation compound library preparation. We believe that our further efforts, both in synthetic and computational fields, will point to the potential of the in silico methods for the design of new GlfT2 inhibitors*.*

## Supporting Information

The Supporting Information provides the biochemical pathway for the biosynthesis of mycobacterial galactan, superimposition of docked structures and evaluation of the effects of the target compounds on the synthesis of the lipid-linked galactan polymer. Complete experimental procedures, spectral characterization of all prepared compounds and copies of their ^1^H NMR and ^13^C NMR spectra are also provided.

File 1 Experimental and analytical data.
