# Supplementary material for: Synthesis, docking study and biological evaluation of ᴅ-fructofuranosyl and ᴅ-tagatofuranosyl sulfones as potential inhibitors of the mycobacterial galactan synthesis targeting the galactofuranosyltransferase GlfT2
Source: Beilstein J Org Chem. 2020 Jul 27;16:1853–62. doi: 10.3762/bjoc.16.152 (PMC7404141; doi:10.3762/bjoc.16.152)
Supplement: File 1 — Experimental and analytical data. [file Beilstein_J_Org_Chem-16-1853-s001.pdf]

BEILSTEIN JOURNAL OF ORGANIC CHEMISTRY

Supporting Information

for

Synthesis, docking study and biological evaluation of D-fructofuranosyl and D-tagatofuranosyl sulfones as potential inhibitors of the mycobacterial galactan synthesis targeting the galactofuranosyltransferase GlfT2

Marek Baráth, Jana Jakubčinová, Zuzana Konyariková, Stanislav Kozmon,
Katarína Mikušová and Maroš Bella

Beilstein J. Org. Chem. **2020,** *16,* 1853–1862. doi:10.3762/bjoc.16.152

Experimental and analytical data

License and Terms: This is a supporting information file under the terms of the Creative Commons Attribution License (http://creativecommons.org/licenses/by/4.0). Please note that the reuse, redistribution and reproduction in particular requires that the authors and source are credited.
The license is subject to the *Beilstein Journal of Organic Chemistry* terms and conditions: (https://www.beilstein-journals.org/bjoc)

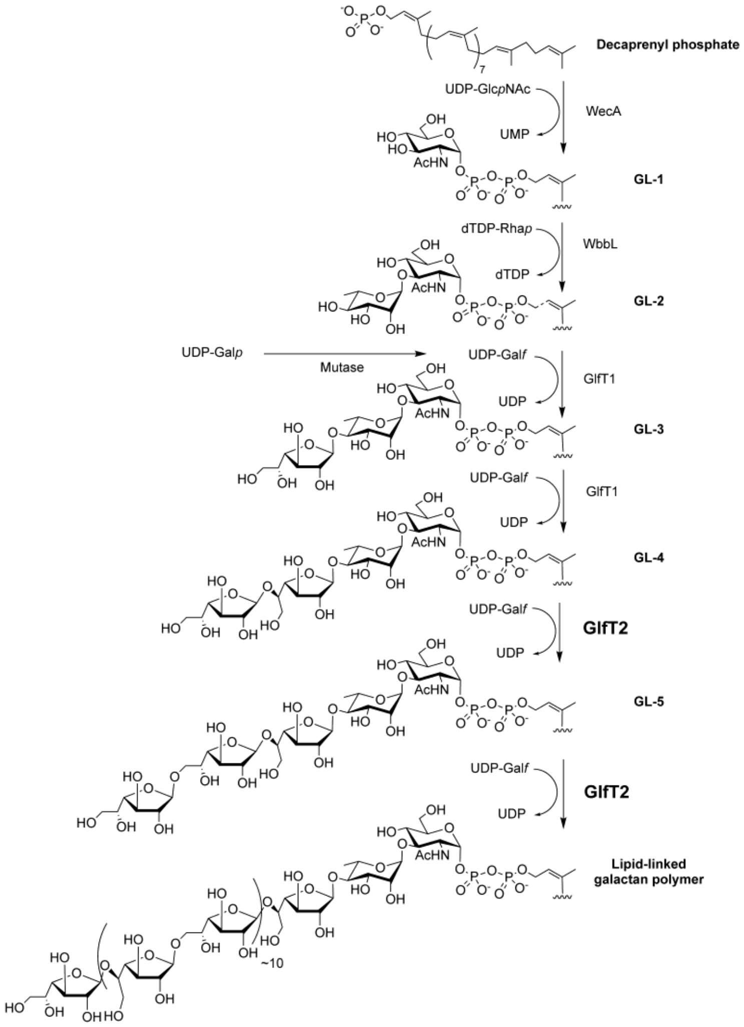

Figure S1: The biochemical pathway for biosynthesis of mycobacterial galactan. The synthesis is initiated by a transfer of GlcNAc-1-phosphate onto a decaprenyl-phosphate by the enzyme WecA and continues with the sequential addition of glycosyl residues to this lipid carrier by the enzymes WbbL, GlfT1 and GlfT2. GL-1 – GL-5 – glycolipid 1-5

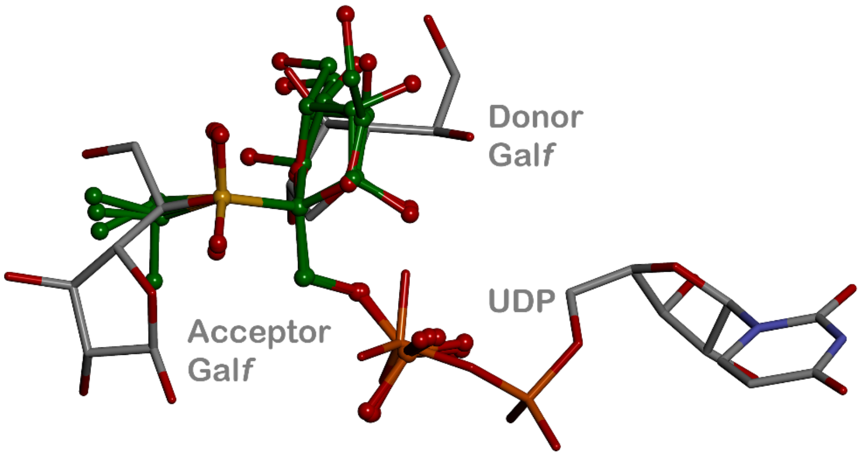

Figure S2: Superimposition of the studied D-fructofuranosyl and D-tagatofuranosyl scaffolds (green carbons) with the probable transition state structure (gray carbons) of the GlfT2 catalytic reaction.

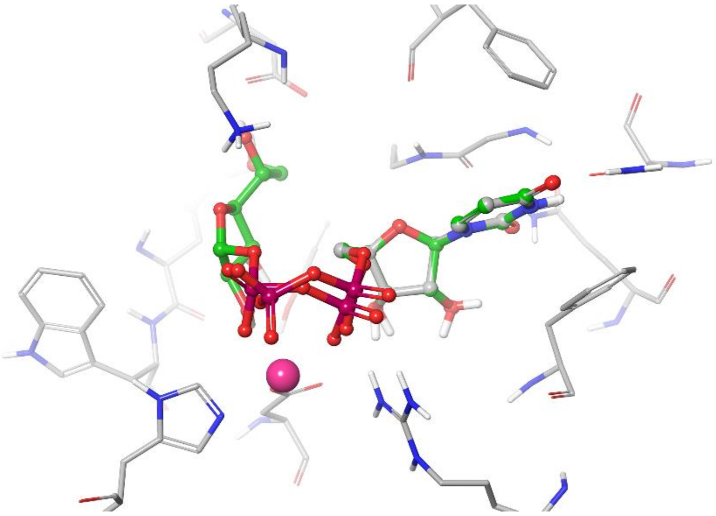

Figure S3: Superimposition of the docked UDP-Gal*f* (green carbons) with UDP in the crystallographic GlfT2 structure (light gray carbons).

Table S1. Evaluation of the effects of the target compounds on synthesis of the lipid-linked galactan polymer.*

	Experiment 1		Experiment 2	
	dpm	% incorporation	dpm	% incorporation
Control	28,650	100	21,058	100
**1aα**	28,285	99	23,612	112
**1bα**	**34,840**	**122**	**26,723**	**127**
**1cα**	27,370	96	18,752	89
**1cβ**	**30,505**	**106**	**24,498**	**116**
**2a**	35,125	123	16,950	80
**2b**	29,720	104	15,667	74
**2c**	**30,045**	**105**	**23,475**	**111**
**3a**	33,440	117	20,962	100
**3b**	28,685	100	19,267	91
**3c**	**27,670**	**97**	**19,693**	**94**

*The values in the table represent the total incorporation of radioactive galactose from UDP-[^{14}C]-Gal into lipid-linked galactan polymer extracted by the solvents TT3 and E-soak (see Experimental part) in two independent experiments. The experiments were performed with 500 µg of the cell envelope protein in the reaction mixtures. The compounds were added at a 500 µM concentration. The lines in bold correspond to the compounds with high binding affinities.

Experimental

Docking study

The structures of all docked compounds were optimized at the DFT B3LYP/6-31+G* level allowing full relaxation of the structures. After the geometric optimization the ESP charges were associated with all atoms at the same level. The ESP charges were calculated on the atom centers and values were fitted to reproduce the molecular quadruple moment. All calculations were done employing the Jaguar program as a part of the Schrodinger Suite 2018.04 [1] and were done in gas phase without the solvent effects. Such prepared molecules were docked into the receptor structure. Two structures of the GlfT2 were used as a receptor for the docking. The first one was a crystal structure of the GlfT2 in complex with UDP (PDB ID: 4FIY). The second structure used as a receptor was the structure representing the transition state of the catalytic reaction. This structure was obtained in the previous study by QM/MM molecular dynamics simulations [2]. Both structures were prepared using the Protein Preparation Wizard procedure implemented in the Schrodinger Suite 2018.04. The protein structures were protonated to pH 7 and the proton positions were optimized to create the optimal hydrogen bond network. The OPLS03 force field atom types and charges were associated to the protein structures. The center of the docking grid was placed close to the β-phosphate position or molecular center of the transferring galactofuranose molecule in case of the 4FIY or modeled structure, respectively. The docking box dimensions were set to 15 × 15 × 15 Å. The docking was done in the Glide program using the default setup for the standard precision flexible ligand docking. The best ten poses for each docked compound were kept for further analysis by the Glide Pose Viewer. The presented predicted K_i values of the

docked molecules were calculated based on the equation ΔG = RTln(K_i), where ΔG is the calculated predicted binding energy represented by the docking score, R is the gas constant and T is the temperature.

Chemistry

All reagents and anhydrous solvents were commercially sourced, and were used as received. The starting alcohols **4α**, **5α**, **5β**, **6α** and **10** were prepared according to a published method [3]. All solvents were of technical grade, and were distilled before use. Specific optical rotations were determined with a Jasco P-2000 polarimeter. ^{1}H and ^{13}C NMR spectra were recorded with a Bruker AVANCE III HD 400 spectrometer operating at 400 and 100 MHz working frequencies, respectively. Chemical shifts are given in (δ), and tetramethylsilane (TMS) was used as an internal standard for deuterated solvents used ($CDCl_3$/CD_3OD/D_2O). Coupling constants are given in Hz. All given ^{13}C spectra are proton decoupled. 2D NMR experiments (HSQC and COSY) were used for signal assignments. High-resolution mass spectra were recorded with an Orbitrap Elite (Thermo Scientific) mass spectrometer with ESI ionization in positive mode. Air humidity sensitive reactions were performed under inert atmosphere (nitrogen). Thin-layer chromatography (TLC) was carried out on glass plates pre-coated with TLC Silica gel 60 F_{254} (E. Merck). The plates were visualized by immersing into phosphomolybdic acid (PMA; 10% solution in ethanol) or H_2SO_4 (5% H_2SO_4 in ethanol) and heating at ca 200 °C with a heat gun. Column chromatography was carried out as flash chromatography on Silica gel 60 (E. Merck, 0.040–0.063 mm). Solvents used for flash chromatography were of technical grade,

and were distilled before use. If both anomers are mentioned the alpha anomer is always indicated by “α” and beta anomer by “β” in compound numbering.

*General procedure for preparation of 1-O-dibenzyloxyphosphoryl D-fructo- and D-tagatofuranosyl sulfones **7α**, **8α**, **9α**, **9β**, **14** and **21***

To a solution of alcohol (1 equiv) in anhydrous acetonitrile (ca. 200 mg of saccharide per 5 mL) cooled in an ice-water bath to 0 °C, a solution of 1*H*-tetrazole in acetonitrile (0.45 M, 4 equiv) was added dropwise followed by the addition of dibenzyl (*N*,*N*-dimethyl)phosphoramidite (3 equiv). After 5 min of stirring, the ice-water bath was removed and the mixture was slowly warmed up to room temperature (1 h). Next, the reaction was cooled in the ice-water bath to 0 °C and *m*-CPBA (12 equiv, 70%) was added in one portion. After 5 min of stirring, the ice-water bath was removed and the mixture was slowly warmed up to room temperature (1.5–2 h). The reaction mixture was diluted with CH_2Cl_2 (20 mL), washed with 5% aqueous solution of $Na_2S_2O_3$ (2 × 15 mL), saturated aqueous solution of $NaHCO_3$ (2 × 15 mL) and with brine (2 × 15 mL). Organic layer was dried over Na_2SO_4, filtered and the solvent was evaporated under reduced pressure. The crude products were isolated and purified by column chromatography on silica gel using AcOEt/hexanes (1:3, v/v) for fructofuranosyl sulfones **7α**, **8α**, **9α**, **9β** and (1:4, v/v) for tagatofuranosyl sulfones **16** and **23**.

*Ethyl 3,4,6-tri-O-benzyl-1-O-dibenzyloxyphosphoryl-α-D-fructofuranosyl sulfone (**7α**)*
Yield 120 mg, 81 %, colorless oil, $[\alpha]_D$ = +58.6 (c = 1.0, $CHCl_3$), ^{1}H NMR ($CDCl_3$): δ 1H NMR (400 MHz, $CDCl_3$) δ 7.35 – 7.13 (m, 25H, Har), 5.06 – 4.87 (m, 5H, 2×CH_2Ph, H-3), 4.68 (d, *J* = 11.5 Hz, 1H, CH_2Ph), 4.53 – 4.24 (m, 7H, 3×CH_2Ph, H-1,

H1a), 4.20 (ddd, *J* = 9.2, 5.0, 2.3 Hz, 1H, H-5), 4.07 – 3.97 (m, 1H, H-4), 3.54 (dd, *J* = 11.4, 2.4 Hz, 1H, H-6), 3.45 (dd, *J* = 11.4, 5.1 Hz, 1H, H-6a), 3.21 (dq, J = 13.7, 7.6 Hz, 1H, CH_2CH_3), 2.87 (dq, *J* = 13.7, 7.4 Hz, 1H, CH_2CH_3), 1.28 (t, *J* = 7.5 Hz, 3H, CH_2CH_3). ^{13}C NMR ($CDCl_3$): δ 137.8, 137.5, 136.8, 135.7, 135.6, 128.6, 128.5, 128.4, 128.2, 128.1, 128.0, 127.9, 127.8, 128.7 and 128.5 (C_{Ar}), 97.6 (C-2), 83.4 (C-3), 82.0 (C-4), 80.4 (C-5), 73.8, 73.3, 72.7, 69.5 and 69.4 (5×CH_2Ph), 68.7 (C-6), 66.5 (C-1), 44.7 (CH_2CH_3), 5.3 (CH_2CH_3). HRMS: m/z calcd for $C_{43}H_{47}O_{10}PS$: 809.2520 [*M*+Na]$^+$; found: 809.2518.

*Phenyl 3,4,6-tri-O-benzyl-1-O-dibenzyloxyphosphoryl-α-D-fructofuranosyl sulfone **(8α)***

Yield 158 mg, 82 %, colorless oil, $[\alpha]_D$ = +33.9 (c = 1.0, $CHCl_3$), ^{1}H NMR ($CDCl_3$): δ 7.98-7.95 (m, 2H, H_{Ar}), 7.62 (t, 1H, H_{Ar}), 7.31 (m, 27H, H_{Ar}), 5.16 (d, 1H, *J* = 7.2 Hz, H-3), 5.00 (d, 1H, *J* = 11.4 Hz, CH_2Ph), 4.90–4.67 (m, 6H, CH_2Ph), 4.65–4.53 (m, 2H, CH_2Ph, H-1), 4.51–4.34 (m, 4H, CH_2Ph, H-5), 4.19–4.07 (m, 2H, H-4, H-1a), 3.66 (dd, *J* = 11.4, 2.4 Hz, 1H, H-6), 3.55 (dd, *J* = 11.4, 5.7 Hz, 1H, H-6a). ^{13}C NMR ($CDCl_3$) δ 137.9, 137.6, 137.1, 135.5, 134.2, 130.4, 128.8, 128.5, 128.5, 128.4, 128.39, 128.34, 128.1, 127.8, 127.77, 127.71, 127.6, 127.5 and 127.4 (C_{Ar}), 98.5 (C-2), 83.8 (C-3), 82.3 (C-4), 80.0 (C-5), 74.1, 73.0, 72.8, 69.2 and 69.1, (5×CH_2Ph), 69.0 (C-6), 65.2 (C-1). HRMS: m/z calcd for $C_{47}H_{47}O_{10}PS$: 857.2520 [*M*+Na]$^+$; found: 857.2531.

Isopropyl 3,4,6-tri-O-benzyl-1-O-dibenzyloxyphosphoryl-α-D-fructofuranosyl sulfone ***(9α)***

Yield 110 mg, 85 %, colorless oil, $[\alpha]_D$ = +47.2 (c = 1.0, $CHCl_3$), ^{1}H NMR ($CDCl_3$): δ 7.39-7.24 (m, 23H, H_{Ar}), 7.19-7.17 (m, 2H, H_{Ar}), 5.07-5.01 (m, 5H, 2×CH_2Ph, H-3), 4.82 (AB_q, 1H, CH_2Ph), 4.61 (AB_q, 1H, CH_2Ph), 4.52-4.44 (m, 6H, H-1, H-1a, 2×CH_2Ph), 4.37-4.27 (m, 1H, H-5), 4.09 (dd, *J* = 9.4, 7.0 Hz, 1H, H-4), 3.65 (dd, *J* = 11.5, 2.4, 1H, H-6), 3.66-3.52 [m, 2H, H-6a, C*H*(CH_3)$_2$], 1.45 and 1.39 [2d, each 3H, *J* = 7.0 Hz, CH(CH_3)$_2$]; ^{13}C NMR ($CDCl_3$): δ 137.8, 137.5, 136.9, 135.6, 135.6, 135.5 and 135.5 (Car), 99.6 (C-2), 84.2 (C-3), 81.8 (C-4), 80.0 (C-5), 73.7, 73.1, 72.6, 69.5 and 69.4 (5×*C*H_2Ph), 68.9 (C-6), 66.4 (C-1), 53.9 [*C*H(CH_3)$_2$], 17.3 and 15.3 [CH(*C*H_3)$_2$]. HRMS: m/z calcd for $C_{44}H_{49}O_{10}PS$: 823.2676 $[M+Na]^+$; found: 823.2681.

Isopropyl 3,4,6-tri-O-benzyl-1-O-dibenzyloxyphosphoryl-β-D-fructofuranosyl sulfone ***(9β)***

Yield 165 mg, 79 %, colorless oil, $[\alpha]_D$ = -65.5 (c = 1, $CHCl_3$), ^{1}H NMR ($CDCl_3$) δ 7.39–7.26 (m, 23H, H_{Ar}), 7.21 (dd, *J* = 7.3, 2.3 Hz, 2H, H_{Ar}), 5.08 (dd, *J* = 8.3, 2.4 Hz, 4H, 2×CH_2Ph), 4.83 (d, *J* = 11.4 Hz, 1H, CH_2Ph), 4.68–4.43 (m, 9H, H-3, H-4, H-1, H-1a, 2xCH_2Ph), 4.21 (td, *J* = 7.0, 3.7 Hz, 1H, H-5), 3.70 (dd, *J* = 10.5, 6.8 Hz, 1H, H-6), 3.62–3.57 (m, 1H, H-6a), 3.51 [p, *J* = 6.9 Hz, 1H, C*H*(CH_3)$_2$], 1.24 [s, 3H, CH(CH_3)$_2$], 1.18 [d, *J* = 9.6 Hz, 3H, CH(CH_3)$_2$]; ^{13}C NMR ($CDCl_3$) δ 137.7, 137.6, 137.1, 135.5, 135.5, 128.7, 128.67, 128.64, 128.57, 128.53, 128.06, 128.00, 127.87 and 127.84 (14C_{Ar}), 99.9 (C-2), 85.0 (C-3), 82.48 (C-5), 82.41 (C-4), 74.1, 73.3, 72.9, 69.8 and 69.6 (5×*C*H_2Ph), 70.3 (C-6), 66.4 (C-1), 53.4 [*C*H(CH_3)$_2$], 16.6 and 16.5 [CH(*C*H_3)$_2$]. HRMS: m/z calcd for $C_{44}H_{49}O_{10}PS$: 823.2676 $[M+Na]^+$; found: 823.2678.

*Ethyl 1-O-dibenzyloxyphosphoryl-3,4-O-isopropylidene-6-O-pivaloyl-α-D-tagatofuranosyl sulfone (**14**)*

Yield 300 mg, 83 %, colorless oil, $[\alpha]_D$ = +72.1 (c = 1.0, $CHCl_3$), ^{1}H NMR ($CDCl_3$): δ 7.43–7.31 (m, 10H, H_{Ar}), 5.29 (d, *J* = 6.1 Hz, 1H, H-3), 5.17–5.07 (m, 4H, 2×CH_2Ph), 4.94 (dd, *J* = 6.1, 4.1 Hz, 1H, H-4), 4.69 (dt, *J* = 7.4, 4.1 Hz, 1H, H-5), 4.56 (dd, *J* = 12.0, 4.4 Hz, 1H, H-1), 4.43 (dd, *J* = 12.0, 4.2 Hz, 1H, H-6), 4.35 (dd, *J* = 12.1, 5.5 Hz, 1H, H-1a), 4.18 (dd, *J* = 12.0, 7.5 Hz, 1H, H-6a), 3.35 (dq, *J* = 14.0, 7.6 Hz, 1H, CH_2CH$_3$), 3.06 (dq, *J* = 14.0, 7.5 Hz, 1H, CH_2CH$_3$), 1.43 and 1.32 [2s, each 3H, C(CH_3)$_2$], 1.37 (t, *J* = 7.5 Hz, 3H, CH$_2$CH_3), 1.20 [s, 9H, C(CH_3)$_3$]; ^{13}C NMR ($CDCl_3$): δ 177.9 (C=O), 135.5, 135.4, 128.7, 128.5, 127.98 and 127.94 (C_{Ar}), 114.0 [*C*(CH$_3$)$_2$], 101.5 (C-2), 82.1 (C-5), 81.5 (C-3), 80.9 (C-4), 69.57 and 69.52 (2×*C*H$_2$Ph), 67.0 (C-1), 62.3 (C-6), 46.1 (*C*H$_2$CH$_3$), 38.7 [*C*(CH$_3$)$_3$], 27.1 [C(*C*H$_3$)$_3$], 25.6 and 24.0 [C(*C*H$_3$)$_2$], 5.4 (CH$_2$*C*H$_3$). HRMS: m/z calcd for $C_{30}H_{41}O_{11}PS$: 679.1739 $[M+K]^+$; found: 679.1725.

*Phenyl 6-O-benzyl-1-O-dibenzyloxyphosphoryl-3,4-O-isopropylidene-α-D-tagatofuranosyl sulfone (**21a**)*

Yield 335 mg, 86 %, colorless oil, $[\alpha]_D$ = +24.7 (c = 1.0, $CHCl_3$), ^{1}H NMR ($CDCl_3$): δ 8.00–7.83 (m, 2H, H_{Ar}), 7.57–7.45 (m, 1H, H_{Ar}), 7.43–7.21 (m, 15H, H_{Ar}), 7.23–7.09 (m, 2H, H_{Ar}), 5.53 (d, *J* = 6.0 Hz, 1H, H-3), 5.08 (dd, *J* = 6.0, 4.2 Hz, 1H, H-4), 5.03 (dt, *J* = 7.2, 4.0 Hz, 1H, H-5), 4.90 (t, *J* = 7.8 Hz, 2H, CH_2Ph), 4.86– 4.78 (m, 1H, CH_2Ph), 4.62 (dd, *J* = 11.9, 7.1 Hz, 1H, CH_2Ph), 4.56–4.45 (m, 3H, CH_2Ph, H-1), 4.33 (dd, *J* = 12.0, 6.7 Hz, 1H, H-1a), 3.85 (dd, *J* = 10.9, 4.0 Hz, 1H, H-6), 3.64 (dd, *J* = 10.9, 7.1 Hz, 1H, H-6a), 1.47 and 1.36 [2s, each 3H, C(CH_3)$_2$]; ^{13}C NMR ($CDCl_3$): δ 138.0, 136.0, 135.8, 135.78, 135.73, 134.0, 130.3, 128.58, 128.55, 128.50, 128.4,

128.36, 128.30, 127.7, 127.64, 127.61 and 127.4 (C_{Ar}), 113.9 [*C*(CH_3)$_2$], 102.1 (C-2), 83.6 (C-5), 81.7 (C-3), 81.4 (C-4), 73.2, 69.1 and 69.0 (2×*C*H_2Ph), 68.4 (C-6), 66.0 (C-1), 25.6 and 24.2 [2s, each 3H, C(C*H*$_3$)$_2$]. HRMS: m/z calcd for $C_{36}H_{39}O_8PS$: 685.1995 [*M*+Na]$^+$; found: 685.1997.

*Isopropyl 6-O-benzyl-1-O-dibenzyloxyphosphoryl-3,4-O-isopropylidene-α-D-tagatofuranosyl sulfone (**21b**)*

Yield 330 mg, 82 %, colorless oil, $[\alpha]_D$ = +12.4 (c = 1.0, $CHCl_3$), ^{1}H NMR ($CDCl_3$): 1H NMR (400 MHz, $CDCl_3$) δ 7.38 – 7.26 (m, 15H, Har), 5.33 (d, *J* = 6.1 Hz, 1H, H-3), 5.17 – 5.07 (m, 4H, 2×*C*H_2Ph), 4.94 (dd, *J* = 6.1, 4.1 Hz, 1H, H-4), 4.79 (dt, *J* = 7.2, 4.1 Hz, 1H, H-5), 4.64 – 4.39 (m, 4H, *C*H_2Ph, H-1, H-1a), 3.79 (dd, J = 10.8, 4.2 Hz, 1H, H-6), 3.73 – 3.56 [m, 2H, H-6a, C*H*(CH_3)$_2$], 1.53 – 1.41 (m, 6H, 2×CH_3), 1.38 – 1.29 (m, 6H, 2×CH_3). 13C NMR (101 MHz, $CDCl_3$) δ 138.01, 135.56, 128.62, 128.57, 128.37, 127.96, 127.94, 127.85, 127.64, and 127.42 (Car) and 113.72 [*C*(CH_3)$_2$], 103.2 (C-2), 83.1 (C-5), 82.2 (C-3), 81.0 (C-4), 73.2 (CH_2Ph), 69.5, 69.4 (2×CH_2Ph), 68.3 (C-6), 67.3 (C-1), 55.2 [*C*H(CH_3)$_2$], 25.7 (CH_3), 24.1 (CH_3), 17.2 (CH_3), 15.0 (CH_3). HRMS: m/z calcd for $C_{33}H_{41}O_{10}PS$: 699.1790 [*M*+K]$^+$; found: 699.1795.

*General procedure for preparation of target compounds **1aα, 1bα, 1cα** and **1cβ***

A mixture of perbenzyl monosaccharide (1 equiv) and Pd/C (10%, 50 mg) in MeOH (5-8 mL) was stirred at room temperature under a hydrogen atmosphere for 3–4 h [TLC, $CHCl_3$/MeOH, (3:1, v/v)]. When the reaction was complete, the mixture was filtered through a pad of Celite® and a filter cake was washed with a mixture of MeOH/distilled water [2:1 (v/v), 7 mL]. The filtrate was evaporated under reduced pressure and the residue was dissolved in distilled water (5 mL). The resulting

mixture was filtered through a PTFE syringe filter (0.45 μm) and the filtrate was lyophilized to afford compounds **1aα**, **1bα**, **1cα** and **1cβ**.

*Ethyl 1-O-phosphono-α-D-fructofuranosyl sulfone (**1aα**)*

Yield 41 mg, 99 %, white powder, $[\alpha]_D$ = +6.0 (c = 1.0, H_2O), ^1H NMR **(**D_2O): δ 4.79 (s, 1H, H-3), 4.18 (m, 2H, H-1, H-1a), 4.10 (s, 1H, H-4), 3.99-3.95 (m, 1H, H-5), 3.88-3.82 (m, 1H, H-6), 3.68-3.60 (m, 1H, H-6a), 3.48-3.41 (m, 1H, CH_2CH_3), 3.29-3.20 (m, 1H, CH_2CH_3), 1.32 (s, 3H, CH_2CH_3); ^{13}C NMR (D_2O): δ 97.8 (C-2), 82.0 (C-5), 75.9 (C-3), 74.1 (C-4), 63.7 (C-1), 60.0 (C-6), 44.9 (CH_2CH_3), 4.3 (CH_2CH_3). HRMS: m/z calcd for $C_8H_{17}O_{10}PS$: 359.0172 [*M*+Na]$^+$; found: 359.0177.

*Phenyl 1-O-phosphono-α-D-fructofuranosyl sulfone (**1bα**)*

Yield 11 mg, 98 %, white powder, $[\alpha]_D$ = +3.5 (c = 0.5, H_2O), ^1H NMR (D_2O): δ 7.81 (m, 5H, H_{Ar}), 4.49 (d, *J* = 7.9 Hz, 1H, H-3), 4.19–4.09 (m, 1H, H-1), 4.04 (t, *J* = 8.8 Hz, 1H, H-5), 3.78 (dd, *J* = 11.8, 5.4 Hz, 1H, H-1a), 3.66 (d, *J* = 11.5 Hz, 1H, H-6), 3.51 (dd, *J* = 13.6, 5.7 Hz, 2H, H-4, H-6a); 13C NMR (D_2O) δ 135.6, 130.1 and 129.7 (C_{Ar}), 100.8 (C-2), 81.5 (C-4), 77.2 (C-3), 73.7 (C-5), 62.5 (C-1), 60.1 (C-6). HRMS: m/z calcd for $C_{12}H_{17}O_{10}PS$: 407.0172 [*M*+Na]$^+$; found: 407.0180.

*Isopropyl 1-O-phosphono-α-D-fructofuranosyl sulfone (**1cα**)*

Yield 15 mg, 99 %, white powder, $[\alpha]_D$ = +8.0 (c = 1.0, $CHCl_3$), ^1H NMR **(**D_2O): δ 4.71 (d, *J* = 7.4 Hz, 1H, H-3), 4.18 (dd, *J* = 12.2, 5.6 Hz, 1H, H-1), 4.09 (dd, *J* = 12.0, 4.9 Hz, 1H, H-1a), 3.98 (d, *J* = 8.2 Hz, 1H, H-5), 3.91 (d, *J* = 4.5 Hz, 1H, H-4), 3.74 (d, *J* = 12.8 Hz, 1H, H-6), 3.64 [p, *J* = 9.6, 6.6 Hz, 1H, $CH(CH_3)_2$], 3.54 (dd, *J* = 13.0, 4.6 Hz, 1H, H-6a), 1.29 and 1.23 [2s, each 3H, $CH(CH_3)_2$]; ^{13}C NMR (D_2O): δ 99.9 (C-2),

81.9 (C-4), 76.8 (C-3), 73.7 (C-5), 63.7 (C-1), 60.1 (C-6), 54.4 [*C*H(CH_3)$_2$], 16.2 and 14.1 [CH(*C*H_3)$_2$]. HRMS: m/z calcd for $C_9H_{19}O_{10}PS$: 373.0329 [*M*+Na]$^+$; found: 373.0335.

*Isopropyl 1-O-phosphono-β-D-fructofuranosyl sulfone (**1cβ**)*

Yield 35 mg, 99 %, white powder, $[\alpha]_D$ = -12.1 (c = 1.0, $CHCl_3$), ^{1}H NMR (D_2O): δ 3.90 (m, 2H, H-5, H-4), 3.84 (m, 3H, H-3, H-6, H-6a), 3.73 (m, 2H, H-1, H-1a), 2.88 [p, *J* = 9.6, 6.6 Hz, 1H, C*H*(CH_3)$_2$], 1.18 and 1.12 [2s, each 3H, CH(C*H*$_3$)$_2$]; ^{13}C NMR (D_2O): δ 97.5 (C-2), 83.7 (C-4), 80.6 (C-3), 71.7 (C-5), 62.1 (C-1), 60.3 (C-6), 51.1 [*C*H(CH_3)$_2$], 16.7 [CH(*C*H_3)$_2$]. HRMS: m/z calcd for $C_9H_{19}O_{10}PS$: 389.0068 [*M*+K]$^+$; found: 389.0074.

*1,2:3,4-Di-O-isopropylidene-6-O-pivaloyl-α-D-tagatofuranose (**11**)*

Pivaloyl chloride (3.8 mL, 3.74 g, 31.0 mmol) was added dropwise to a stirred solution of diisopropylidene-D-tagatofuranose **10** [4] (6.70 g, 25.7 mmol) and pyridine (2.7 mL, 2.64 g, 33.4 mmol) in CH_2Cl_2 (100 mL) while being cooled in an ice-water bath. Next, the ice-water was removed and the reaction mixture was stirred at room temperature overnight. Then, the reaction mixture was washed with water (2×100 mL). The organic layer was dried with Na_2SO_4, and filtered, and the solvent was evaporated under reduced pressure. The crude product was purified by column chromatography on silica gel using EtOAc/hexanes (1:4, v/v) as the eluent to afford 6-*O*-pivaloyl-D-tagatofuranose **11** (7.53 g, 85%) as a thick colorless oil which crystallized in a refrigerator. M.p. 34-35 °C, $[\alpha]_D$ = +66.9 (*c* 1.16, $CHCl_3$), ^{1}H NMR ($CDCl_3$): δ 4.79 (dd, *J* = 5.8, 3.6 Hz, 1H, H-4), 4.61 (d, *J* = 5.8 Hz, 1H, H-3), 4.45 (dd, *J* = 11.6, 4.1 Hz, 1H, H-6), 4.26 (d, *J* = 9.7 Hz, 1H, H-1), 4.20 (dd, *J* = 11.6, 7.6 Hz,

1H, H-6‘), 4.12 (dt, J = 7.6, 3.8 Hz, 1H, H-5), 4.07 (d, J = 9.7 Hz, 1H, H-1‘), 1.46, 1.42, 1.40 and 1.30 [4s, each 3H, $C(CH_3)_2$], 1.21 [s, 9H, $C(CH_3)_3$]; ^{13}C NMR ($CDCl_3$): δ 178.2 (C=O), 112.9 and 111.6 [2×$C(CH_3)_2$], 111.8 (C-2), 85.1 (C-3), 79.9 (C-4), 77.1 (C-5), 69.3 (C-1), 62.2 (C-6), 38.7 [$C(CH_3)_3$], 27.1 [$C(CH_3)_3$], 26.5, 26.2, 26.0 and 24.9 [$C(CH_3)_2$]; HRMS: m/z calcd for $C_{17}H_{28}O_7$: 383.1466 [M+K]$^+$; found: 383.1469.

*6-O-Benzyl-1,2:3,4-di-O-isopropylidene-d-tagatofuranose (**12**)*

To a solution of diisopropylidene-D-tagatofuranose **10** [4] (1.8 g, 6.92 mmol) and BnBr (1.25 mL, 10.27 mmol) in THF (5 mL), 50% aqueous solution of NaOH (1.6 mL) was added followed by TBABr (250 mg, 0.692 mmol) and the resulting mixture was heated under reflux for 3 h. Then, the mixture was diluted with EtOAc (20 mL) and washed with 5% aqueous solution of citric acid (15 mL) and brine (15 mL). Organic layer was dried over Na_2SO_4, filtered and the solvent was evaporated under reduced pressure. The crude product was purified by column chromatography on silica gel using AcOEt/hexanes (1:10, v/v) as the eluent to afford 6-*O*-benzyl-D-tagatofuranose **12** (2.4 g, 91%) as a colorless oil. $[\alpha]_D$ = +22.1 (c=1.0, $CHCl_3$), ^{1}H NMR ($CDCl_3$): δ 7.48–7.02 (m, 5H, H_{Ar}), 4.84 (dd, J = 5.9, 3.8 Hz, 1H, H-4), 4.69 (d, J = 12.1 Hz, 1H, CH_2Ph), 4.64 (d, J = 5.8 Hz, 1H, H-3), 4.59 (d, J = 12.0 Hz, 1H, CH_2Ph), 4.31 (d, J = 9.6 Hz, 1H, H-1), 4.22 (ddd, J = 6.6, 5.0, 3.7 Hz, 1H, H-5), 4.10 (d, J = 9.7 Hz, 1H, H-1a), 3.86 (dd, J = 10.5, 5.0 Hz, 1H, H-6), 3.72 (dd, J = 10.6, 6.6 Hz, 1H, H-6a), 1.52, 1.44, 1.42 and 1.34 [4s, each 3H, $C(CH_3)_2$]; ^{13}C NMR ($CDCl_3$): δ 138.2, 128.6, 128.3, 127.7 and 127.5 (C_{Ar}), 112.7 [$C(CH_3)_2$], 111.8 [$C(CH_3)_2$], 111.6 (C-2), 85.1 (C-3), 80.2 (C-4), 78.4 (C-5), 73.3 (CH_2Ph), 69.2 (C-1), 67.9 (C-6), 26.54, 26.50, 26.0 and 25.0 [$C(CH_3)_2$]; HRMS: m/z calcd for $C_{19}H_{27}O_6$: 351.1802 [M+H]$^+$; found: 351.1810.

*1,2-Di-O-acetyl-6-O-benzyl-3,4-O-isopropylidene-β-D-tagatofuranose (**18**) and 1,2,6-tri-O-acetyl-3,4-O-isopropylidene-β-D-tagatofuranose (**19**)*

To a solution of diacetonide **12** (3.0 g, 8.56 mmol) in acetic anhydride (36 mL), $BF_3·OEt_2$ (110 µL, 0.85 mmol) was added as a solution in anhydrous CH_2Cl_2 (1 mL) at 0 °C over 5 min and the reaction mixture was stirred at 0 °C for 1.5 h [TLC, EtOAc/hexanes, (1:3, v/v)]. Then the mixture was poured into ice cold saturated solution of $NaHCO_3$ (50 mL) and was vigorously stirred at 0 °C for 2 h. The mixture was extracted with CH_2Cl_2 (3 × 20 mL). The combined extracts were washed with brine (2 × 10 mL), dried with Na_2SO_4, and filtered, and the solvent was evaporated under reduced pressure. Particular products were separated and purified by column chromatography on silica gel using EtOAc/hexanes (1:5→1:1, v/v) as the eluent to afford title compounds **18** and **19**.

Compound **18**: Yield 1.99 g, 59%, colorless oil, R_f = 0.36 (EtOAc/hexanes, 1:3), $[\alpha]_D$ = +55.4 (*c* =1, $CHCl_3$). ^1H NMR ($CDCl_3$): δ 7.40–7.30 (m, 5H, H_{Ar}), 4.97 (d, *J* = 6.0 Hz, 1H, H-3), 4.91 (dd, *J* = 6.0, 3.9 Hz, 1H, H-4), 4.70–4.52 (m, 4H, CH_2Ph, H-1, H-1a), 4.48 (ddd, *J* = 6.7, 5.0, 3.9 Hz, 1H, H-5), 3.81 (dd, *J* = 10.4, 5.1 Hz, 1H, H-6), 3.69 (dd, *J* = 10.4, 6.7 Hz, 1H, H-6a), 2.11 and 2.07 (2×$COCH_3$), 1.46 and 1.32 [2s, each 3H, $C(CH_3)_2$]; ^{13}C NMR ($CDCl_3$): δ 170.2 and 169.7 (2×$COCH_3$), 138.0, 128.3, 127.8 and 127.6 (C_{Ar}), 113.3 [$C(CH_3)_2$], 109.6 (C-2), 84.8 (C-3), 81.8 (C-5), 80.2 (C-4), 73.4 (CH_2Ph), 67.8 (C-6), 62.7 (C-1), 25.8 and 24.5 [$C(CH_3)_2$], 21.7 and 20.8 (2×$COCH_3$). HRMS: m/z calcd for $C_{20}H_{27}O_8$: 395.1700 $[M+H]^+$; found: 395.1698.

Compound **19**: Yield 0.81 g, 28%, colorless oil, R_f = 0.22 (EtOAc/hexanes, 1:3), $[\alpha]_D$ = +23.0 (*c* =1, $CHCl_3$) ^1H NMR ($CDCl_3$): δ 4.99 (d, *J* = 6.0 Hz, 1H, H-3), 4.94 (dd, *J* = 6.0, 4.0 Hz, 1H, H-4), 4.65 (d, *J* = 11.9 Hz, 1H, H-1), 4.57–4.42 (m, 3H, H-1a, H-5, H-

6), 4.20 (dd, *J* = 11.9, 7.3 Hz, 1H, H-6a), 2.12, 2.11 and 2.09 (3s, each 3H, COC*H*$_3$), 1.48 (s, 3H, CH$_3$), 1.33 (s, 3H, CH$_3$); ^{13}C NMR (CDCl$_3$): δ 170.8, 170.1 and 169.7 (3×*C*OCH$_3$), 113.6 [*C*(CH$_3$)$_2$], 109.7 (C-2), 84.9 (C-3), 80.8 (C-5), 80.2 (C-4), 62.8 (C-6), 62.7 (C-1), 25.8 and 24.4 [C(*C*H$_3$)$_2$], 21.7, 20.9 and 20.8 (3×*C*OCH$_3$). HRMS: m/z calcd for $C_{15}H_{23}O_9$: 347.1337 [*M*+H]$^+$; found: 347.1343.

*Ethyl 3,4-O-isopropylidene-6-O-pivaloyl-2-thio-α-D-tagatofuranoside (**13**)*

BF_3·Et_2O (179 μL, 1.45 mmol) was added to a stirred solution of methyl pivalate **11** (500 mg, 1.45 mmol) and ethanethiol (157 μL, 2.17 mmol) in anhydrous CH_2Cl_2 (10 mL) at 0 °C and the reaction mixture was stirred at this temperature until all starting material was consumed (2 h). Next, the reaction mixture was diluted with $CHCl_3$ (20 mL) and was washed with a saturated aqueous solution of $NaHCO_3$ (30 mL). The organic layer was dried with Na_2SO_4, and filtered, and the solvent was evaporated under reduced pressure. The crude product was purified by column chromatography with EtOAc/hexanes (1:4, v/v) as the eluent to afford 2-thio-α-D-tagatofuranoside **13** (298 mg, 59%) as a colorless oil. $[\alpha]_D$ = +124.6 (c = 1.75, CHCl$_3$), ^{1}H NMR (CDCl$_3$): δ 4.79 (dd, *J* = 6.0, 3.8 Hz, 1H, H-4), 4.49 (dd, *J* = 11.7, 3.6 Hz, 1H, H-6), 4.43 (d, *J* = 6.0 Hz, 1H, H-3), 4.33 (dt, *J* = 7.5, 3.7 Hz, 1H, H-5), 4.22 (dd, *J* = 11.7, 7.7 Hz, 1H, H-6a), 3.91 (d, *J* = 11.7 Hz, 1H, H-1), 3.75 (d, *J* = 11.7 Hz, 1H, H-1a), 2.51 (m, 2H, SC*H*$_2$CH$_3$), 1.48 and 1.29 [2s, each 3H, C(C*H*$_3$)$_2$],1.23 (t, *J* = 7.5 Hz, 3H, SCH$_2$C*H*$_3$), 1.19 [s, 9H, C(C*H*$_3$)$_3$]; ^{13}C NMR (100 MHz, CDCl$_3$): δ 178.1 (C=O), 113.1 [*C*(CH$_3$)$_2$], 96.7 (C-2), 85.7 (C-3), 80.4 (C-4), 77.9 (C-5), 61.98 (C-1), 61.92 (C-6), 38.6 [*C*(CH$_3$)$_3$], 27.0 [C(*C*H$_3$)$_3$], 25.7 and 24.4 [C(*C*H$_3$)$_2$], 21.2 (S*C*H$_2$CH$_3$), 15.1 (SCH$_2$*C*H$_3$); HRMS: m/z calcd for $C_{16}H_{28}O_6S$: 349.1679 [*M*+H]$^+$; found: 349.1682.

Ethyl 1-O-dibenzyloxyphosphoryl-3,4-O-isopropylidene-α-D-tagatofuranosyl sulfone (15)

To a solution of pivalate **14** (170 mg, 0.31 mmol) in MeOH (3 mL), a freshly prepared 1 M solution of NaOMe (160 µL) was added. The reaction mixture was stirred at room temperature for 90 min [TLC, EtOAc/hexanes, (1:1, v/v)]. Then, the mixture was carefully neutralized with Amberlite® IR-120 H^+. Next, the Amberlite was filtered off, washed with methanol (3 mL) and the filtrate was evaporated under reduced pressure. The crude product was purified by column chromatography with EtOAc/hexanes (1:1, v/v) as the eluent to afford alcohol **15** (137 mg, 93%) as a colorless oil. $[\alpha]_D$ = +24.9 (c = 1.0, $CHCl_3$), ^{1}H NMR ($CDCl_3$): δ 7.41–7.33 (m, 10H, H_{Ar}), 5.29 (d, *J* = 6.2 Hz, 1H, H-3), 5.18–5.02 (m, 4H, 2×C*H*$_2$Ph), 4.97 (dd, *J* = 6.2, 4.4 Hz, 1H, H-4), 4.69–4.50 (m, 2H, H-5, H-1), 4.42 (dd, *J* = 12.1, 6.4 Hz, 1H, H-1a), 3.85 (d, *J* = 5.6 Hz, 2H, H-6, H-6a), 3.30 (dq, *J* = 13.9, 7.6 Hz, 1H, C*H*$_2$CH$_3$), 3.00 (dq, *J* = 13.9, 7.6 Hz, 1H, C*H*$_2$CH$_3$), 1.46 and 1.32 [2s, each 3H, C(C*H*$_3$)$_2$]; ^{13}C NMR ($CDCl_3$): δ 135.57, 135.51, 128.73, 128.71, 128.6, 127.99 and 127.97 (C_{Ar}), 114.1 [*C*(CH$_3$)$_2$], 101.0 (C-2), 84.1 (C-5), 81.7 (C-3), 81.3 (C-4), 69.65 and 69.63, (2×*C*H$_2$Ph), 66.9 (C-1), 61.0 (C-6), 45.4 (*C*H$_2$CH$_3$), 25.6 and 23.9 [C(*C*H$_3$)$_2$], 5.03 (*C*H$_3$CH$_2$). HRMS: m/z calcd for $C_{25}H_{33}O_{10}PS$: 557.1605 $[M+H]^+$; found: 557.1611.

*General procedure for preparation of compounds **16** and **22***

To a solution of acetonids **15** (120 mg, 0.22 mmol), **21a** (112 mg, 0.17 mmol) and **21b** (115 mg, 0.17 mmol) in THF (4 mL), 3 M HCl (1.4 mL for **15** and 1.1 mL for **21a** and **21b**) was added dropwise and the reaction mixture was stirred at 40 °C for 8 h [TLC, EtOAc/hexanes, (6:1, v/v)]. Then, the solid $NaHCO_3$ was carefully added to neutralize the reaction and the solvents were evaporated under reduced pressure.

The crude products were purified by column chromatography with EtOAc/hexanes (6:1→EtOAc, v/v) as the eluent to afford triol **16** and diols **22**.

*Ethyl 1-O-dibenzyloxyphosphoryl-α-D-tagatofuranosyl sulfone (**16**)*

Yield 91 mg, 85 %, colorless oil, $[\alpha]_D$ = +2.1 (c = 1.0, MeOH), ^{1}H NMR (CD_3OD): δ 7.42–7.36 (m 10H, H_{Ar}), 5.13 (m, 4H, 2×CH_2Ph), 5.05 (d, *J* = 4.9 Hz, 1H, H-3), 4.68 (dd, *J* = 11.9, 5.7 Hz, 1H, H-1), 4.55 (dd, *J* = 11.9, 5.0 Hz, 1H, H-1a), 4.31 (dd, *J* = 4.9, 3.1 Hz, 1H, H-4), 4.24 (ddd, *J* = 6.5, 4.6, 3.1 Hz, 1H, H-5), 3.88 (dd, *J* = 11.9, 4.5 Hz, 1H, H-6), 3.80 (dd, *J* = 11.8, 6.5 Hz, 1H, H-6a), 3.38 (m, 1H, CH_2CH$_3$), 2.98 (m, 1H, CH_2CH$_3$), 1.30 (t, *J* = 7.5 Hz, 3H, CH$_2$CH_3); ^{13}C NMR (CD_3OD): δ 135.5, 128.38, 128.32 and 127.8 (C_{Ar}), 98.5 (C-2), 83.8 (C-5), 72.8 (C-3), 71.8 (C-4), 69.7 and 69.6 (2×CH$_2$Ph), 67.5 (C-1), 60.0 (C-6), 45.5 (CH$_2$CH$_3$), 4.12 (CH$_2$CH$_3$). HRMS: m/z calcd for $C_{22}H_{29}O_{10}PS$: 539.1115 $[M+Na]^+$; found: 539.1120.

*Phenyl 6-O-benzyl-1-O-dibenzyloxyphosphoryl-α-D-tagatofuranosyl sulfone (**22a**)*

Yield 80 mg, 78 %, colorless oil, $[\alpha]_D$ = +39.8 (c = 1.75, MeOH), ^{1}H NMR (CD_3OD): δ 7.99–7.89 (m, 2H, H_{Ar}), 7.61–7.54 (m, 1H, H_{Ar}), 7.46–7.40 (m, 2H, H_{Ar}), 7.36 (dt, *J* = 4.6, 2.1 Hz, 3H, H_{Ar}), 7.33–7.25 (m, 10H, H_{Ar}), 7.22–7.15 (m, 2H, H_{Ar}), 5.23 (d, *J* = 4.9 Hz, 1H, H-3), 4.84–4.71 (m, 3H, 2×CH_2Ph), 4.71–4.56 (m, 2H, H-1, CH_2Ph), 4.55–4.49 (m, 2H, H-1a, H-5), 4.47 (bs, 2H, CH_2Ph), 4.35 (dd, *J* = 4.9, 3.0 Hz, 1H, H-4), 3.84 (dd, *J* = 10.9, 3.7 Hz, 1H, H-6), 3.70 (dd, *J* = 10.9, 7.3 Hz, 1H, H-6a); ^{13}C NMR (CD_3OD): δ 138.1, 137.6, 135.6, 135.5, 133.6, 130.1, 128.3, 128.27, 128.24, 128.1, 127.9, 127.6, 127.5, 127.2 (C_{Ar}), 99.6 (C-2), 82.5 (C-5), 73.4 (C-3), 72.8, (CH$_2$Ph), 69.3 (C-4), 69.24 and 69.22 (2×CH$_2$Ph), 68.5 (C-6), 66.5 (C-1). HRMS: m/z calcd for $C_{33}H_{36}O_8PS$: 623.1863 $[M+H]^+$; found: 623.1825.

*Isopropyl 6-O-benzyl-1-O-dibenzyloxyphosphoryl-α-D-tagatofuranosyl sulfone (**22b**)*

Yield 77 mg, 74 %, colorless oil, $[\alpha]_D$ = +43.2 (c = 0.1, MeOH), 1H NMR (400 MHz, CD_3OD): δ 7.44–7.20 (m, 15H, H_{Ar}), 5.19–5.05 (m, 5H, 2×CH_2Ph, H-3), 4.70 (dd, *J* = 12.0, 6.1 Hz, 1H, H-1), 4.56–4.47 (m, 3H, CH_2Ph, H-1a), 4.39 (ddd, *J* = 7.0, 4.0, 2.9 Hz, 1H, H-5), 4.25 (dd, *J* = 4.9, 2.9 Hz, 1H, H-4), 3.82 (dd, *J* = 10.8, 4.0 Hz, 1H, H-6), 3.70 (dd, *J* = 10.8, 7.2 Hz, 1H, H-6a), 3.53 [p, *J* = 6.8 Hz, 1H, C*H*(CH_3)$_2$], 1.40 [d, *J* = 6.7 Hz, 3H, CH(CH_3)$_2$], 1.28 [d, *J* = 7.0 Hz, 3H, CH(CH_3)$_2$]; ^{13}C NMR (101 MHz, CD_3OD): δ 138.1, 128.3, 128.3, 127.9, 127.79, 127.70 and 127.2 (C_{Ar}), 100.4 (C-2), 82.3 (C-5), 73.6 (C-3), 72.7 (*C*H_2Ph), 71.6 (C-4), 69.7, 69.49 (2×*C*H_2Ph), 68.4 (C-6), 67.5 (C-1), 54.7 [*C*H(CH_3)$_2$], 16.62 and 13.85 [CH(*C*H_3)$_2$]. HRMS: m/z calcd for $C_{30}H_{37}O_{10}PS$: 659.1477 [*M*+K]$^+$; found: 659.1472.

*General procedure for th preparation of target compounds **2** and **3***

A mixture of alcohol **15**, triol **16** or compounds **21** and **22** (1 equiv) and Pd/C (10%, 50 mg) in MeOH (8 mL per 100 mg of substrate) was stirred at room temperature under a hydrogen atmosphere for 3–4 h [TLC, $CHCl_3$/MeOH, (3:1, v/v)]. When the reaction was complete, the mixture was filtered through a pad of Celite® and a filter cake was washed with a mixture of MeOH/distilled water [2:1 (v/v), 7 mL]. The filtrate was evaporated under reduced pressure and the residue was dissolved in distilled water (5 mL). The resulting mixture was filtered through a PTFE syringe filter (0.45 μm) and the filtrate was lyophilized to afford compounds **2** and **3**.

*Ethyl 1-O-phosphono-α-D-tagatofuranosyl sulfone (**2a**)*

Yield 15 mg, 98 %, white powder, $[\alpha]_D$ = +10.7 (c = 1.0, H_2O), ^{1}H NMR **(**CD_3OD): δ 5.03 (m, 1H, H-3), 4.52-4.40 (m, 2H, H-1, H-1a), 4.34 (s, 1H, H-5), 4.24 (s, 1H, H-4),

3.91-3.80 (m, 2H, H-6, H-6a), 3.51 (s, 1H, CH_2CH_3), 3.25 (s, 1H, CH_2CH_3), 1.38 (s, 3H, CH_2CH_3); ^{13}C NMR (CD_3OD): δ 99.5 (C-2), 83.4 (C-4), 72.6 (C-3), 71.7 (C-5), 65.9 (C-1), 60.1 (C-6), 45.6 (CH_2CH_3), 4.1 (CH_2CH_3). HRMS: m/z calcd for $C_8H_{17}O_{10}PS$: 359.0172 [M+Na]$^+$; found: 359.0175.

*Phenyl 1-O-phosphono-α-D-tagatofuranosyl sulfone (**2b**)*

Yield 17 mg, 96 %, white powder, $[\alpha]_D$ = +6.9 (c = 1.0, H_2O), ^{1}H NMR (CD_3OD): δ 7.99 (d, J = 7.7 Hz, 1H, H_{Ar}), 7.63 (t, J = 7.6 Hz, 2H, H_{Ar}), 7.49–7.37 (m, 2H, H_{Ar}), 5.10 (d, J = 5.0 Hz, 1H, H-3), 4.31 (d, J = 5.1 Hz, 1H, H-5), 4.25 (p, J = 5.1, 4.6 Hz, 2H, H-4, H-1), 4.09–4.01 (m, 1H, H-1a), 3.89-3.80 (m, 2H, H-6, H-6a). ^{13}C NMR (CD_3OD): δ 136.7, 132.7, 130.1, 128.7 (C_{Ar}), 101.9 (C-2), 84.8 (C-4), 74.8 (C-3), 72.4 (C-5), 65.2 (C-1), 61.1 (C-6). HRMS: m/z calcd for $C_{12}H_{17}O_8PS$: 375.0274 [M+H]$^+$; found: 375.0275.

*Isopropyl 1-O-phosphono-α-D-tagatofuranosyl sulfone (**2c**)*

Yield 14 mg, 95 %, white powder, $[\alpha]_D$ = +9.8 (c = 1.0, H_2O), ^{1}H NMR (CD_3OD): δ 4.98 (m, 1H, H-3), 4.58-4.42 (m, 2H, H-1, H-1a), 4.37–4.30 (m, 1H, H-4), 4.30–4.22 (m, 1H, H-5), 3.92 [p, J = 12.1, 4.4 Hz, 1H, CH(CH_3)$_2$], 3.83 (m, 2H, H-6, H-6a), 1.48 (d, J = 6.7 Hz, 3H, CH_3), 1.40 (d, J = 6.8 Hz, 3H, CH_3). ^{13}C NMR (CD_3OD): δ 101.9 (C-2), 83.1 (C-5), 73.4 (C-3), 71.5 (C-3), 64.6 (C-1), 59.8 (C-6), 54.4 [CH(CH_3)$_2$], 16.5 and 13.9 [CH(CH_3)$_2$]. HRMS: m/z calcd for $C_9H_{19}O_{10}PS$: 373.0329 [M+Na]$^+$; found: 373.0325.

*Ethyl 3,4-O-isopropylidene-1-O-phosphono-α-D-tagatofuranosyl sulfone (**3a**)*

Yield 16 mg, 98 %, white powder, $[\alpha]_D$ = +3.1 (c = 0.5, H_2O), ^{1}H NMR (CD_3OD): δ 5.34–5.17 (m, 1H, H-3), 4.96 (m, 1H, H-4), 4.56 (s, 1H, H-5), 4.44 (bs, 1H, H-1), 4.27 (d, *J* = 14.9 Hz, 1H, H-1a), 3.85 (d, *J* = 11.0 Hz, 1H, H-6), 3.79–3.68 (m, 1H, H-6a), 3.53 (s, 1H, CH_2CH_3), 3.28–3.19 (m, 1H, CH_2CH_3), 1.49 (s, 3H CH_2CH_3), 1.36 (bs, J = 10.6 Hz, 6H, $[C(CH_3)_2]$; ^{13}C NMR (CD_3OD): δ 113.6 $[C(CH_3)_2]$, 101.5 (C-2), 84.7 (C-5), 81.5 (C-3), 80.9 (C-4), 65.8 (C-1), 60.1 (C-6), 45.4 (CH_2CH_3), 24.6 and 22.90 $[C(CH_3)_2]$, 3.95 (CH_2CH_3). HRMS: m/z calcd for $C_{11}H_{21}O_{10}PS$: 399.0485 $[M+Na]^+$; found: 399.0490.

*Phenyl 3,4-O-isopropylidene-1-O-phosphono-α-D-tagatofuranosyl sulfone (**3b**)*

Yield 17 mg, 98 %, white powder, $[\alpha]_D$ = +2.8 (c = 1.0, H_2O), ^{1}H NMR (CD_3OD): δ 7.98 (d, *J* = 7.3 Hz, 2H, H_{Ar}), 7.71 (t, *J* = 6.9 Hz, 1H, H_{Ar}), 7.60 (d, *J* = 7.4 Hz, 2H, H_{Ar}), 5.49 (d, *J* = 5.9 Hz, 1H, H-3), 5.10 (s, 1H, H-4), 4.80 (s, 1H, H-5), 4.43–4.36 (m, 1H, H-1), 4.16–4.06 (m, 1H, H-1a), 3.93–3.81 (m, 1H, H-6), 3.70 (t, *J* = 9.4 Hz, 1H, H-6a), 1.49 and 1.38 $[C(CH_3)_2]$; ^{13}C NMR (CD_3OD): δ 136.2, 133.8, 130.3 and 128.4 (C_{Ar}), 113.6 $[C(CH_3)_2]$, 102.2 (C-2), 84.9 (C-5), 81.9 (C-3), 81.1 (C-4), 64.5 (C-1), 60.1 (C-6), 24.5 and 22.9 $[C(CH_3)_2]$. HRMS: m/z calcd for $C_{15}H_{21}O_8PS$: 415.0587 $[M+Na]^+$; found: 415.0588.

*Isopropyl 3,4-O-isopropylidene-1-O-phosphono-α-D-tagatofuranosyl sulfone (**3c**)*

Yield 12 mg, 95 %, white powder, $[\alpha]_D$ = +14.1 (c = 0.5, H_2O), ^{1}H NMR (CD_3OD): δ 5.30 (d, *J* = 5.9 Hz, 1H, H-3), 4.96 (m, 1H, H-4), 4.61 (dt, *J* = 7.8, 4.0 Hz, 1H, H-5), 4.44 (d, *J* = 10.4 Hz, 1H, H-1), 4.30 (d, *J* = 9.9 Hz, 1H, H-1a), 3.98–3.88 [m, 1H, $CH(CH_3)_2$], 3.84 (dd, *J* = 12.0, 4.1 Hz, 1H, H-6), 3.74 (dd, *J* = 11.9, 7.4 Hz, 1H, H-6a),

1.50 (bs, 6H, 2× CH_3), 1.36 (bs 6H, 2×CH_3). ^{13}C NMR (CD_3OD): δ 113.5 [*C*(CH_3)$_2$], 103.7 (C-2), 84.6 (C-5), 82.4 (C-3), 80.7 (C-4), 65.8 (C-1), 60.1 (C-6), 55.2 [*C*H(CH_3)$_2$], 24.5 and 22.9 [C(*C*H_3)$_2$], 16.3 and 13.8 [CH(*C*H_3)$_2$]. HRMS: m/z calcd for $C_{12}H_{23}O_{10}PS$: 413.0642 [*M*+Na]$^+$; found: 413.0649.

General procedure for the thioglycosylation of diacetonide ***12***

$BF_3·Et_2O$ (1.8 equiv.) was added to a stirred solution of diacetonide **12** (1 eq.) and corresponding thiol [3 equiv, (PhSH, iPrSH)] in anhydrous CH_2Cl_2 (5 mL per 100 mg) at −5 °C and the reaction mixture was stirred at this temperature until all starting material was consumed (1 h). Next, the reaction mixture was washed with saturated aqueous solution of $NaHCO_3$ (20 mL per 100 mg) and brine (20 mL per 100 mg). The organic layer was dried with Na_2SO_4, and filtered, and the solvent was evaporated under reduced pressure. The crude products were purified by column chromatography with EtOAc/hexanes (1:5, v/v) as the eluent to afford 2-thio-α-D-tagatofuranosides **17**.

*Phenyl 6-O-benzyl-3,4-O-isopropylidene-2-thio-α-D-tagatofuranoside (**17a**)*

Yield 380 mg, 44 %, colorless oil, $[α]_D$ = +107.0 (c = 1.0, $CHCl_3$), ^1H NMR ($CDCl_3$): δ 7.59–7.44 (m, 2H, H_{Ar}), 7.44–7.24 (m, 8H, H_{Ar}), 4.91 (dd, *J* = 6.0, 3.8 Hz, 1H, H-4), 4.77 (dt, *J* = 7.0, 4.1 Hz, 1H, H-5), 4.67 (d, *J* = 12.0 Hz, 1H, C*H*$_2$Ph), 4.63 (d, *J* = 6.0 Hz, 1H, H-5), 4.57 (d, *J* = 12.0 Hz, 1H, C*H*$_2$Ph), 3.96 (dd, *J* = 10.7, 4.4 Hz, 1H, H-6), 3.85–3.74 (m, 2H, H-1, H-6a), 3.57 (d, *J* = 11.8 Hz, 1H, H-1a), 1.50 and 1.36 [C(C*H*$_3$)$_2$]; ^{13}C NMR ($CDCl_3$): δ 138.2, 135.9, 129.2, 129.0, 128.8, 128.3, 127.6 and 127.5 (C_{Ar}), 113.1 [*C*(CH_3)$_2$], 99.4 (C-2), 85.6 (C-3), 80.6 (C-4), 79.5 (C-5), 73.3

(*C*H_2Ph), 67.6 (C-6), 61.5 (C-1), 25.8 and 24.5 [C(*C*H_3)$_2$]. HRMS: m/z calcd for $C_{22}H_{26}O_5S$: 425.1393 [*M*+Na]$^+$; found: 425.1395.

*Isopropyl 6-O-benzyl-3,4-O-isopropylidene-2-thio-α-D-tagatofuranoside (**17b**)*

Yield 255 mg, 54 %, colorless oil, $[\alpha]_D$ = +95.2 (c = 1.0, $CHCl_3$), ^{1}H NMR ($CDCl_3$): δ 7.38–7.25 (m, 5H, H_{Ar}), 4.80 (dd, *J* = 6.0, 3.9 Hz, 1H, H-4), 4.65 (d, *J* = 12.1 Hz, 1H, C*H_2*Ph), 4.55 (d, *J* = 12.1 Hz, 1H, C*H_2*Ph), 4.43 (dt, J = 6.0, 3.8 Hz, 1H, H-5), 4.40 (d, *J* = 6.0 Hz, 1H, H-3), 3.96 (dd, *J* = 11.6, 10.5 Hz, 1H, H-1), 3.88 (dd, *J* = 10.7, 4.7 Hz, 1H, H-6), 3.79 (dd, *J* = 11.6, 3.2 Hz, 1H, H-1a), 3.75 (dd, *J* = 10.7, 7.0 Hz, 1H, H-6a), 3.10 [p, *J* = 6.9 Hz, 1H, C*H*(CH_3)$_2$], 2.51 (dd, *J* = 10.5, 3.2 Hz, 1H, OH), 1.48 and 1.31 [C(C*H_3*)$_2$], 1.33 and 1.32 [2d, *J* = 6.9 Hz, each 3H, CH(C*H_3*)$_2$]; ^{13}C NMR ($CDCl_3$): δ 138.1, 128.2 and 127.5 (C_{Ar}), 112.8 [*C*(CH_3)$_2$], 97.6 (C-2), 85.7 (C-3), 80.5 (C-4), 79.2 (C-5), 73.3 (*C*H_2Ph), 67.5 (C-6), 61.8 (C-1), 32.8 [*C*H(CH_3)$_2$], 25.7 [CH(*C*H_3)$_2$], 25.1 and 24.4 [C(*C*H_3)$_2$]. HRMS: m/z calcd for $C_{19}H_{28}O_{5S}$: 391.1550 [*M*+Na]$^+$; found: 391.1555.

*Phenyl 1-O-acetyl-6-O-benzyl-3,4-O-isopropylidene-2-thio-α-D-tagatofuranoside (**20**)*

To a solution of diacetate **18** (1.5 g, 3.81 mmol) and PhSH (550 µL, 5.33 mmol) in anhydrous $CHCl_3$ (12 mL), $BF_3.OEt_2$ (1.5 mL, 11.4 mmol) was added dropwise at −20 °C and the reaction mixture was stirred for 90 min at this temperature [TLC, EtOAc/hexanes, (2:1, v/v)]. Then the mixture was diluted with $CHCl_3$ (50 mL) and washed with saturated solution of $NaHCO_3$ (2 × 25 mL) and brine (2× 15 mL). The organic layer was dried with Na_2SO_4, and filtered, and the solvent was evaporated under reduced pressure. The crude product was purified by column chromatography with EtOAc/hexanes (1:4, v/v) as the eluent to afford 2-thio-α-D-tagatofuranoside **20**

(1.2 g, 72%) as a colorless oil. $[\alpha]_D^{20}$ = +77.1 (c = 1.0, $CHCl_3$), ^{1}H NMR ($CDCl_3$): δ 7.61–7.43 (m, 2H, H_{Ar}), 7.44–7.14 (m, 8H, H H_{Ar}), 4.91 (dd, *J* = 6.0, 3.8 Hz, 1H, H-4), 4.76 (dt, *J* = 7.0, 4.2 Hz, 1H, H-5), 4.65 (d, *J* = 12.1 Hz, 1H, CH_2Ph), 4.60 (d, *J* = 6.0 Hz, 1H, H-3), 4.56 (d, *J* = 12.0 Hz, 1H, CH_2Ph), 4.24 (m, 2H, H1, H-1a), 3.95 (dd, *J* = 10.8, 4.4 Hz, 1H, H-6), 3.78 (dd, *J* = 10.8, 7.1 Hz, 1H, H-6a), 2.10 (s, 3H, COCH_3), 1.46 and 1.35 [2s, each 3H, C(CH_3)$_2$]; ^{13}C NMR ($CDCl_3$): δ 170.2 (*C*OCH_3), 138.3, 135.9, 129.7, 128.9, 128.7, 128.3, 127.59 and 127.54 (C_{Ar}), 113.10 [*C*(CH_3)$_2$], 96.7 (C-2), 85.5 (C-3), 80.8 (C-4), 79.5 (C-5), 73.3 (*C*H_2Ph), 67.6 (C-6), 62.5 (C-1), 25.8 and 24.8 [C(*C*H_3)$_2$], 21.00 (CO*C*H_3). HRMS: m/z calcd for $C_{24}H_{28}O_6S$: 467.1499 [*M*+Na]$^+$; found: 467.1501.

Biological assays

Preparation of enzymatically active cell envelope fraction of Mycobacterium smegmatis mc^{2}155

M. smegmatis mc^{2}155 were grown in nutrient broth at 37 °C, 120 min^{-1}. The cells were suspended in 50 mM MOPS buffer (pH 7.9) containing 5 mM β-mercaptoethanol and 10 mM $MgCl_2$ (Buffer A) in the ratio 3 g/8 mL of the final suspension, and the mixture was subjected to probe sonication (Soniprep 150; Sanyo, MSE Ltd., Sussex, United Kingdom) 10 times 60 s pulses with 90 s cooling intervals. The lysate was centrifuged at 100 000 × g for 60 min at 4 °C. The pellet was resuspended in buffer A (250 μL for 1 mL of the centrifuged suspension) to obtain the enzymatically active fraction containing mycobacterial membrane and cell wall proteins (L100) with a concentration of ≈ 20 mg/mL.

Reaction mixtures and analysis of reaction products

Enzyme reactions, extractions and analyses of the reaction products were carried out as described by Mikusova et al. [5] with minor modifications. 250–500 μg of L100 proteins were pre-incubated 10 min on ice with the inhibitors at the final concentration in the reaction mixture of 0.5–10 mM. The reaction mixture was supplemented with UDP-GlcNAc (200 μM), TDP-Rha (200 μM), NADH (2.5 mM) and UDP-[^{14}C]Gal*p* (0.125 μCi, specific activity 55 mCi/mmol, ARC) for monitoring of the incorporation of the radioactive substrate into the lipid-linked intermediates of galactan synthesis. The volume of the reaction was adjusted to 40 μL with Buffer A. The mixtures were incubated for 1 h at 37 °C and stopped by addition of 1.5 mL $CHCl_3$-CH_3OH (2:1; v/v). The suspensions were rocked at room temperature for 20 min and centrifuged. The supernatant, $CHCl_3$-CH_3OH phase containing lower lipid-linked galactan precursors (GL3–5) was removed from the pellet and subjected to Folch wash, as described previously [5]. The final $CHCl_3$-CH_3OH phase was dried and dissolved in 40–50 μL of $CHCl_3$/CH_3OH/H_2O/NH_4OH (65:25:3.6:0.5); 10 μL were subjected to scintillation counting and 10 μl were analysed by thin layer chromatography on silica gel plates [Silica gel 60 F_{254} (Merck)] in $CHCl_3$-CH_3OH-NH_4OH-1 M ammonium acetate-H_2O (180:140:9:9:23). The radiolabeled lipid bands were visualized by autoradiography.

The pellets were extracted with 50% CH_3OH in H_2O with 0.9% NaCl, 50% CH_3OH in H_2O and 100% CH_3OH (1 mL each), as described previously [5], to remove the residual radiolabel. The lipid-linked galactan polymer was obtained by the extraction of the resulting pellets with 1 ml of “TT3” $CHCl_3$-CH_3OH-H_2O (10:10:3; v/v) and then with 1 ml of “E-soak” (water-ethanol-diethyl ether-pyridine-concentrated ammonium

hydroxide [15:15:5:1:0.017]) [5]. 10-20% of these fractions were then subjected to quantification by scintillation counting.

References

1. Schrödinger Suite 2018.04, 2018; Schrödinger, LLC: New York, NY, 2018.
2. Janoš, P.; Kozmon, S.; Tvaroška, I.; Koča, J. *CHEMISTRY A Eur. J.* **2018**, *24*, 7051-7059.
3. Jakubčinová, J.; Kozmon, S.; Šesták, S.; Baráth, M. *Chem. Select*. **2020**, *5*, 4967-4972. DOI: 10.1002/slct.202001098.
4. Yoshihara, A.; Haraguchi, S.; Gullapalli, P.; Rao, D.; Mori-moto, K.; Takata, G.; Jones, N.; Jenkinson S. F.; Wormald. M. R.; Dwek, R. A.; Fleet, G. W. J.; Izumori, K. *Tetrahedron: Asymmetr.* **2008**, *19*, 739–745.
5. Mikušová, K.; Yagi, T.; Stern, R.; McNeil, M. R.; Besra ,G. S.; Crick, D. C.; Brennan. P. J. *J. Biol. Chem*. **2000**, *275*, 33890-33897.

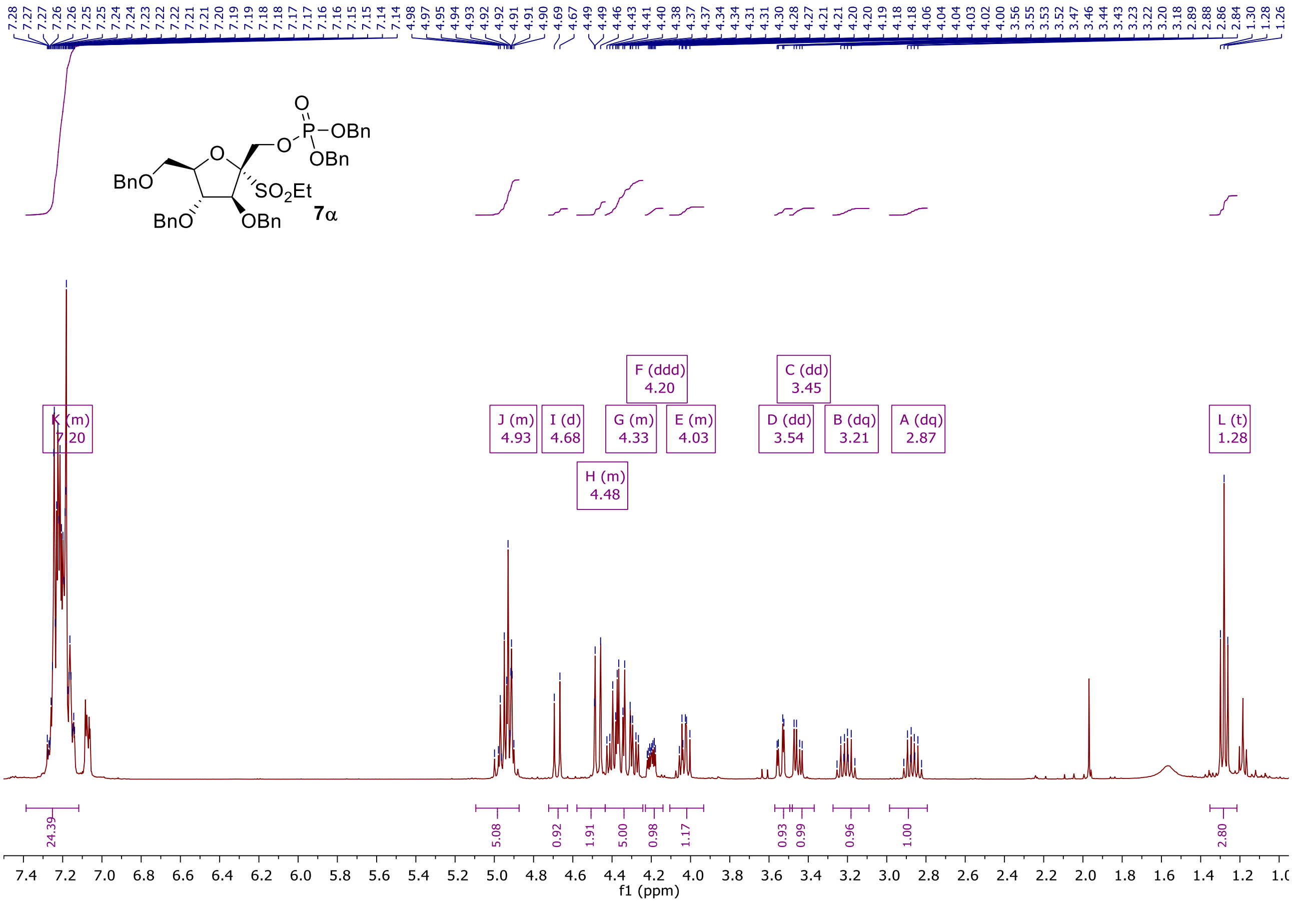
O
P
OBn
OBn
O
BnO
O
SO2Et
BnO
OBn
7α
F (ddd) 4.20
C (dd) 3.45
K (m) 7.20
J (m) 4.93
I (d) 4.68
G (m) 4.33
E (m) 4.03
D (dd) 3.54
B (dq) 3.21
A (dq) 2.87
L (t) 1.28
H (m) 4.48
f1 (ppm)

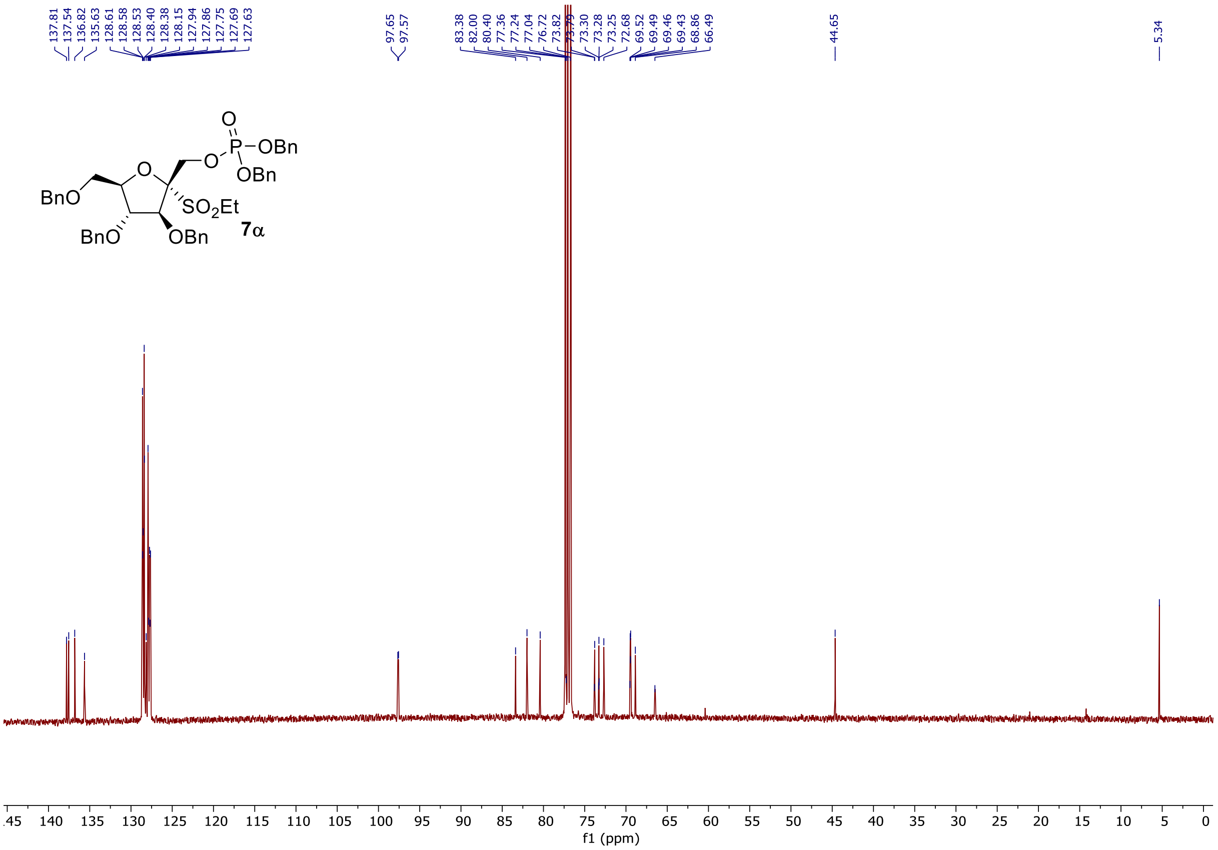
O
P
OBn
OBn
O
BnO
O
SO2Et
BnO
OBn
7α
f1 (ppm)

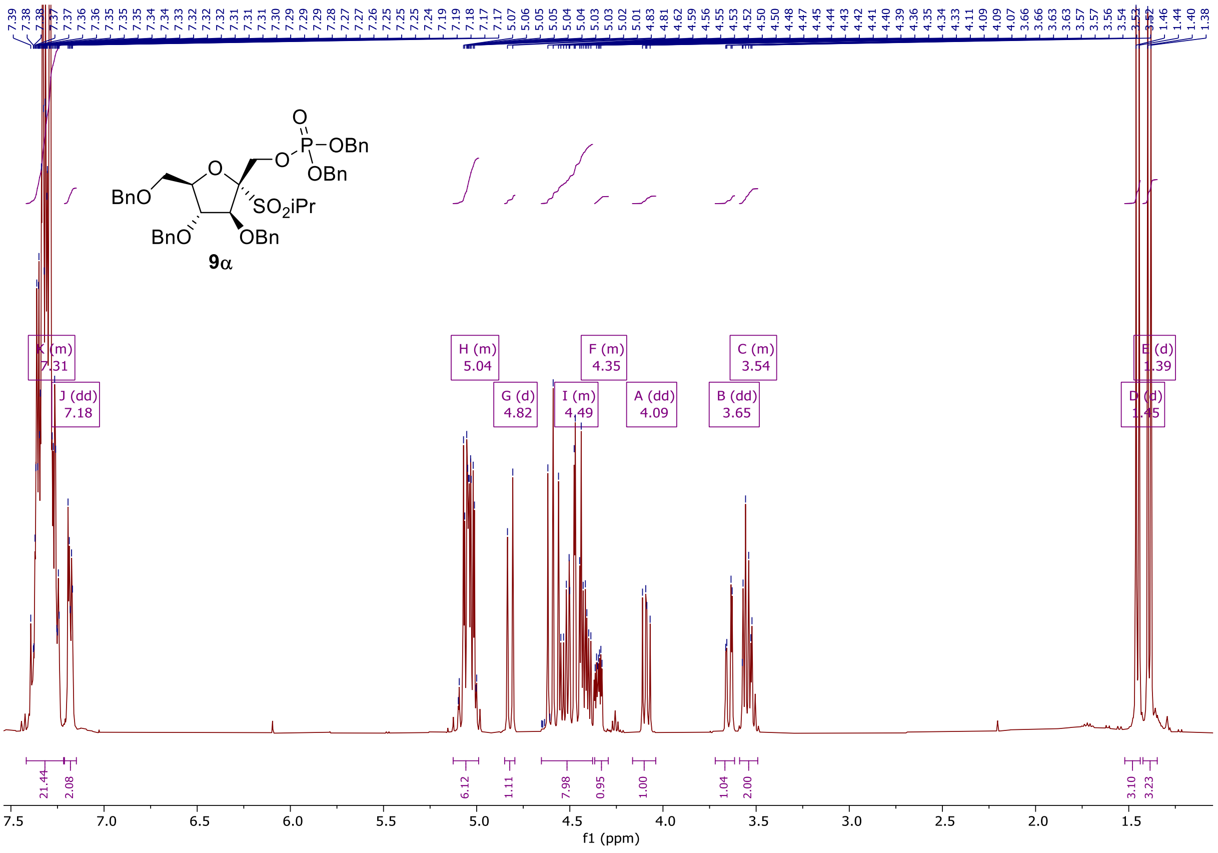

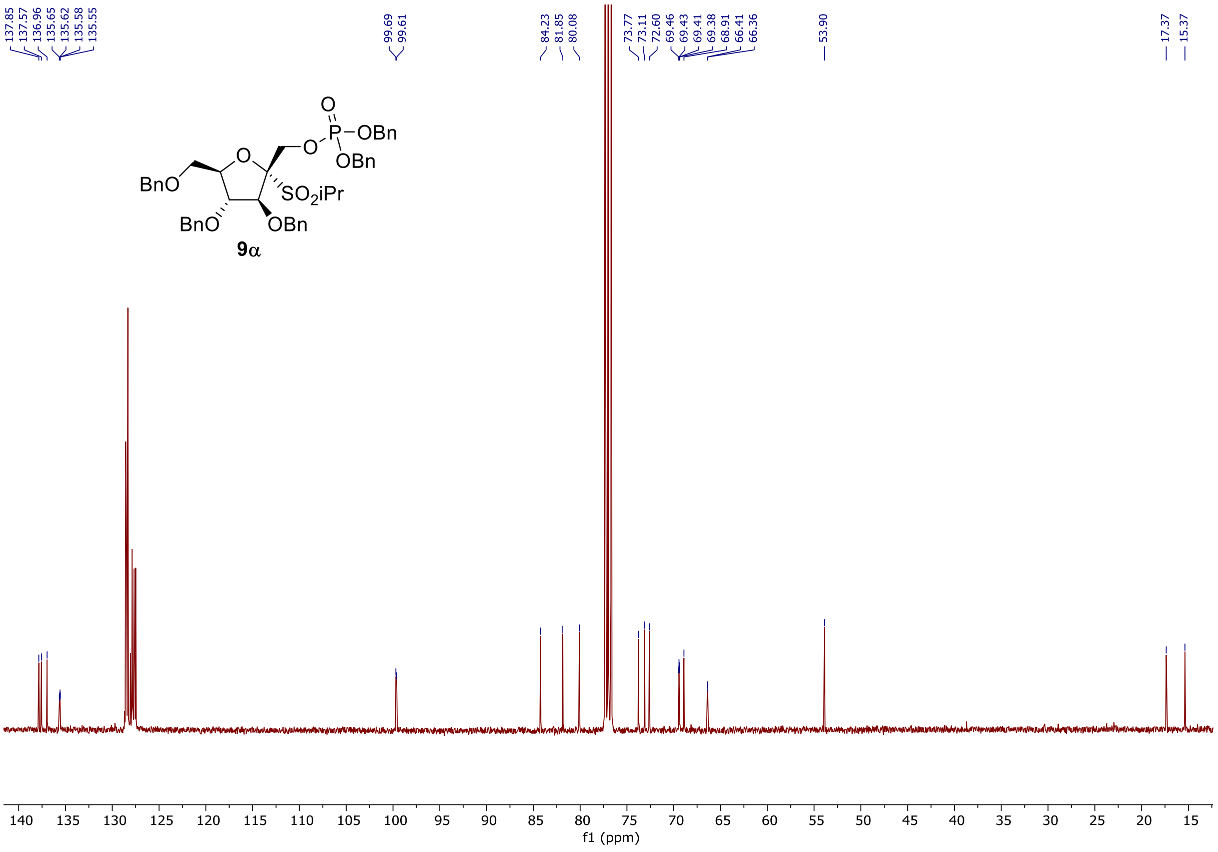

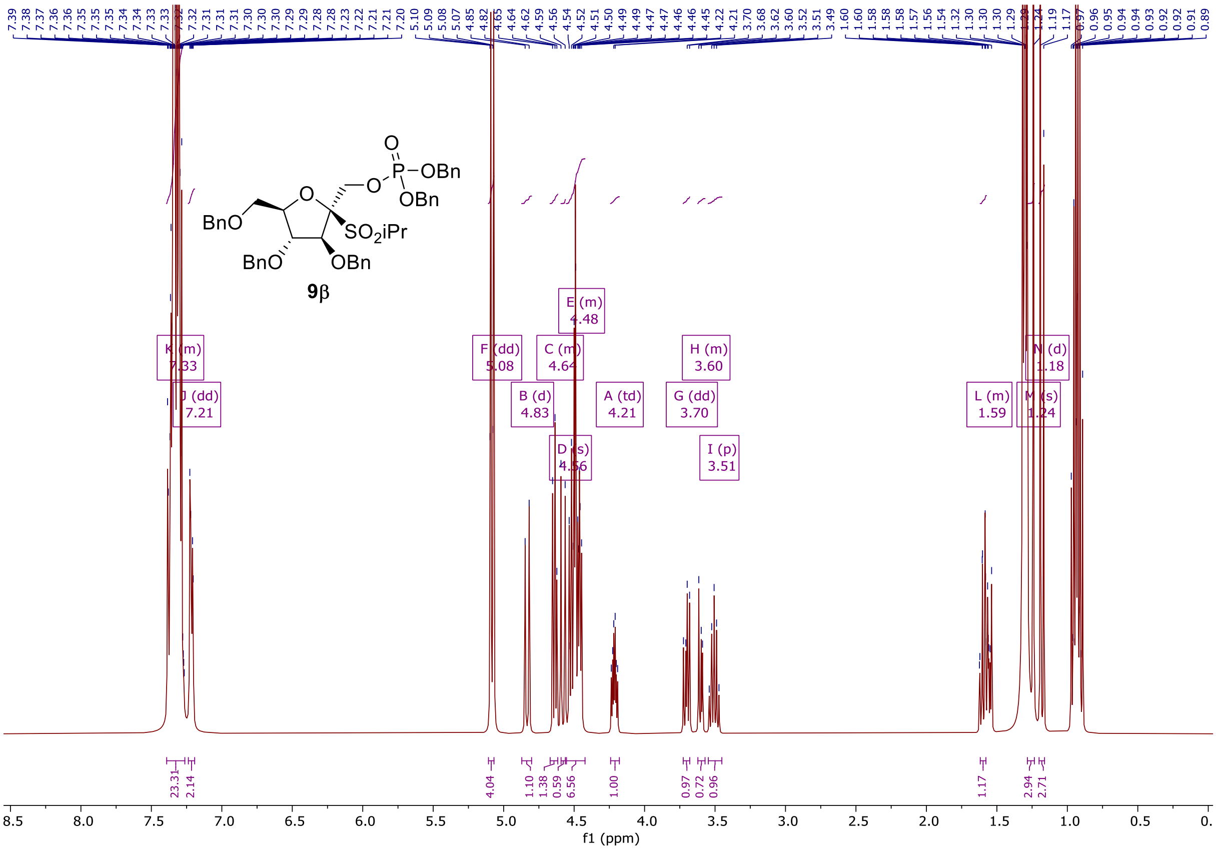

BnO
BnO
OBn
OBn
OBn
SO2iPr
9β
K (m) 7.33
J (dd) 7.21
F (dd) 5.08
B (d) 4.83
C (m) 4.64
D (s) 4.56
E (m) 4.48
A (td) 4.21
G (dd) 3.70
H (m) 3.60
I (p) 3.51
L (m) 1.59
M (s) 1.24
N (d) 1.18
f1 (ppm)

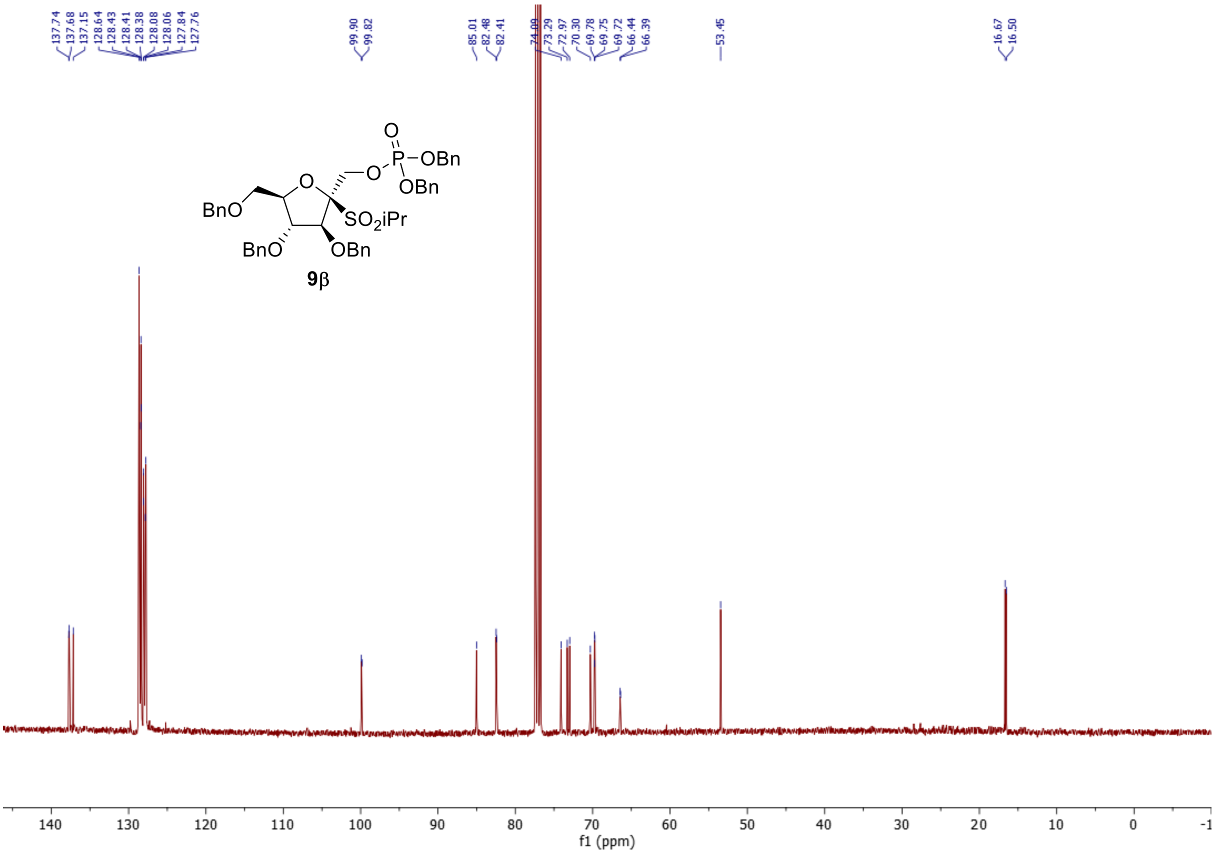

137.74
137.68
137.15
128.64
128.43
128.41
128.38
128.08
128.06
127.84
127.76
99.90
99.82
85.01
82.48
82.41
74.09
73.29
72.97
70.30
69.78
69.75
69.72
66.44
66.39
53.45
16.67
16.50
BnO
BnO
OBn
OBn
OBn
SO2iPr
9β
f1 (ppm)

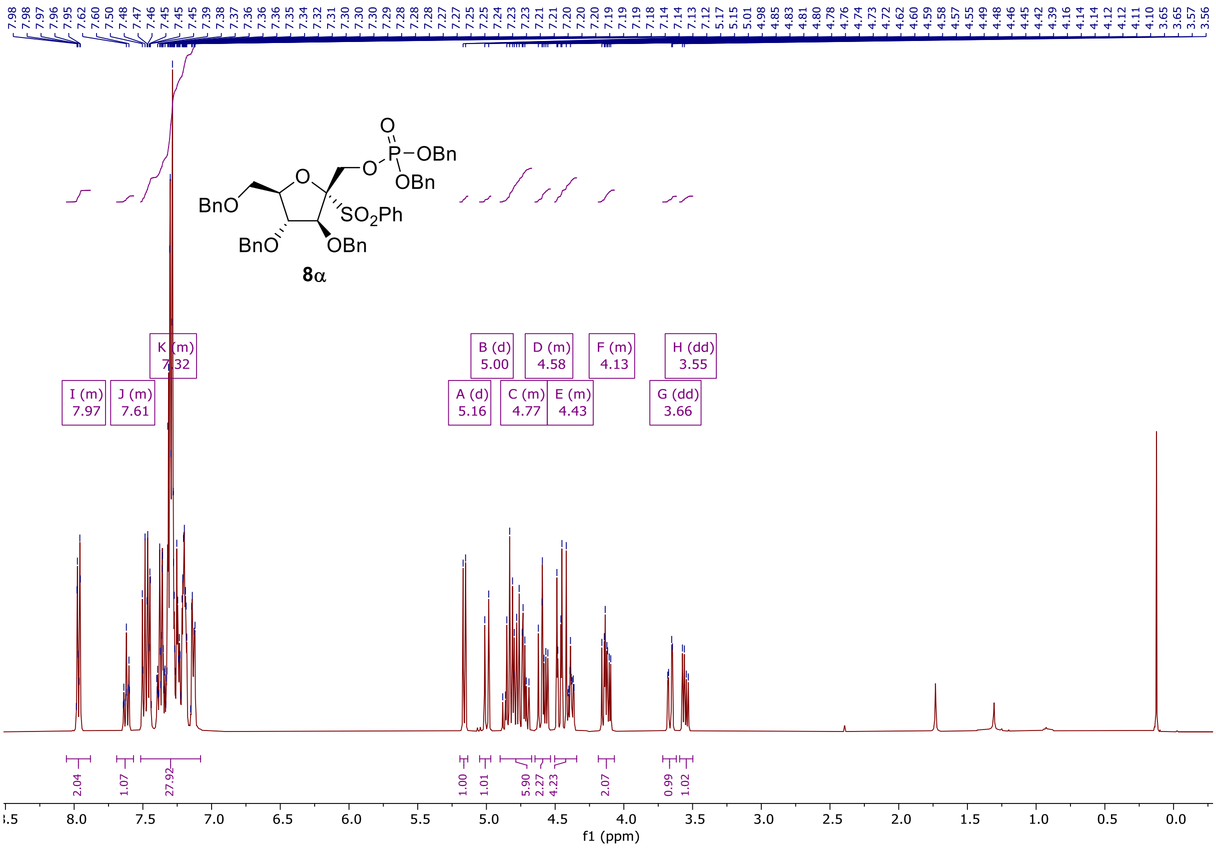

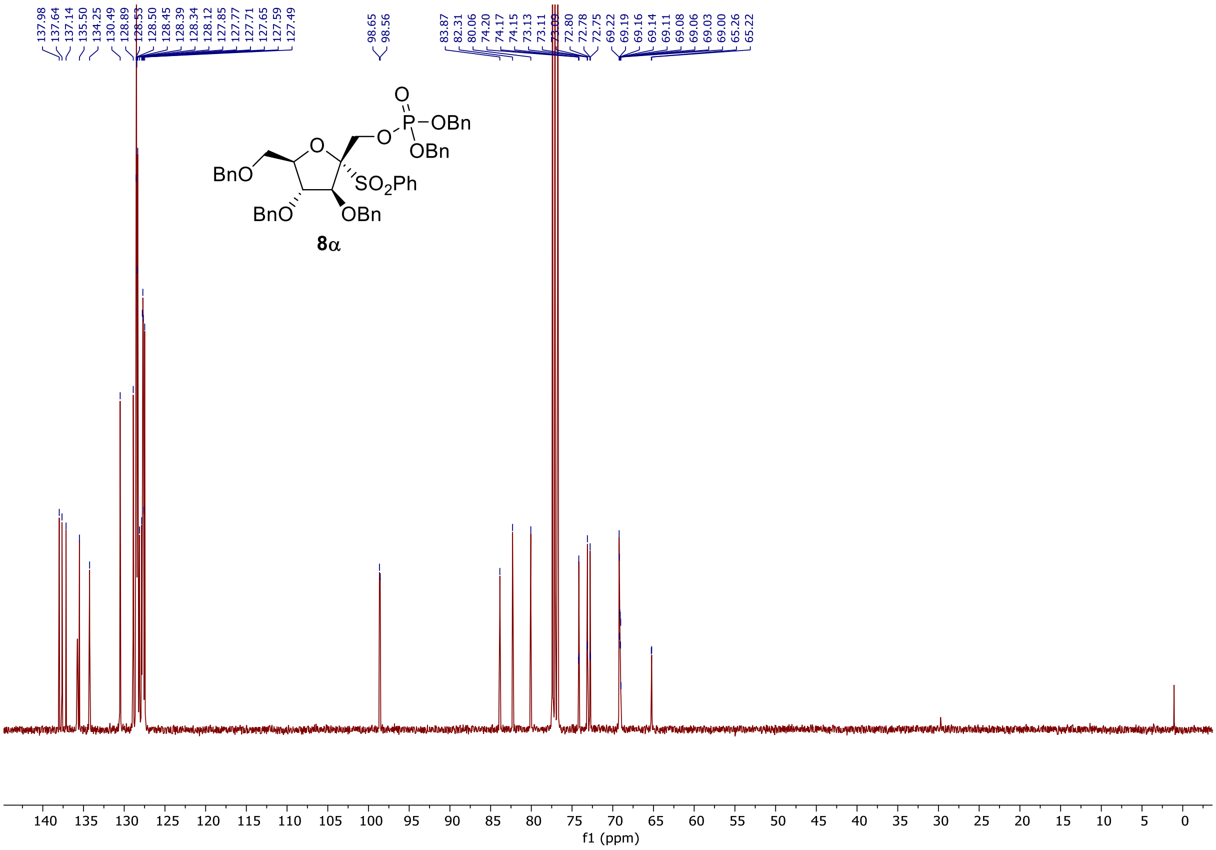

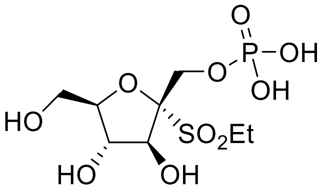

O
P
OH
OH
O
O
HO
SO2Et
HO
OH

1aα

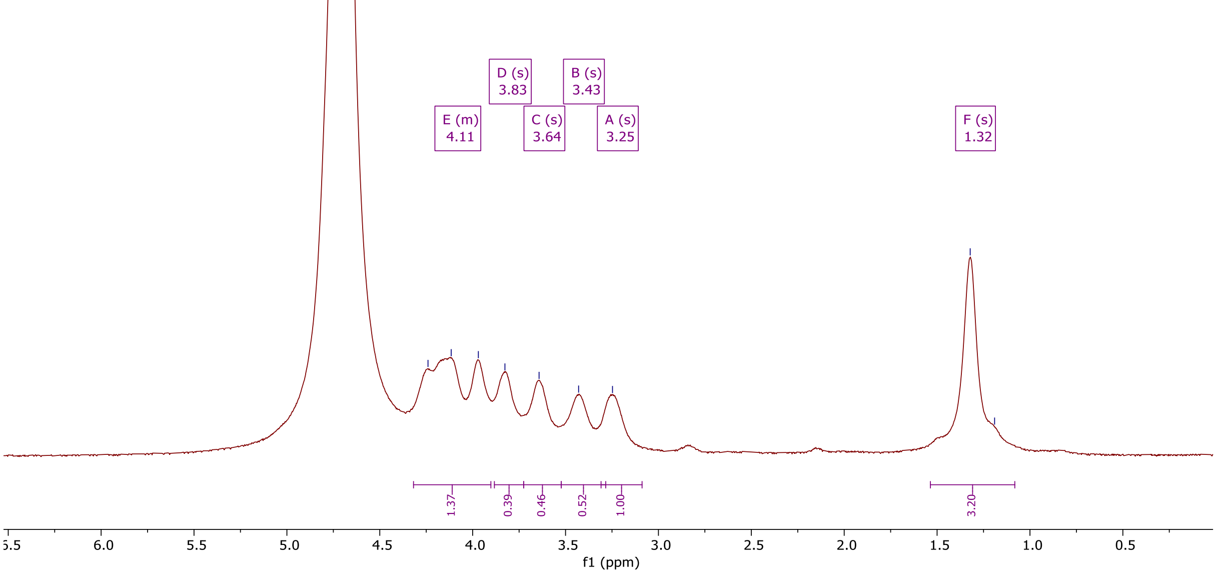

4.24
4.12
3.97
3.83
3.64
3.43
3.25
1.32
1.19
D (s) 3.83
B (s) 3.43
E (m) 4.11
C (s) 3.64
A (s) 3.25
F (s) 1.32
1.37
0.39
0.46
0.52
1.00
3.20
6.0
5.5
5.0
4.5
4.0
3.5
3.0
2.5
2.0
1.5
1.0
0.5
f1 (ppm)

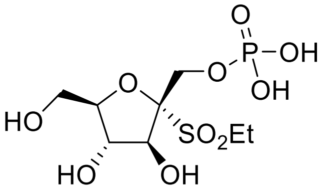

O
P
OH
OH
O
O
HO
SO2Et
HO
OH

1aα

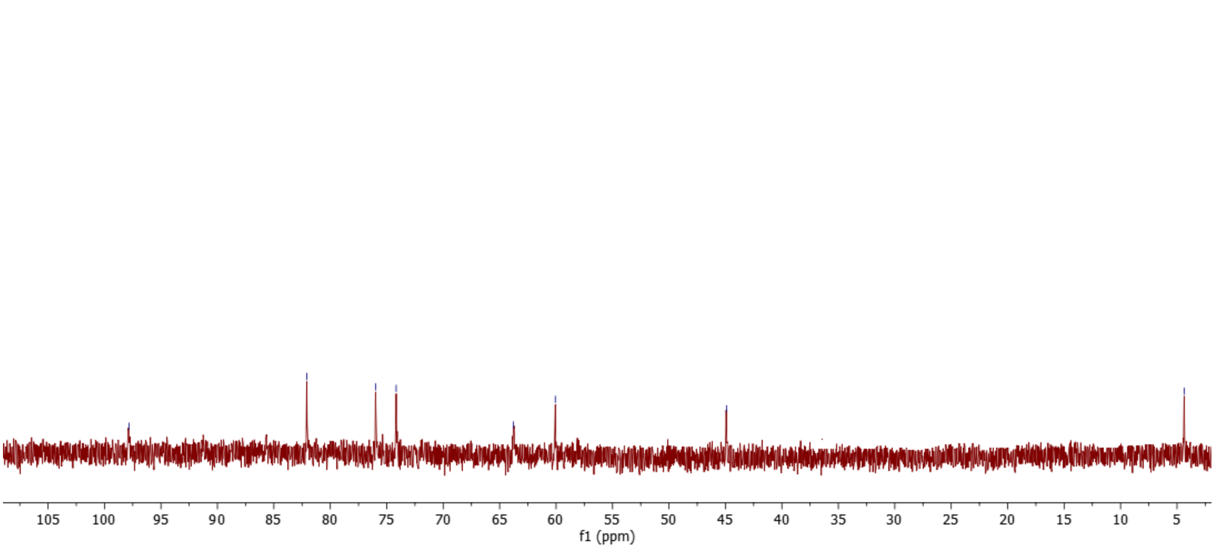

97.82
82.09
75.99
74.17
63.79
60.07
44.91
4.37
105
100
95
90
85
80
75
70
65
60
55
50
45
40
35
30
25
20
15
10
5
f1 (ppm)

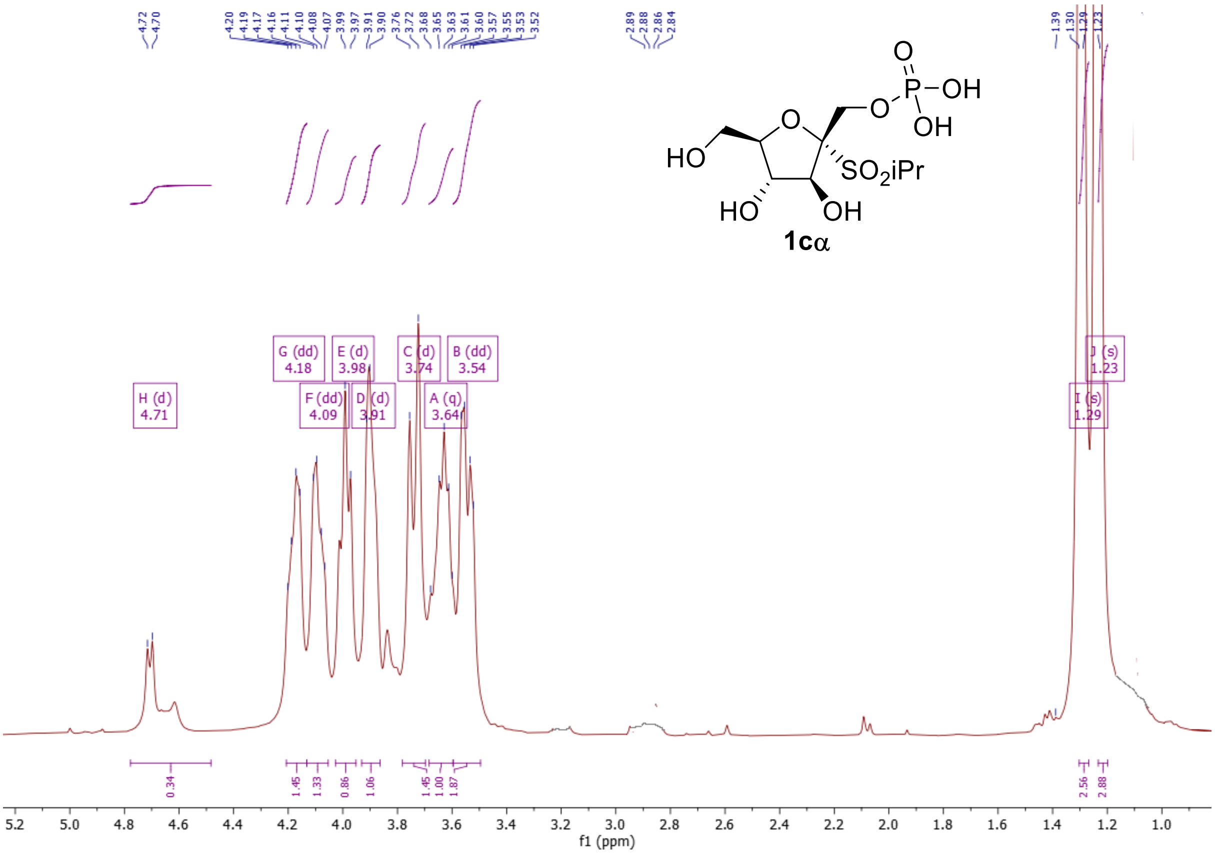

4.72
4.70
4.20
4.19
4.17
4.16
4.11
4.10
4.08
4.07
3.99
3.97
3.91
3.90
3.76
3.72
3.68
3.65
3.63
3.61
3.60
3.57
3.55
3.53
3.52
2.89
2.88
2.86
2.84
1.39
1.30
1.29
1.23
O
P
OH
OH
O
HO
SO2iPr
HO
OH
1cα
G (dd) 4.18
E (d) 3.98
C (d) 3.74
B (dd) 3.54
J (s) 1.23
H (d) 4.71
F (dd) 4.09
D (d) 3.91
A (q) 3.64
I (s) 1.29
0.34
1.45
1.33
0.86
1.06
1.45
1.00
1.87
2.56
2.88
f1 (ppm)

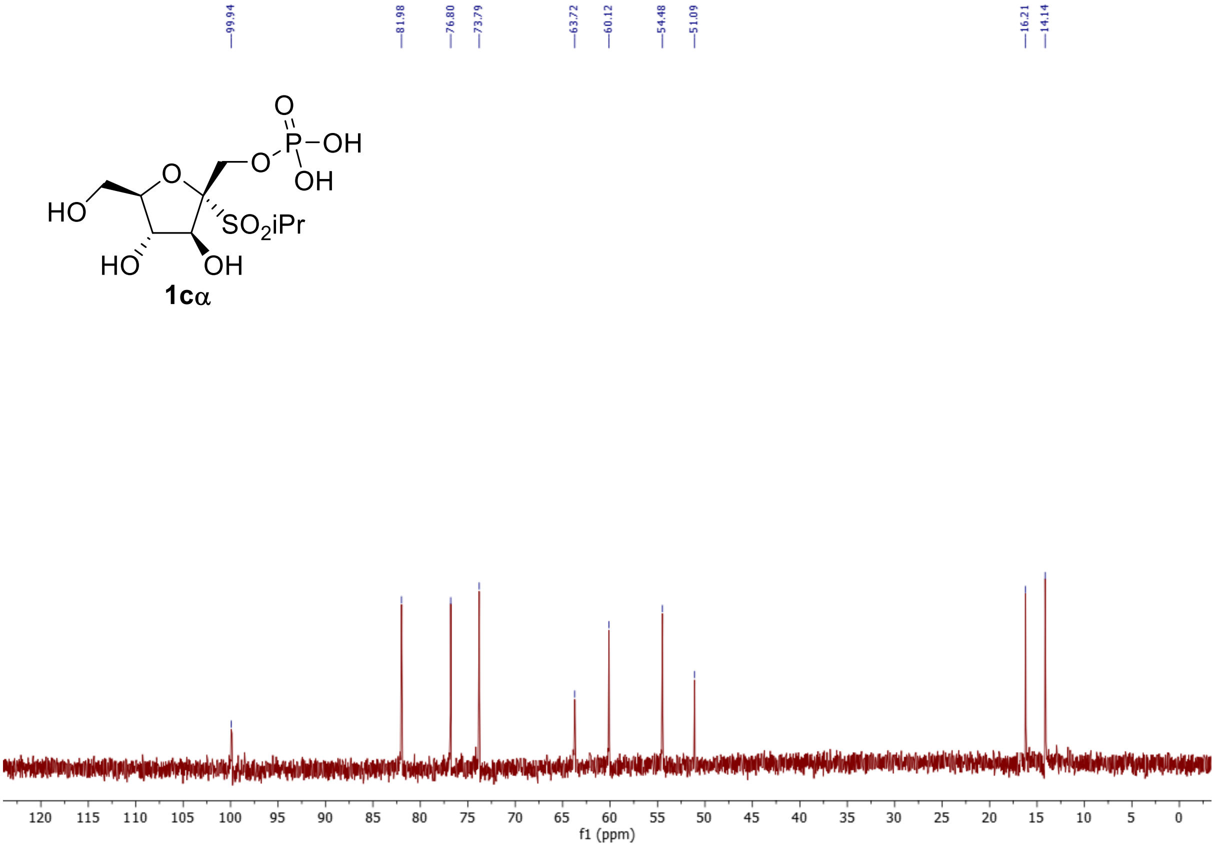

99.94
81.98
76.80
73.79
63.72
60.12
54.48
51.09
16.21
14.14
O
P
OH
OH
O
HO
SO2iPr
HO
OH
1cα
f1 (ppm)

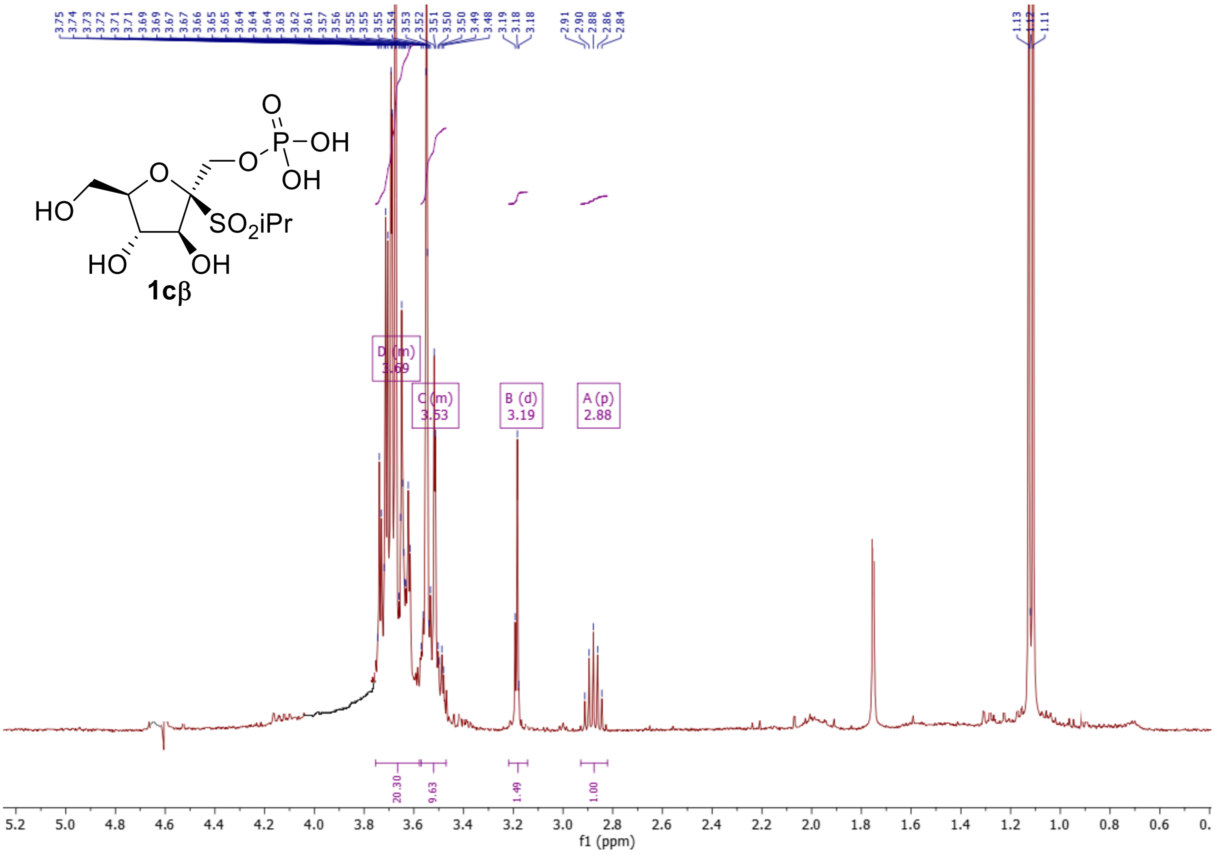
1cβ
D (m) 3.69
C (m) 3.53
B (d) 3.19
A (p) 2.88
20.30
9.63
1.49
1.00
f1 (ppm)

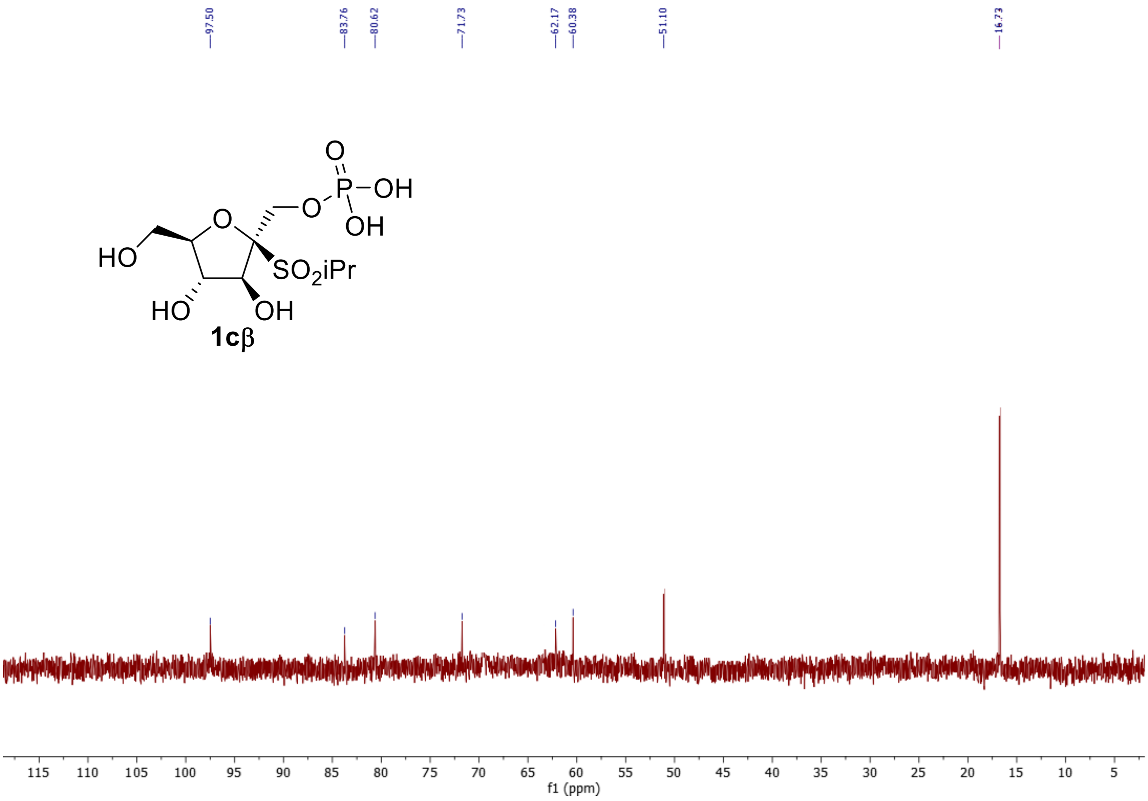
1cβ
97.50
83.76
80.62
71.73
62.17
60.38
51.10
16.73
f1 (ppm)

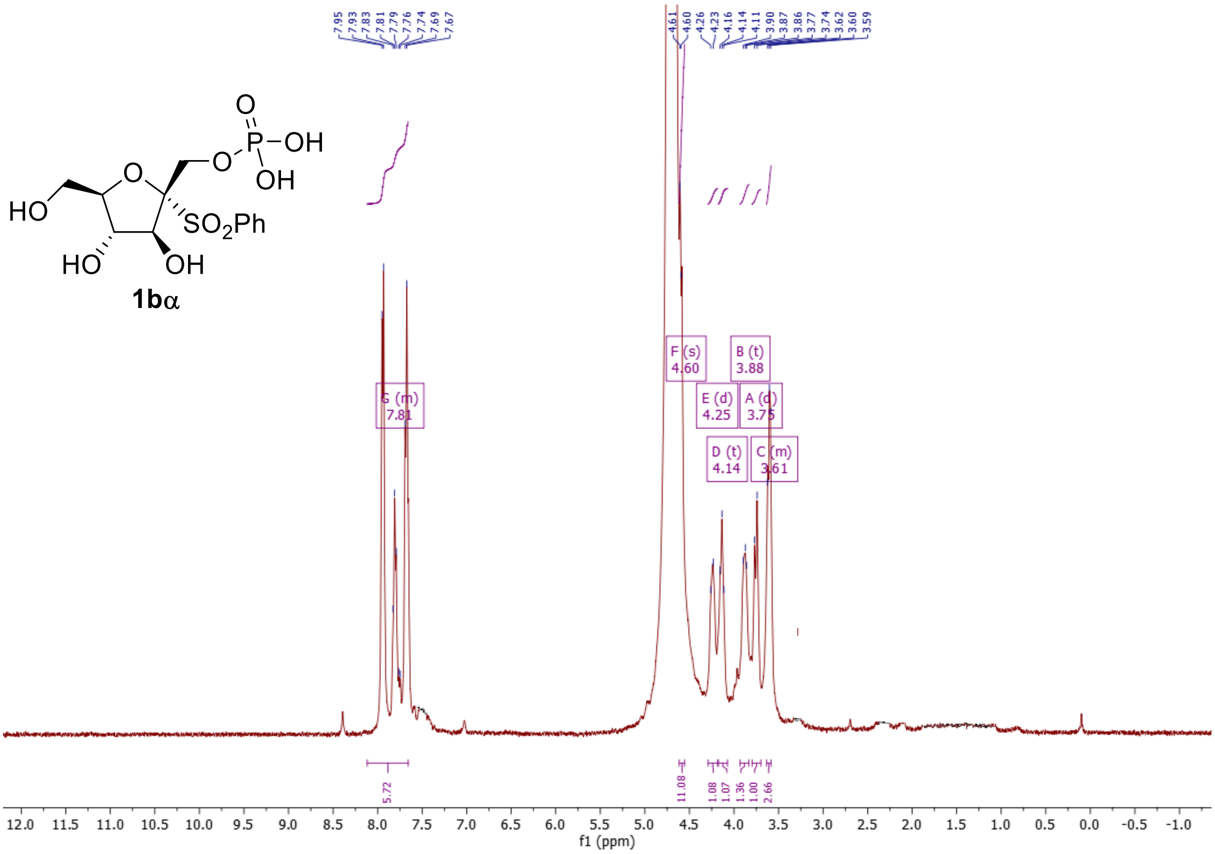
7.95 7.93 7.83 7.81 7.79 7.76 7.74 7.69 7.67
4.61 4.60 4.26 4.23 4.16 4.14 4.11 3.90 3.87 3.86 3.77 3.74 3.62 3.60 3.59
HO
HO
OH
O
O
P
OH
OH
SO2Ph
1bα
G (m) 7.81
F (s) 4.60
E (d) 4.25
D (t) 4.14
B (t) 3.88
A (d) 3.75
C (m) 3.61
5.72
11.08
1.08 1.07 1.36 1.00 2.66
f1 (ppm)

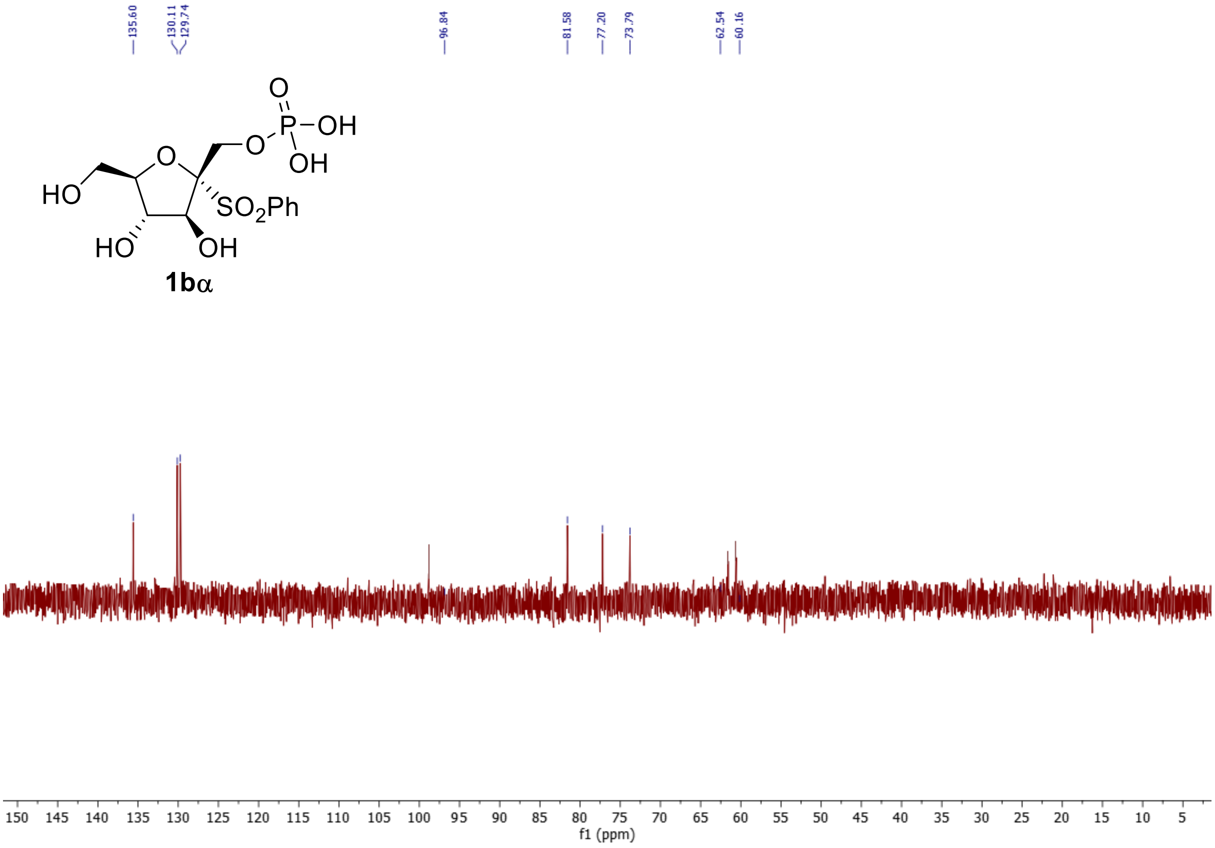
135.60
130.11 129.74
96.84
81.58
77.20
73.79
62.54
60.16
1bα
f1 (ppm)

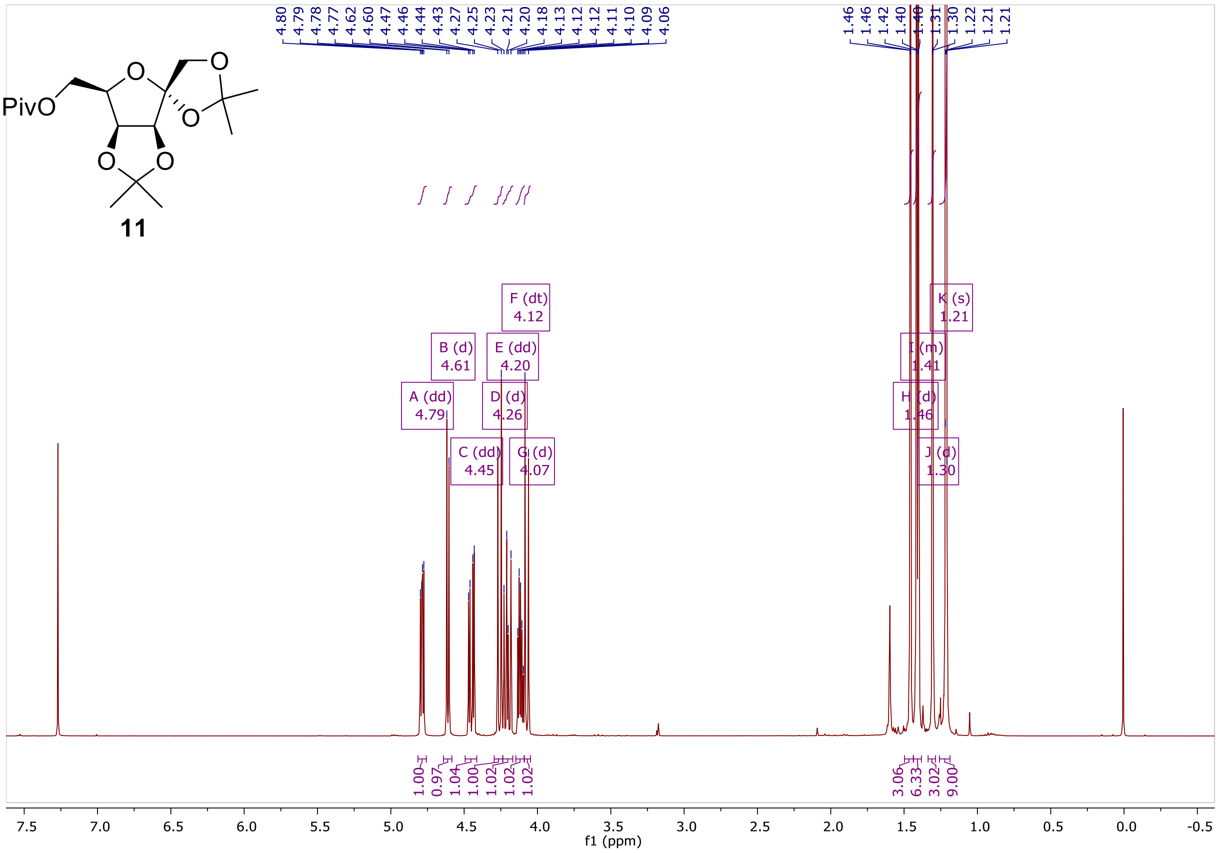
4.80 4.79 4.78 4.77 4.62 4.60 4.47 4.46 4.44 4.43 4.27 4.25 4.23 4.21 4.20 4.18 4.13 4.12 4.12 4.11 4.10 4.09 4.06
1.46 1.46 1.42 1.40 1.40 1.31 1.30 1.22 1.21 1.21
PivO
11
A (dd) 4.79
B (d) 4.61
C (dd) 4.45
D (d) 4.26
E (dd) 4.20
F (dt) 4.12
G (d) 4.07
H (d) 1.46
I (m) 1.41
J (d) 1.30
K (s) 1.21
1.00 0.97 1.04 1.00 1.02 1.02 1.02
3.06 6.33 3.02 9.00
f1 (ppm)

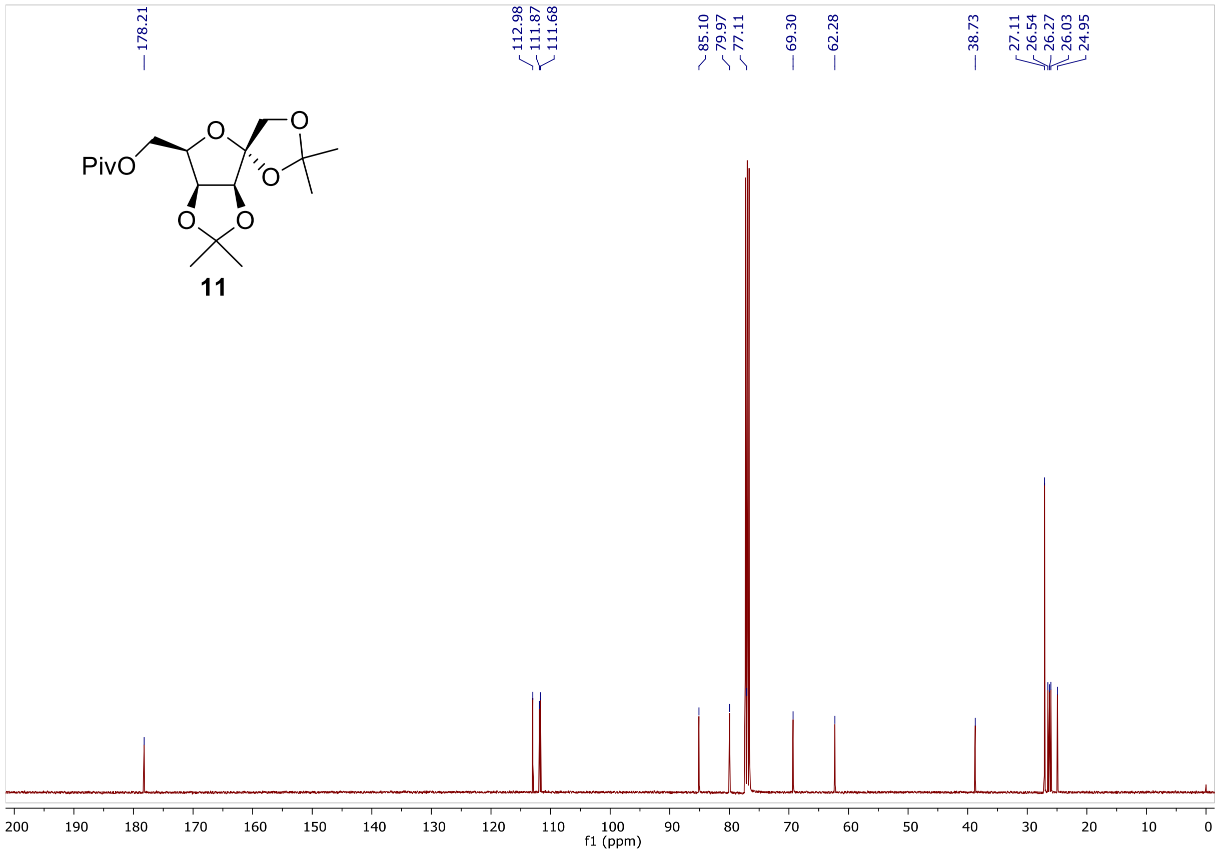
178.21
112.98 111.87 111.68
85.10 79.97 77.11
69.30
62.28
38.73
27.11 26.54 26.27 26.03 24.95
PivO
11
f1 (ppm)

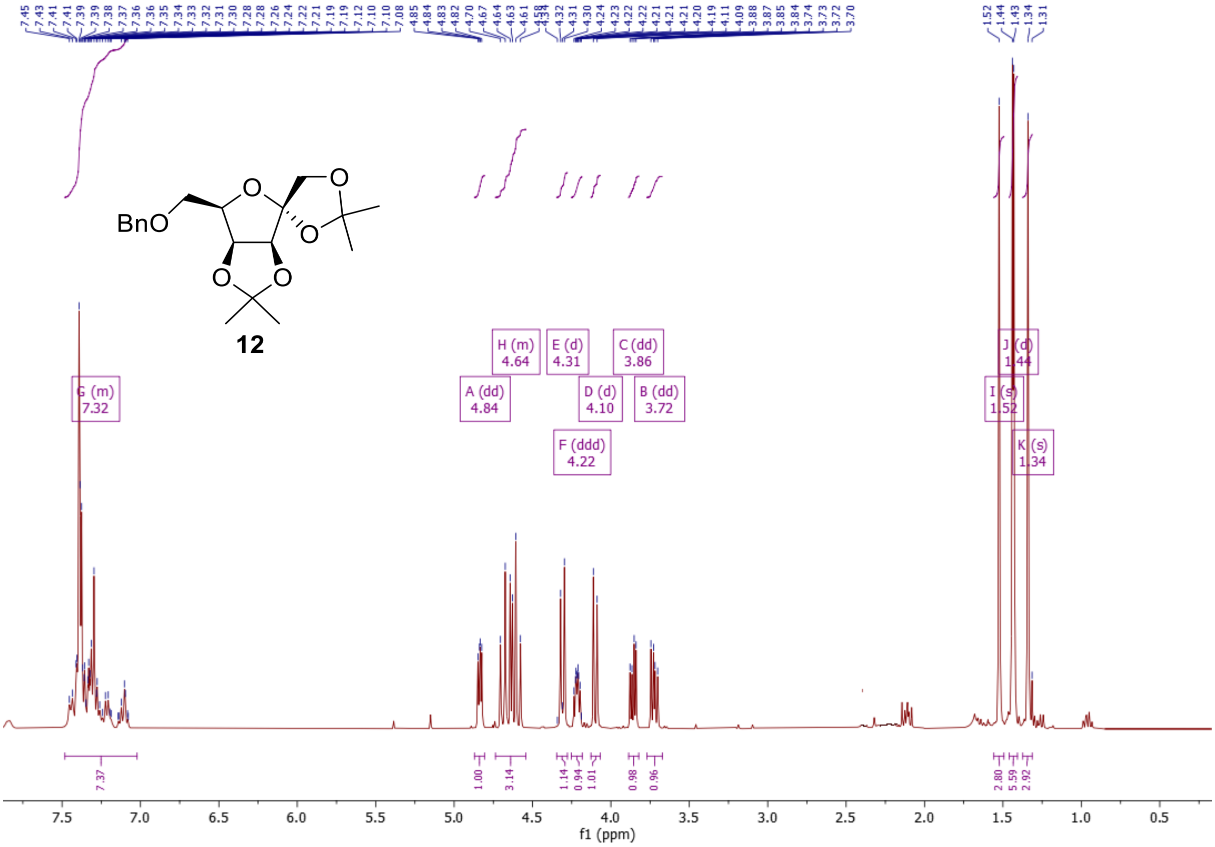

12

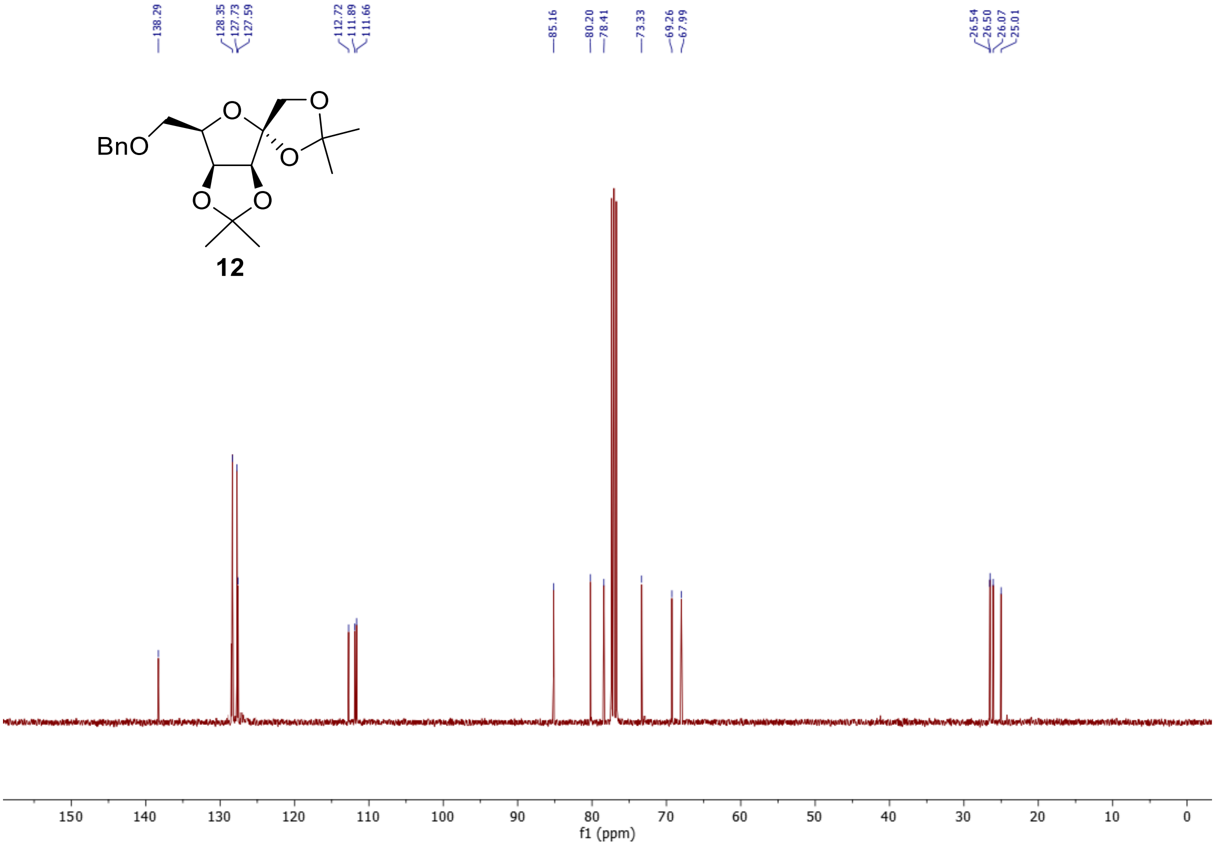

12

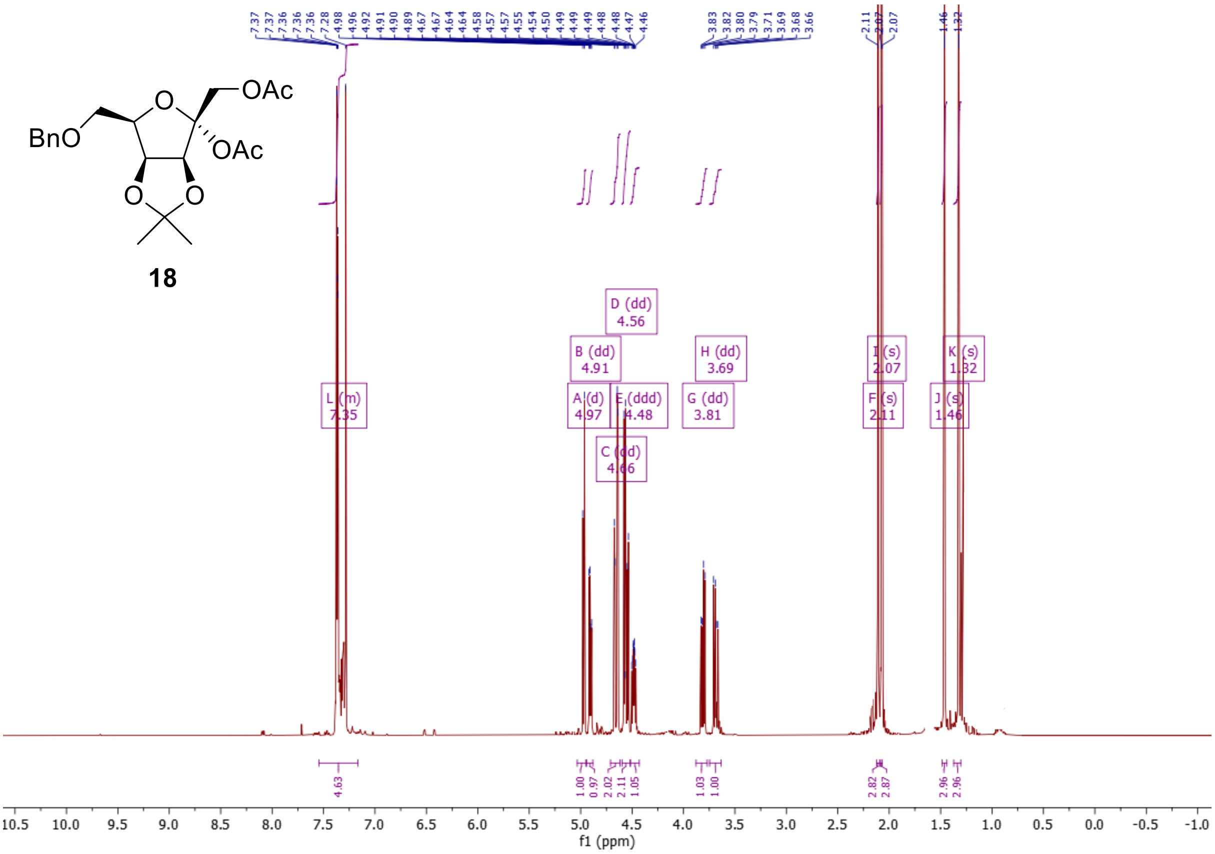
OAc
BnO
O
OAc
O
O
18
7.37
7.37
7.36
7.36
7.36
7.28
4.98
4.96
4.92
4.91
4.90
4.89
4.67
4.64
4.64
4.58
4.57
4.57
4.55
4.54
4.50
4.49
4.49
4.49
4.48
4.47
4.46
3.83
3.82
3.80
3.79
3.71
3.69
3.68
3.66
2.11
2.07
2.07
1.46
1.32
L (m) 7.35
A (d) 4.97
B (dd) 4.91
C (dd) 4.66
D (dd) 4.56
E (ddd) 4.48
G (dd) 3.81
H (dd) 3.69
F (s) 2.11
I (s) 2.07
J (s) 1.46
K (s) 1.32
4.63
1.00
0.97
2.02
2.11
1.05
1.03
1.00
2.82
2.87
2.96
2.96
10.5 10.0 9.5 9.0 8.5 8.0 7.5 7.0 6.5 6.0 5.5 5.0 4.5 4.0 3.5 3.0 2.5 2.0 1.5 1.0 0.5 0.0 -0.5 -1.0
f1 (ppm)

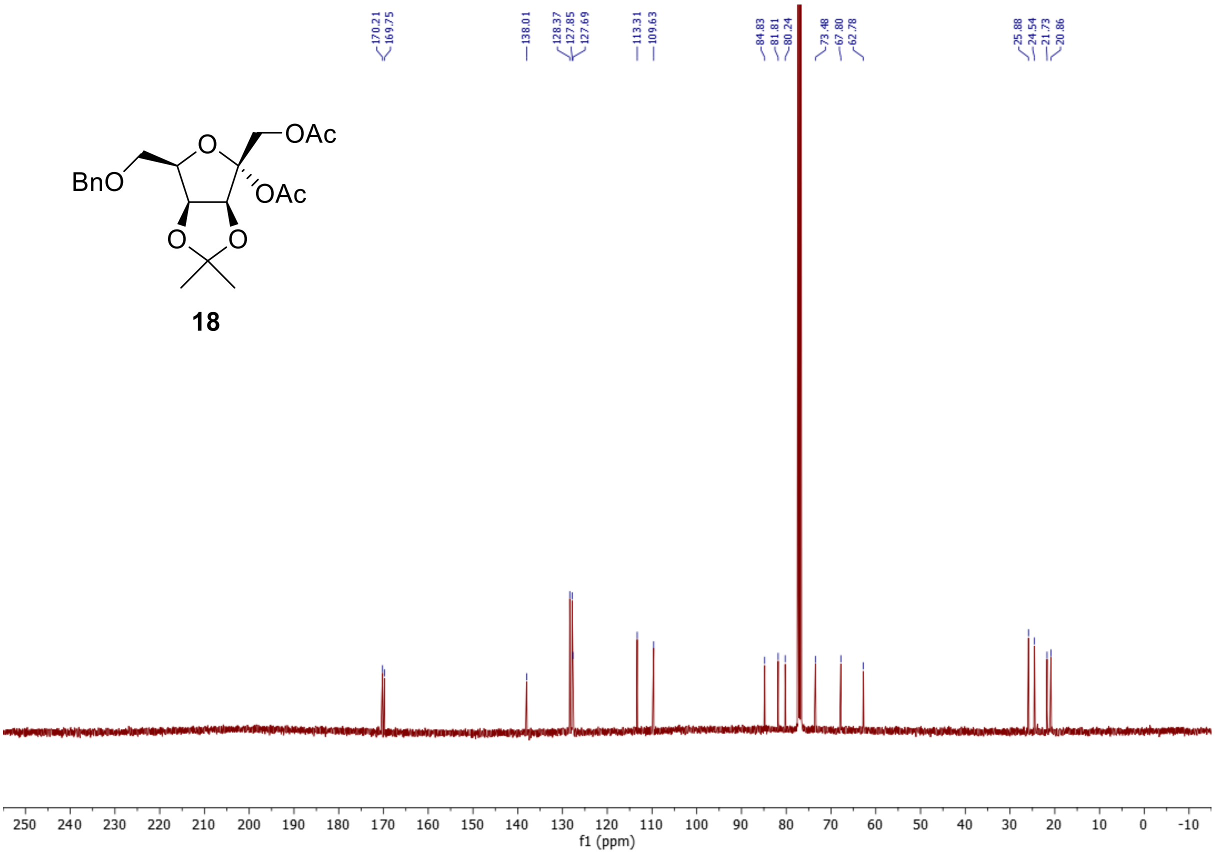
OAc
BnO
O
OAc
O
O
18
170.21
169.75
138.01
128.37
127.85
127.69
113.31
109.63
84.83
81.81
80.24
73.48
67.80
62.78
25.88
24.54
21.73
20.86
250 240 230 220 210 200 190 180 170 160 150 140 130 120 110 100 90 80 70 60 50 40 30 20 10 0 -10
f1 (ppm)

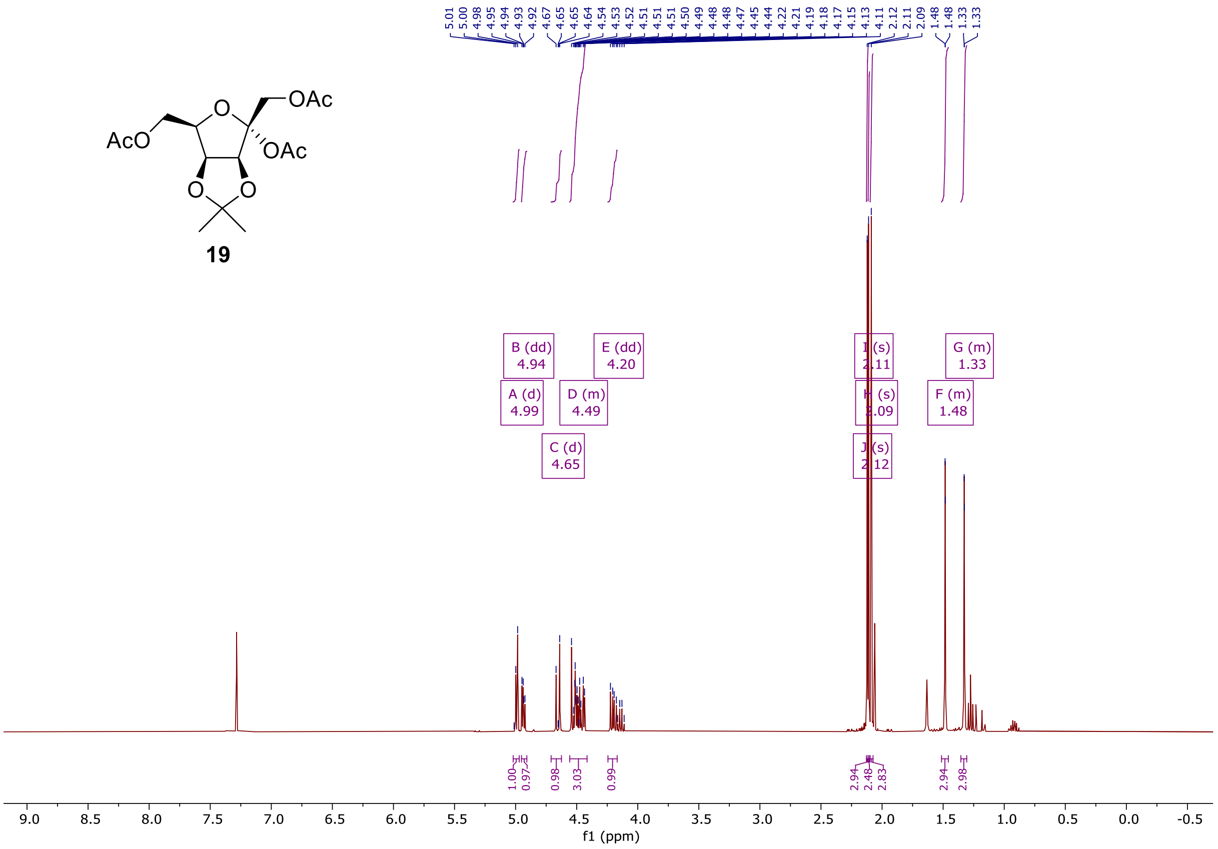
5.01
5.00
4.98
4.95
4.94
4.93
4.92
4.67
4.65
4.65
4.64
4.54
4.53
4.52
4.51
4.51
4.51
4.50
4.49
4.48
4.48
4.47
4.45
4.44
4.22
4.21
4.19
4.18
4.17
4.15
4.13
4.11
2.12
2.11
2.09
1.48
1.48
1.33
1.33
AcO
OAc
OAc
O
O
O
B (dd) 4.94
E (dd) 4.20
A (d) 4.99
D (m) 4.49
C (d) 4.65
I (s) 2.11
H (s) 2.09
J (s) 2.12
G (m) 1.33
F (m) 1.48
1.00
0.97
0.98
3.03
0.99
2.94
2.48
2.83
2.94
2.98
f1 (ppm)

19

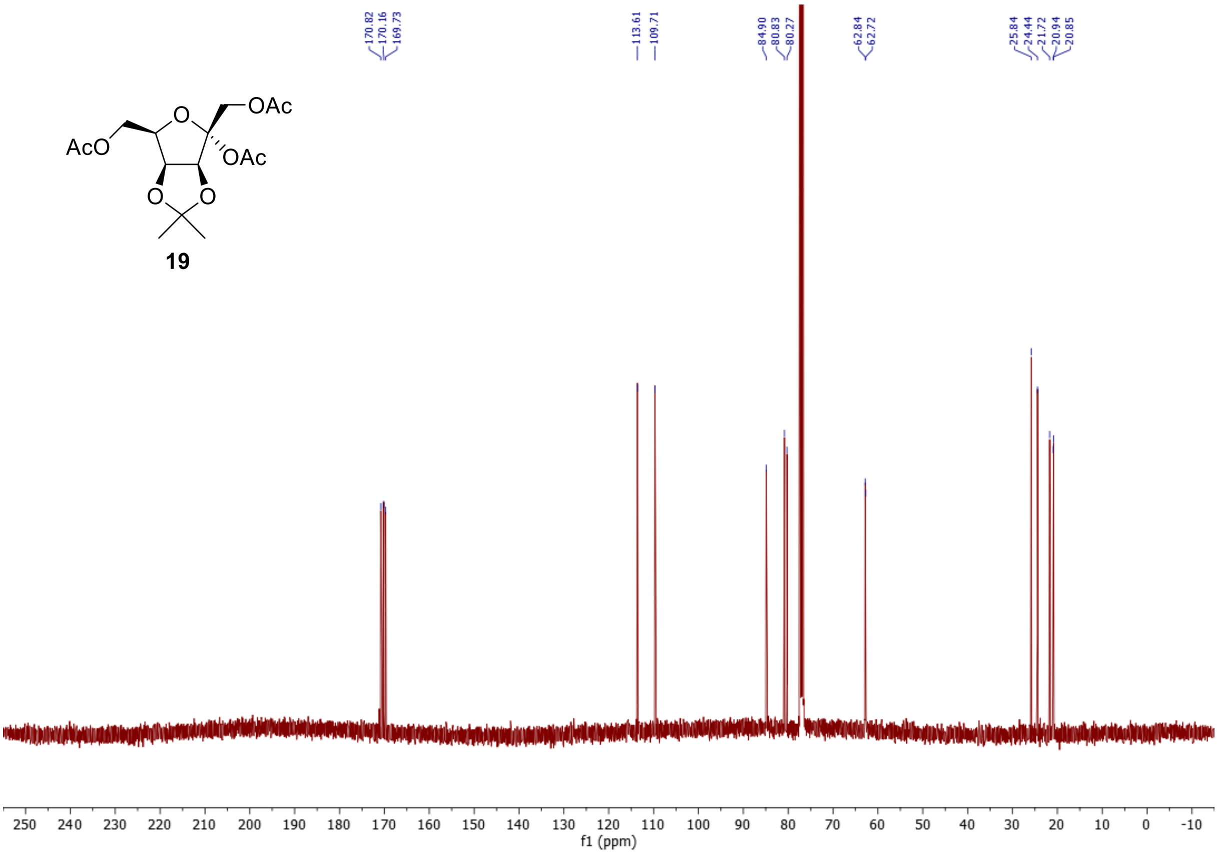
170.82
170.16
169.73
113.61
109.71
84.90
80.83
80.27
62.84
62.72
25.84
24.44
21.72
20.94
20.85
AcO
OAc
OAc
O
O
O
f1 (ppm)

19

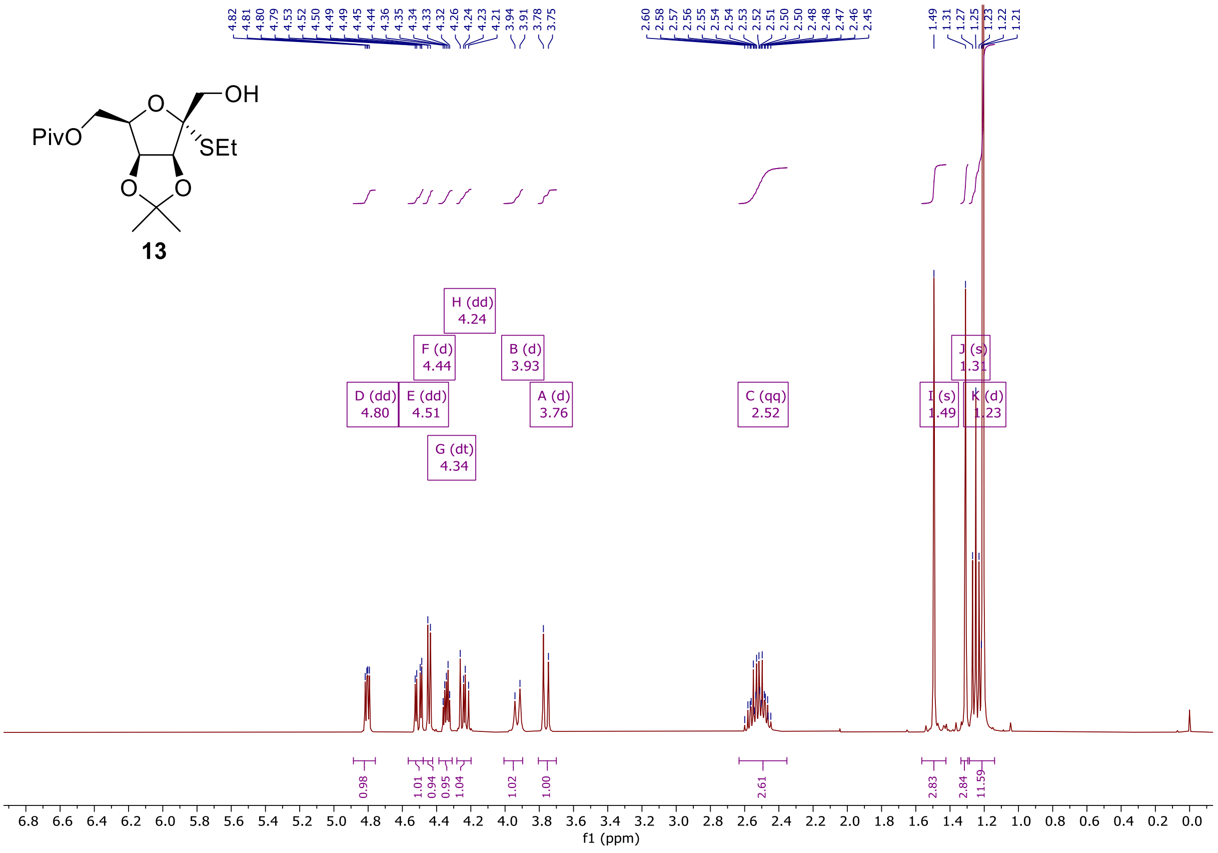

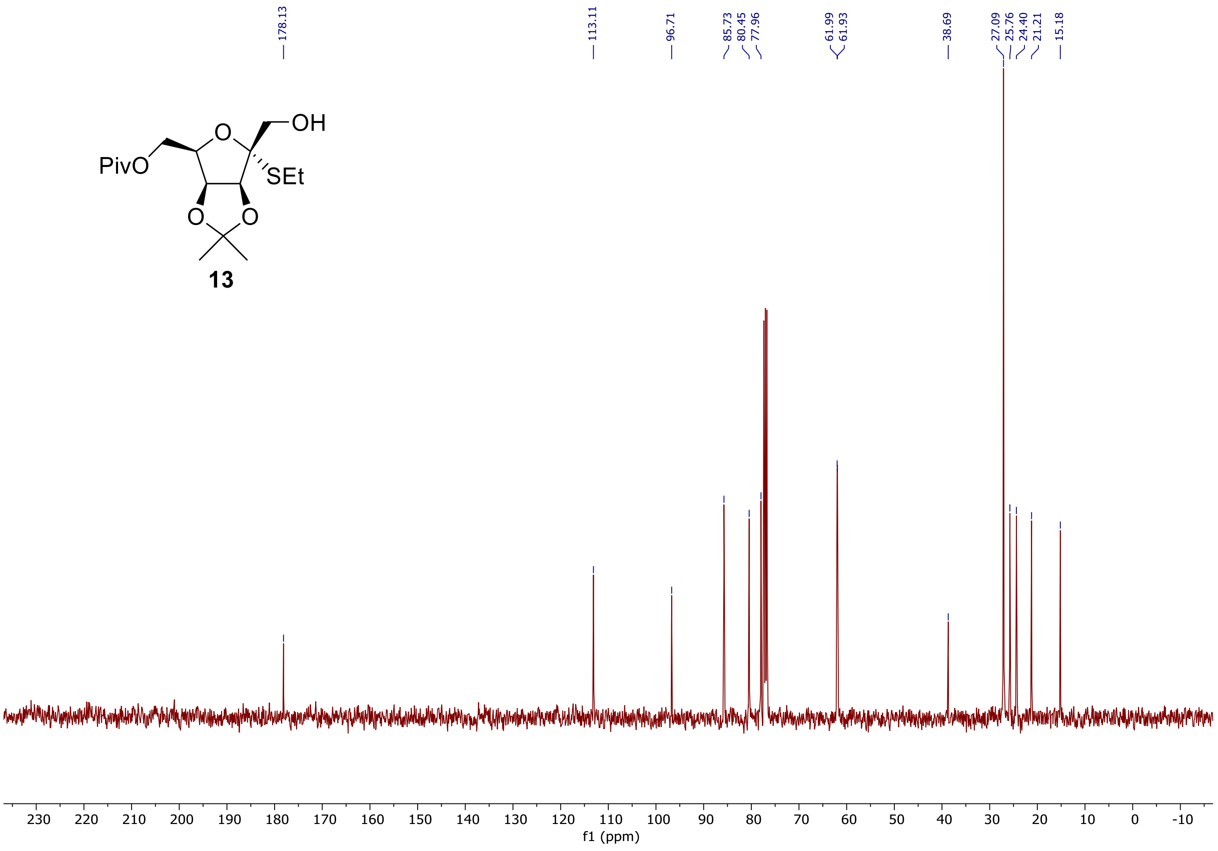

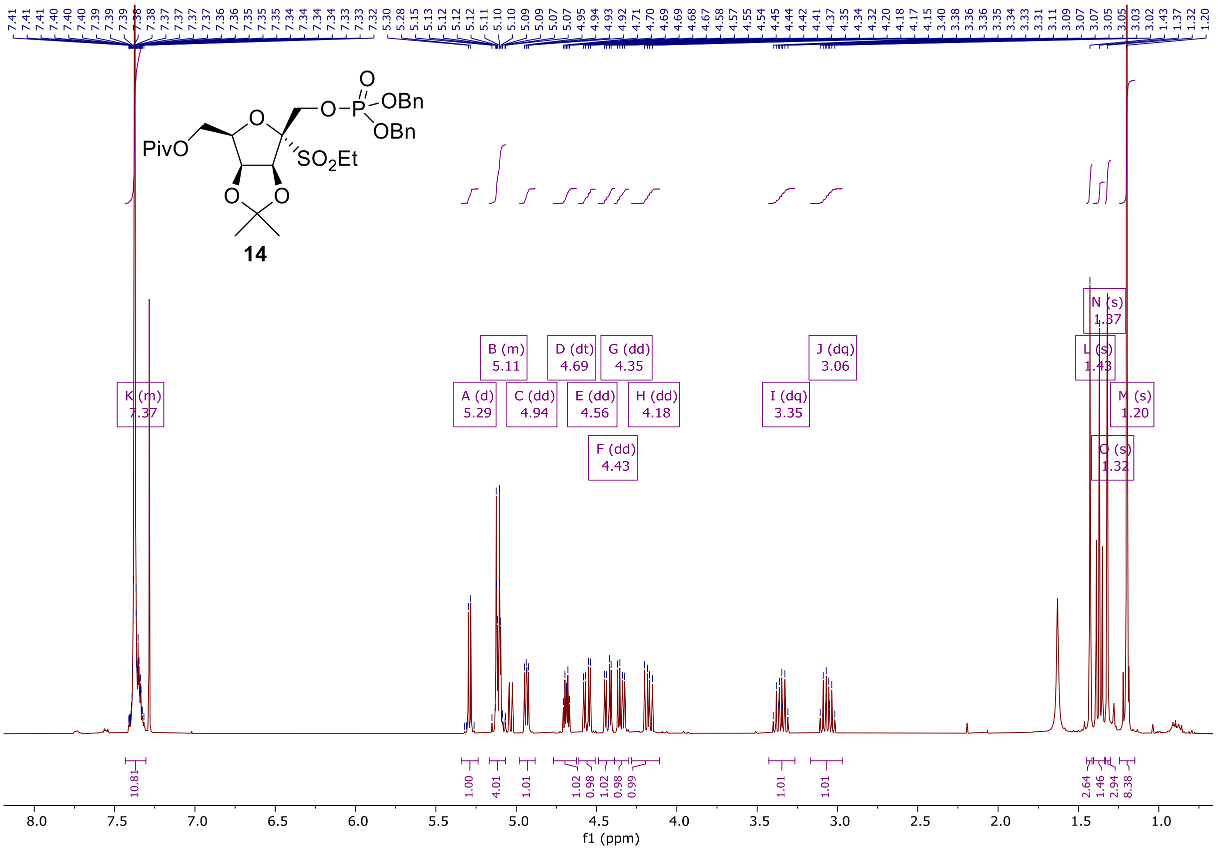

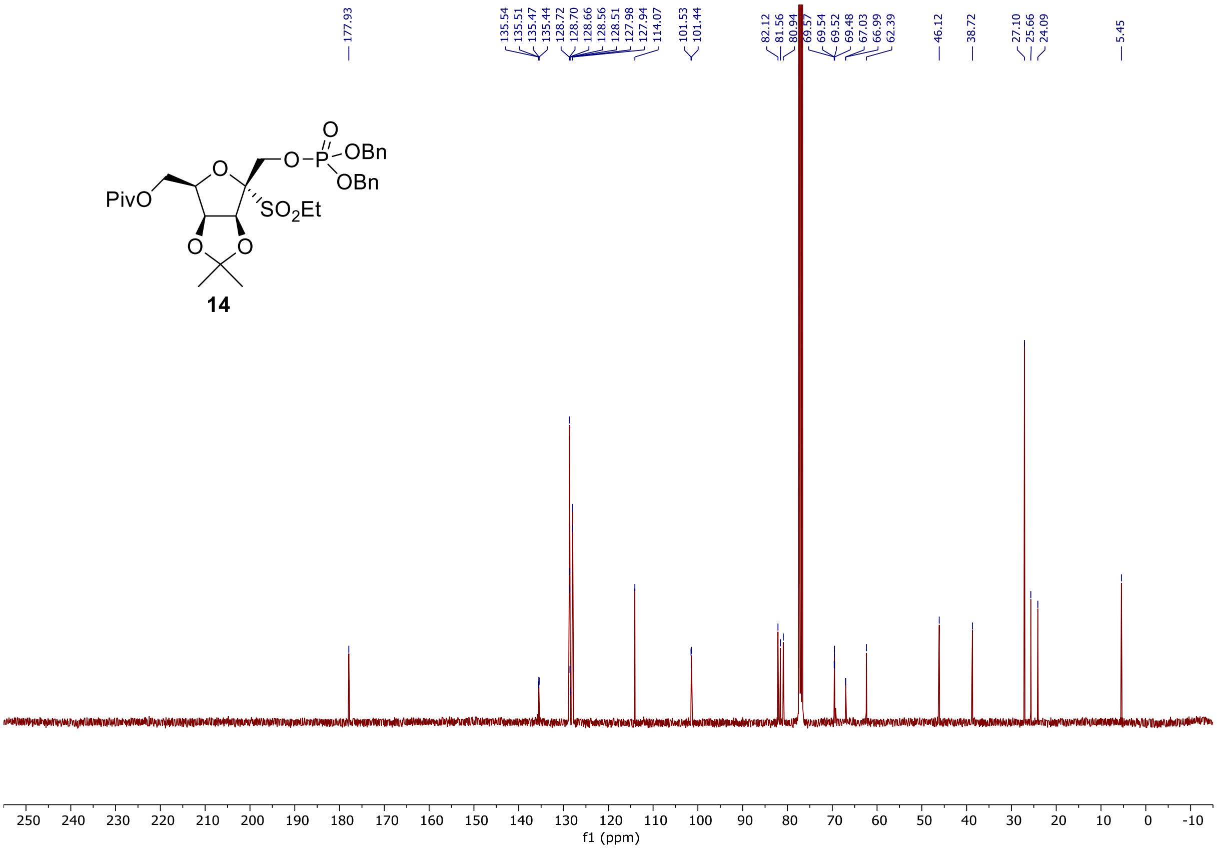

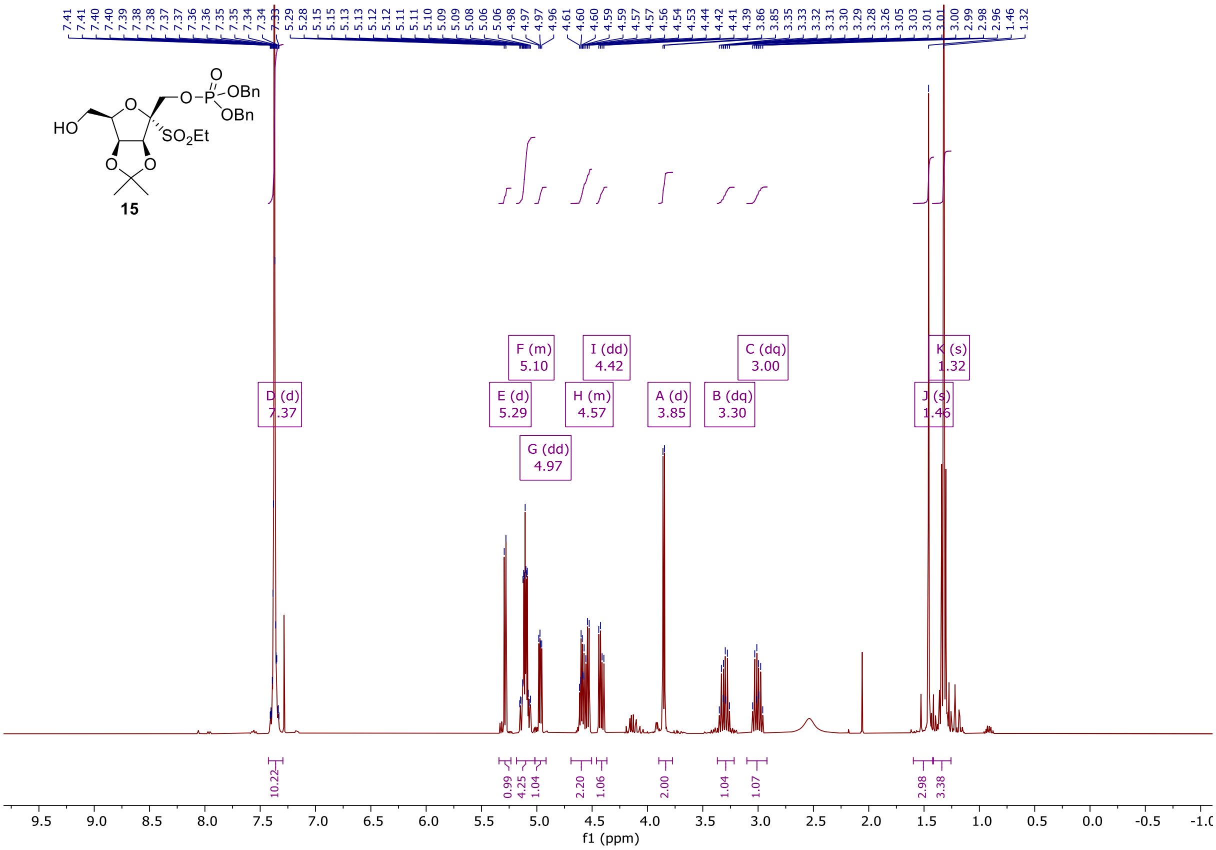

HO
O
O-P-OBn
OBn
SO2Et
15
D (d) 7.37
E (d) 5.29
F (m) 5.10
G (dd) 4.97
H (m) 4.57
I (dd) 4.42
A (d) 3.85
B (dq) 3.30
C (dq) 3.00
J (s) 1.46
K (s) 1.32
f1 (ppm)

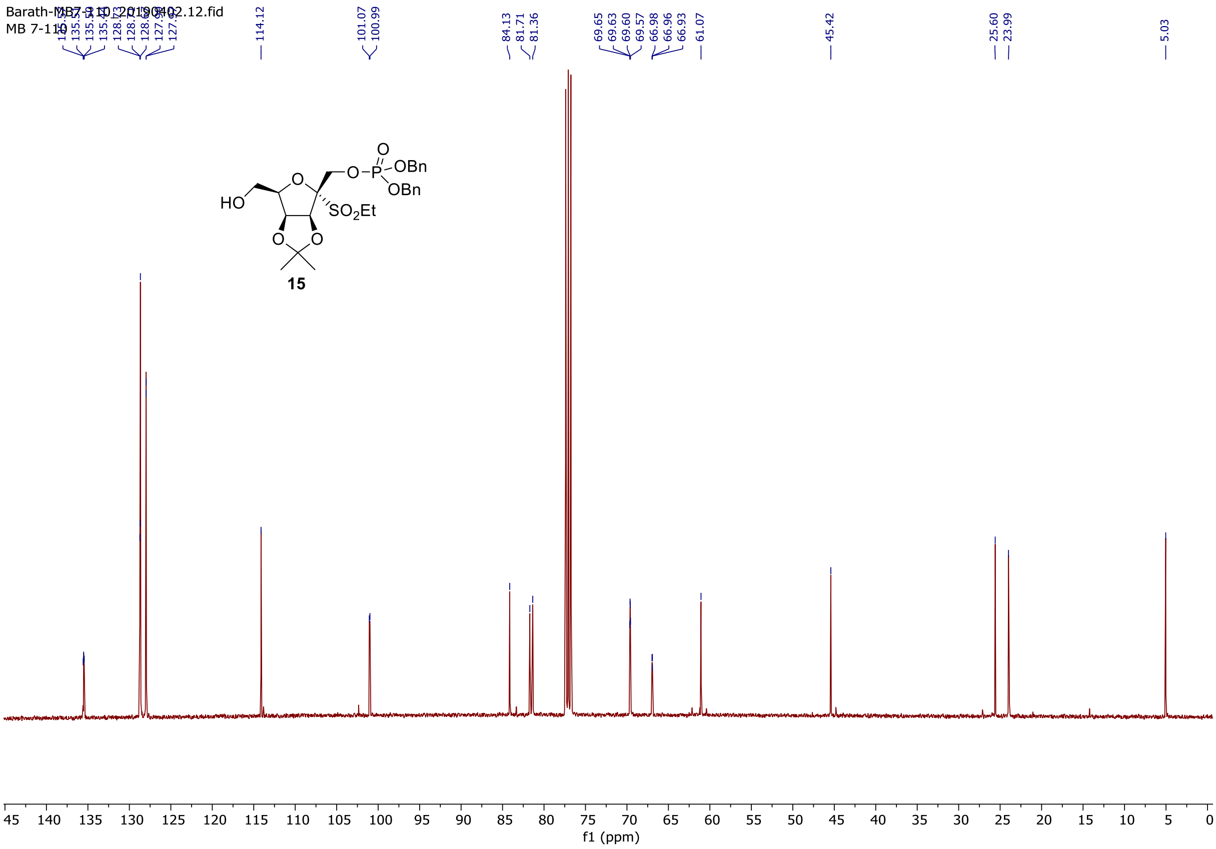

Barath-MB7-110_20190402.12.fid
MB 7-110
114.12
101.07
100.99
84.13
81.71
81.36
69.65
69.63
69.60
69.57
66.98
66.96
66.93
61.07
45.42
25.60
23.99
5.03
HO
O
O-P-OBn
OBn
SO2Et
15
f1 (ppm)

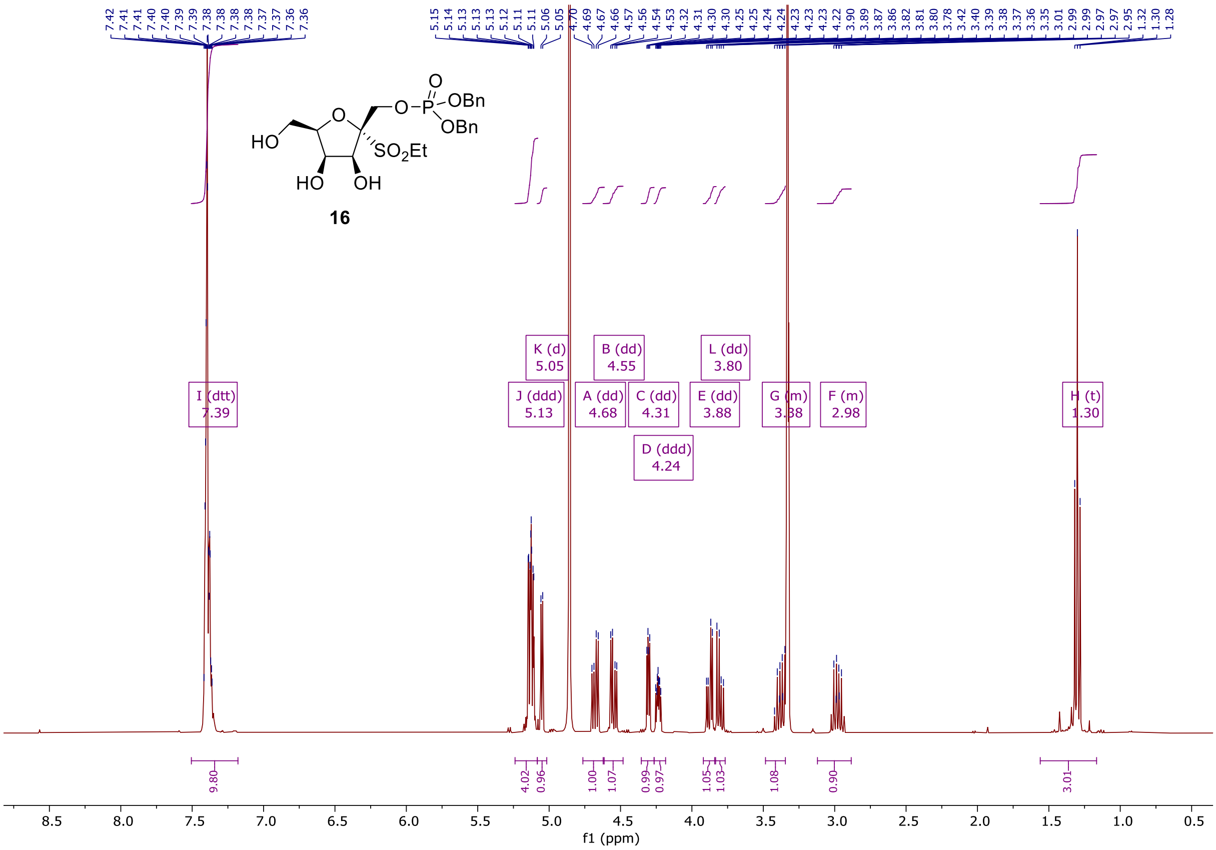

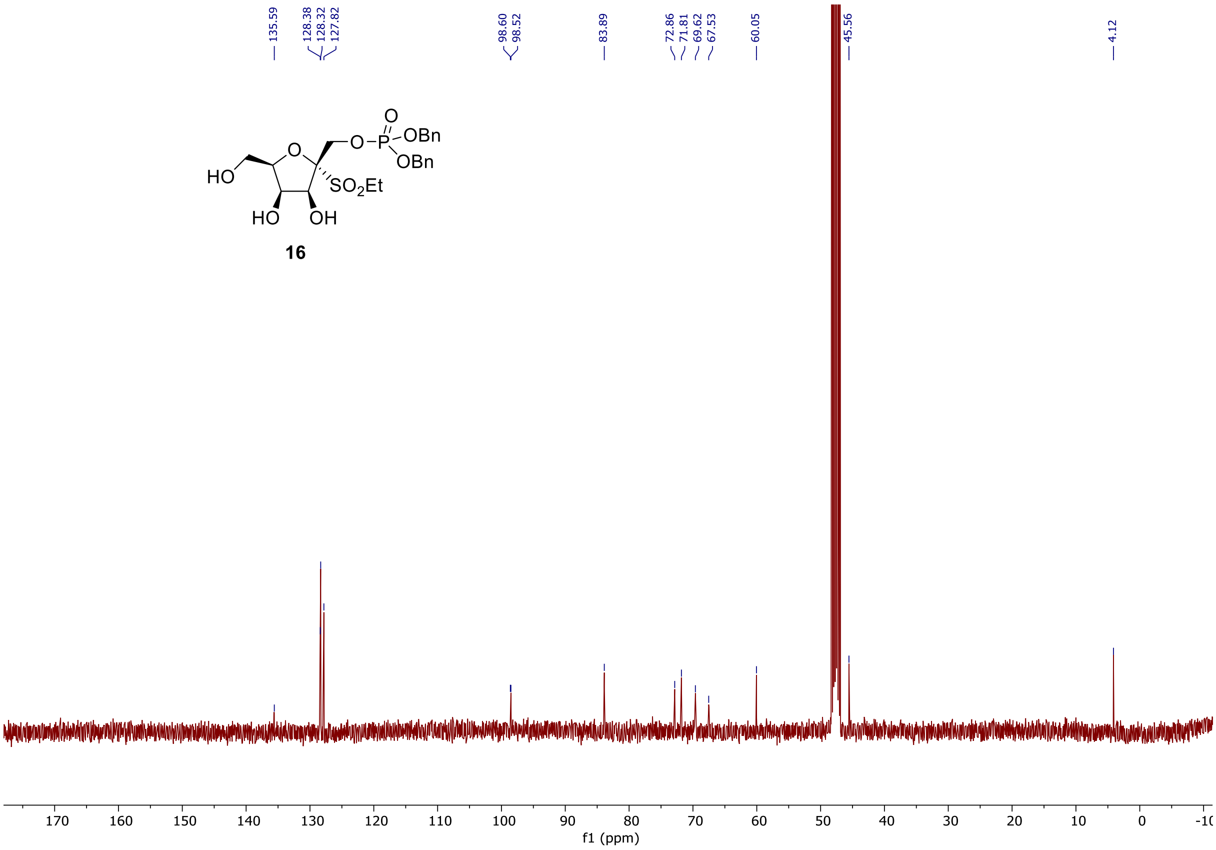

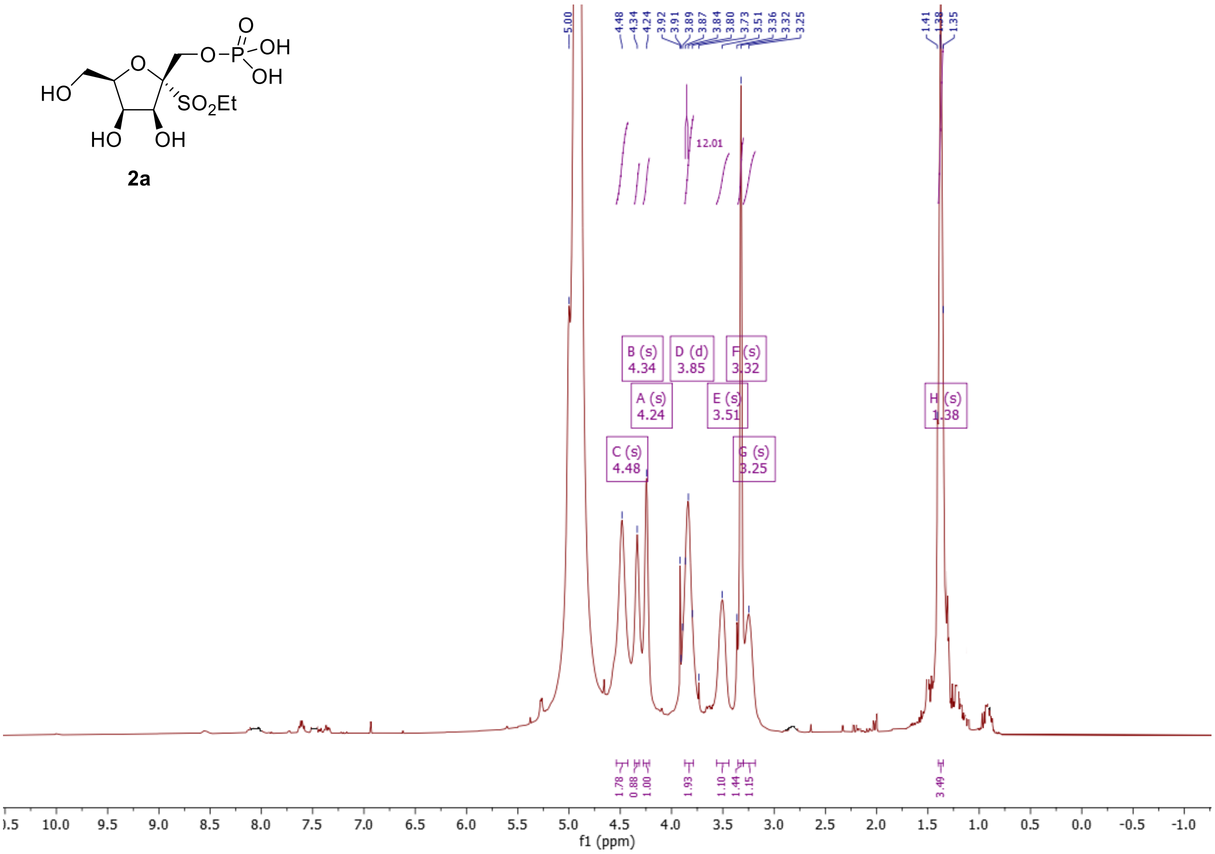
O
OH
O–P
OH
HO
O
SO2Et
HO
OH
2a
5.00
4.48
4.34
4.24
3.92
3.91
3.89
3.87
3.84
3.80
3.73
3.51
3.36
3.32
3.25
1.41
1.38
1.35
12.01
B (s)
4.34
D (d)
3.85
F (s)
3.32
A (s)
4.24
E (s)
3.51
H (s)
1.38
C (s)
4.48
G (s)
3.25
1.78
0.88
1.00
1.93
1.10
1.44
1.15
3.49
f1 (ppm)

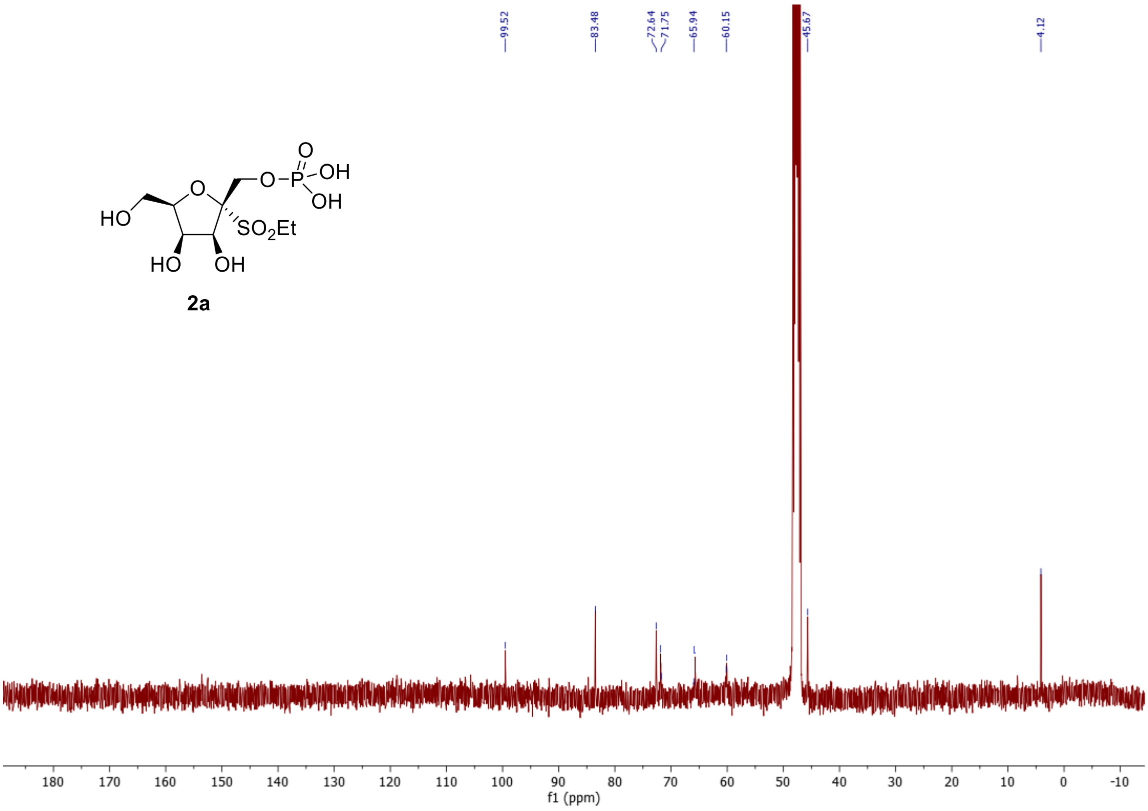
99.52
83.48
72.64
71.75
65.94
60.15
45.67
4.12
O
OH
O–P
OH
HO
O
SO2Et
HO
OH
2a
f1 (ppm)

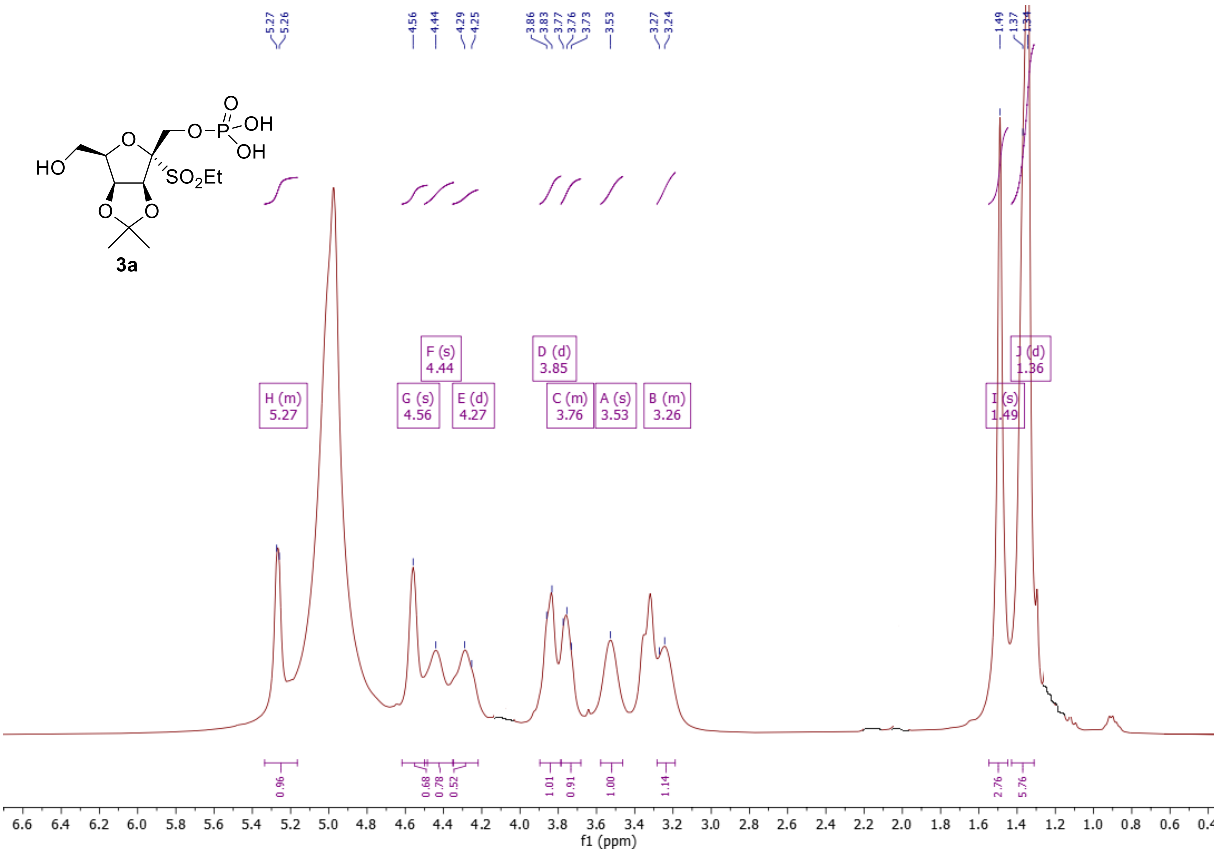
5.27
5.26
4.56
4.44
4.29
4.25
3.86
3.83
3.77
3.76
3.73
3.53
3.27
3.24
1.49
1.37
1.34
HO
O
O-P-OH
OH
SO2Et
3a
H (m) 5.27
G (s) 4.56
F (s) 4.44
E (d) 4.27
D (d) 3.85
C (m) 3.76
A (s) 3.53
B (m) 3.26
I (s) 1.49
J (d) 1.36
0.96
0.68
0.78
0.52
1.01
0.91
1.00
1.14
2.76
5.76
f1 (ppm)

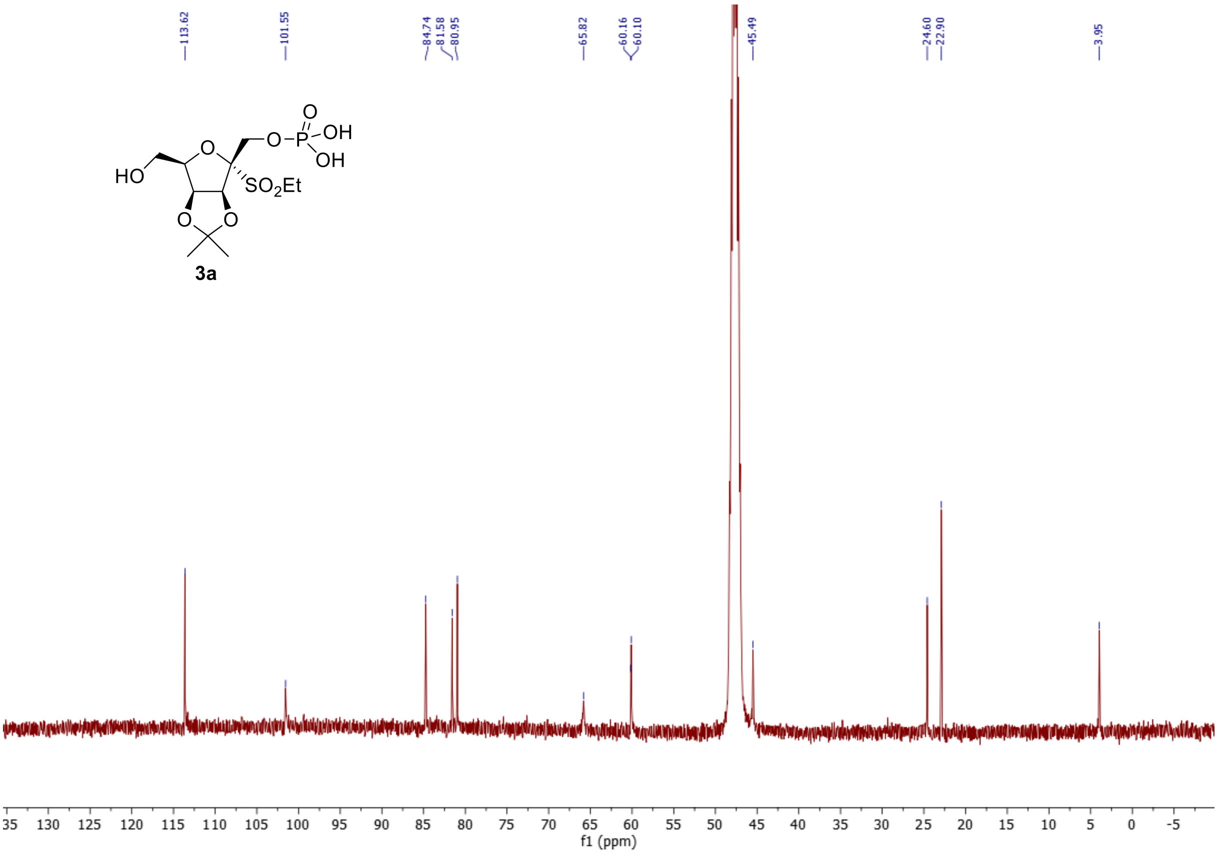
113.62
101.55
84.74
81.58
80.95
65.82
60.16
60.10
45.49
24.60
22.90
3.95
HO
O
O-P-OH
OH
SO2Et
3a
f1 (ppm)

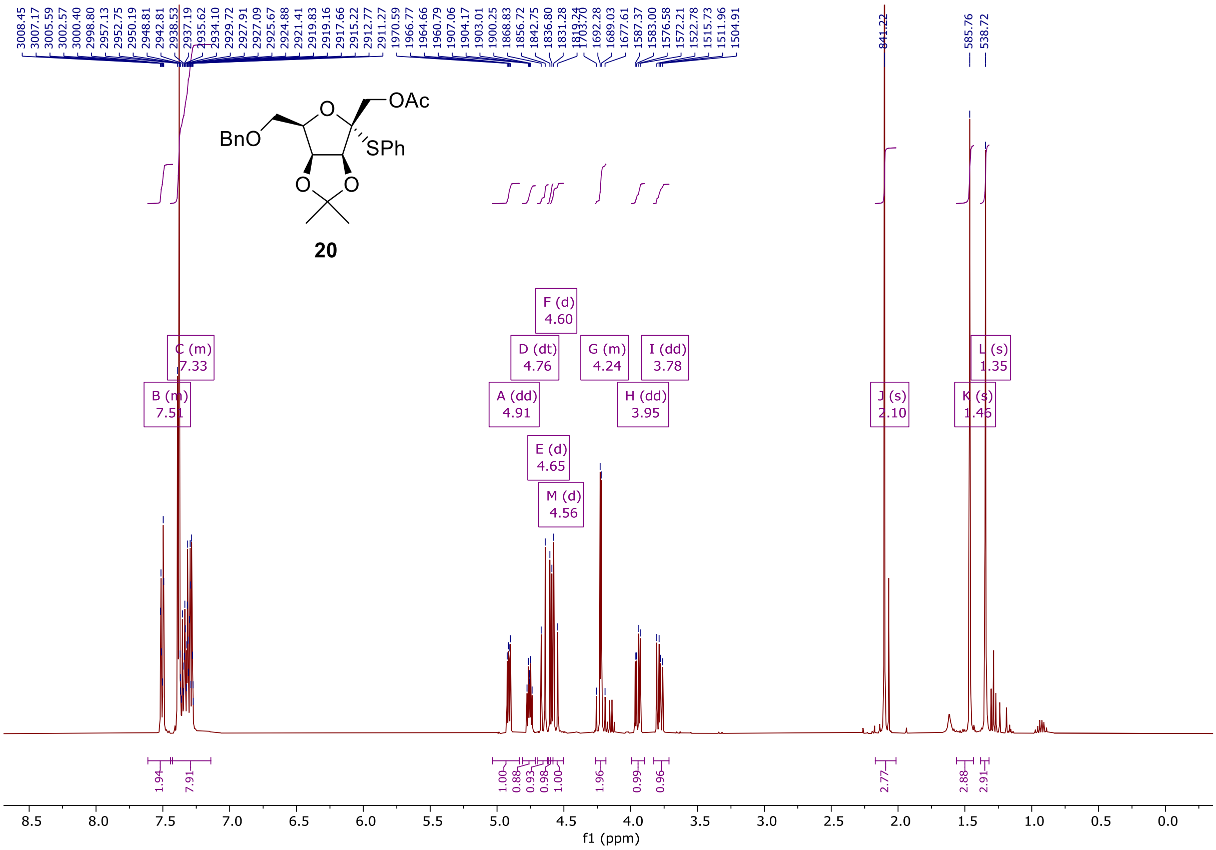
OAc
BnO
SPh
O
20
f1 (ppm)

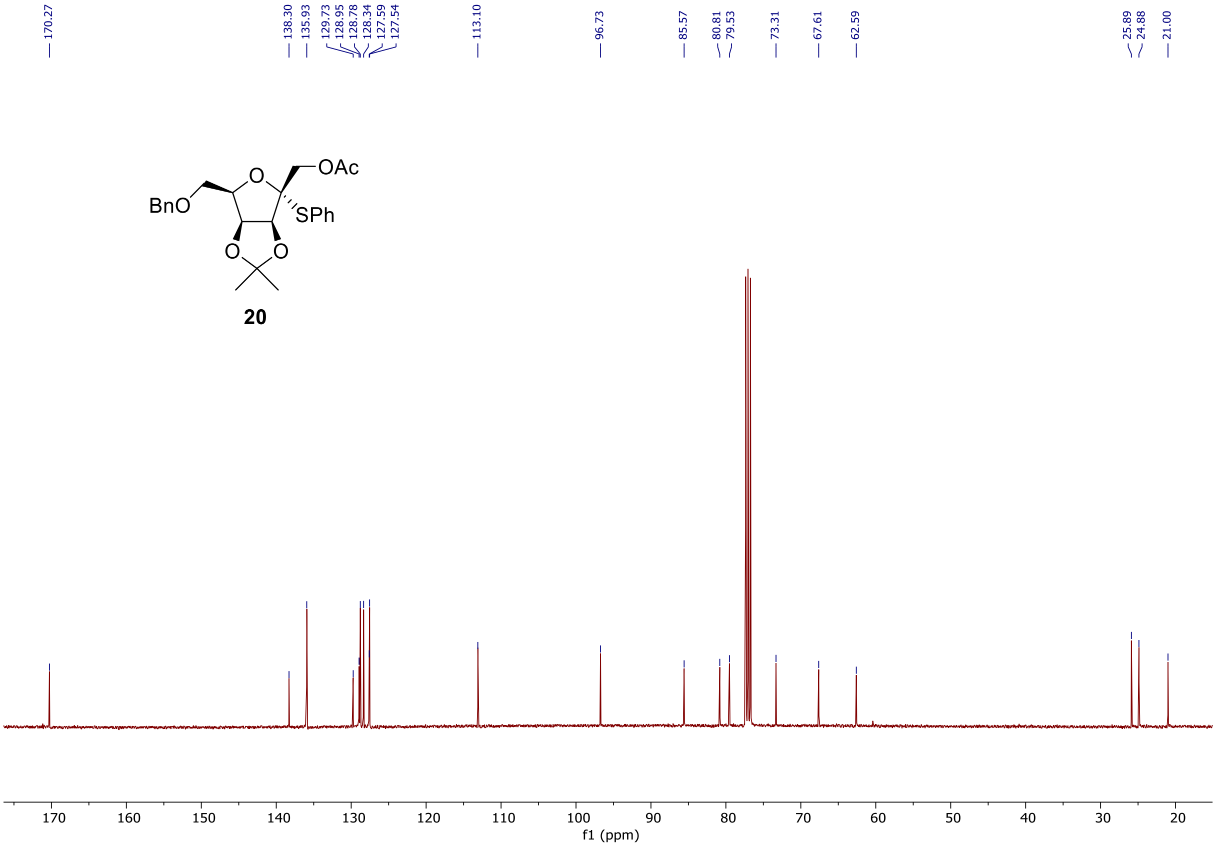
OAc
BnO
SPh
O
20
f1 (ppm)

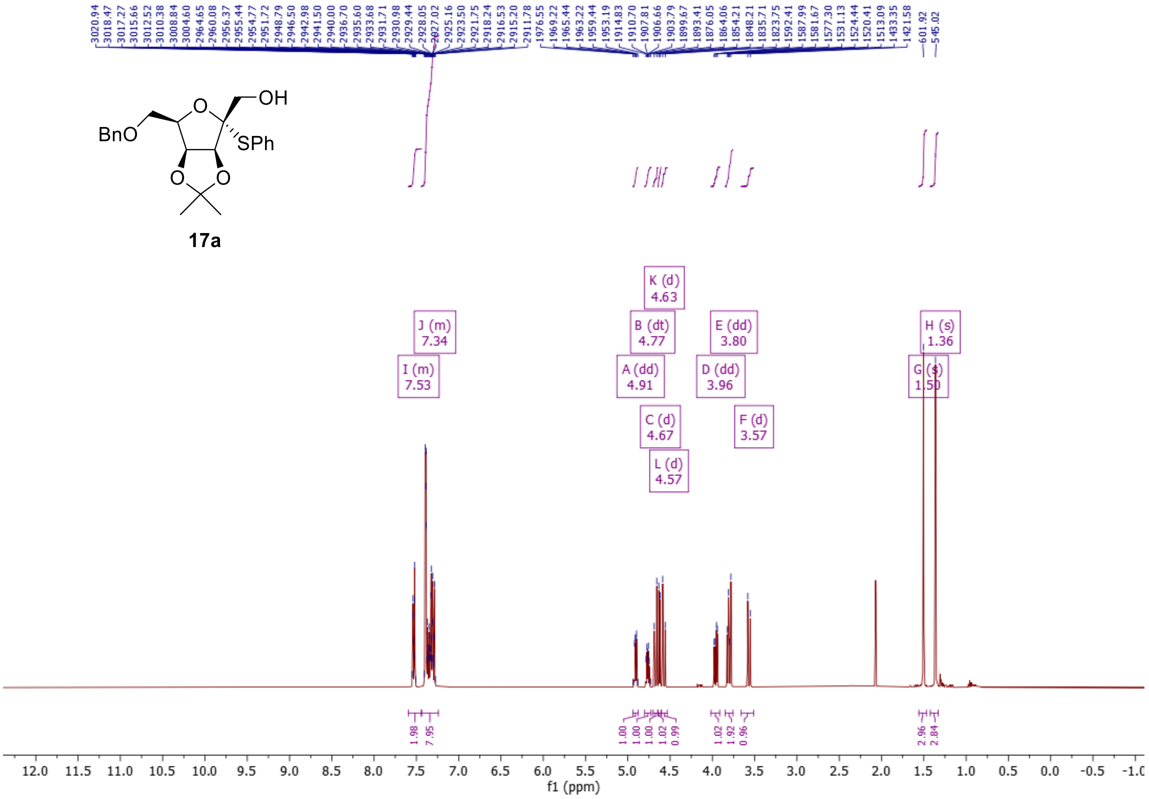
BnO
OH
SPh
17a
I (m) 7.53
J (m) 7.34
A (dd) 4.91
B (dt) 4.77
C (d) 4.67
K (d) 4.63
L (d) 4.57
D (dd) 3.96
E (dd) 3.80
F (d) 3.57
G (s) 1.50
H (s) 1.36
1.98
7.95
1.00
1.00
1.00
1.02
0.99
1.02
1.92
0.96
2.96
2.84
f1 (ppm)

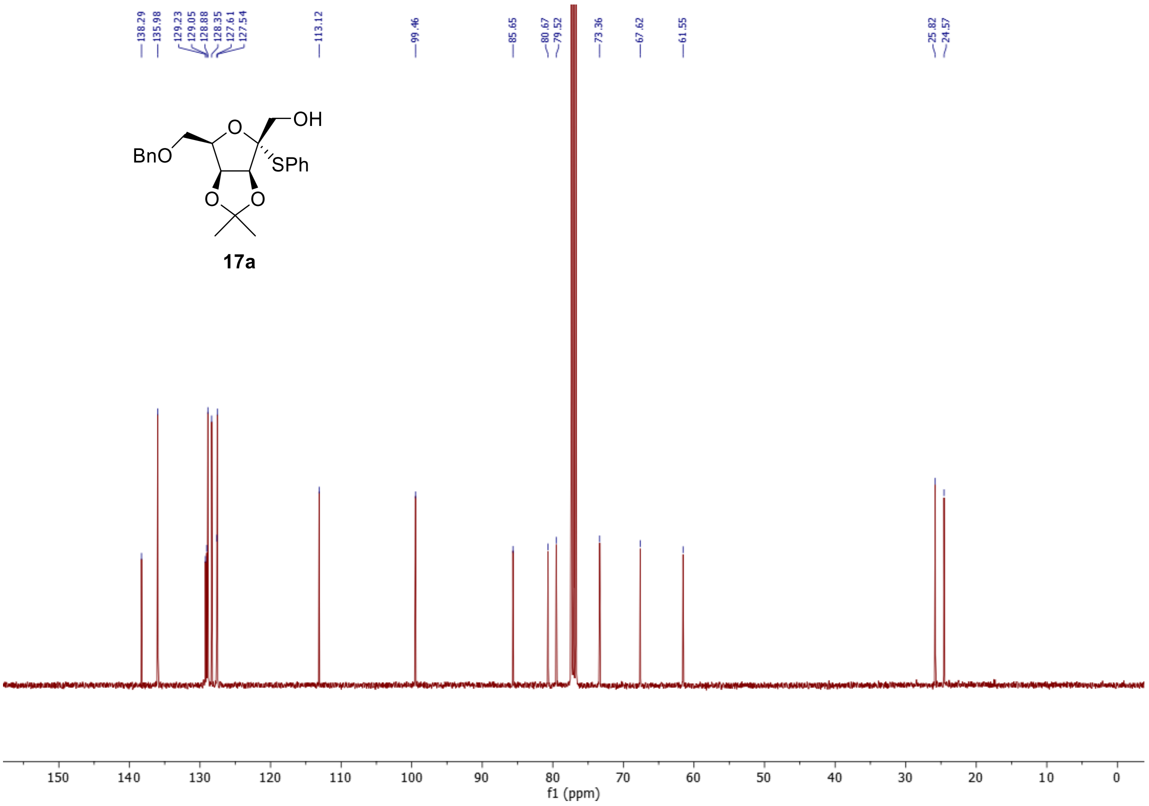
138.29
135.98
129.23
129.05
128.88
128.35
127.61
127.54
113.12
99.46
85.65
80.67
79.52
73.36
67.62
61.55
25.82
24.57
BnO
OH
SPh
17a
f1 (ppm)

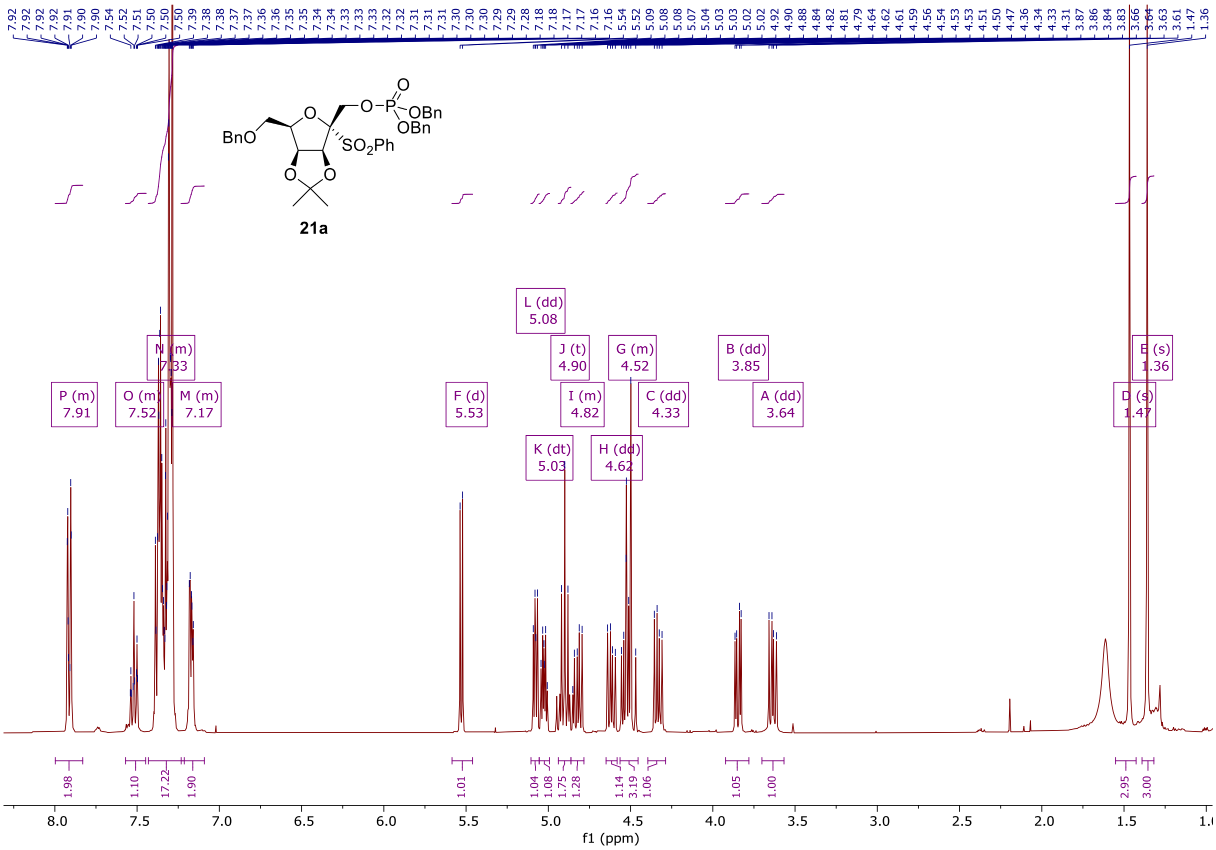

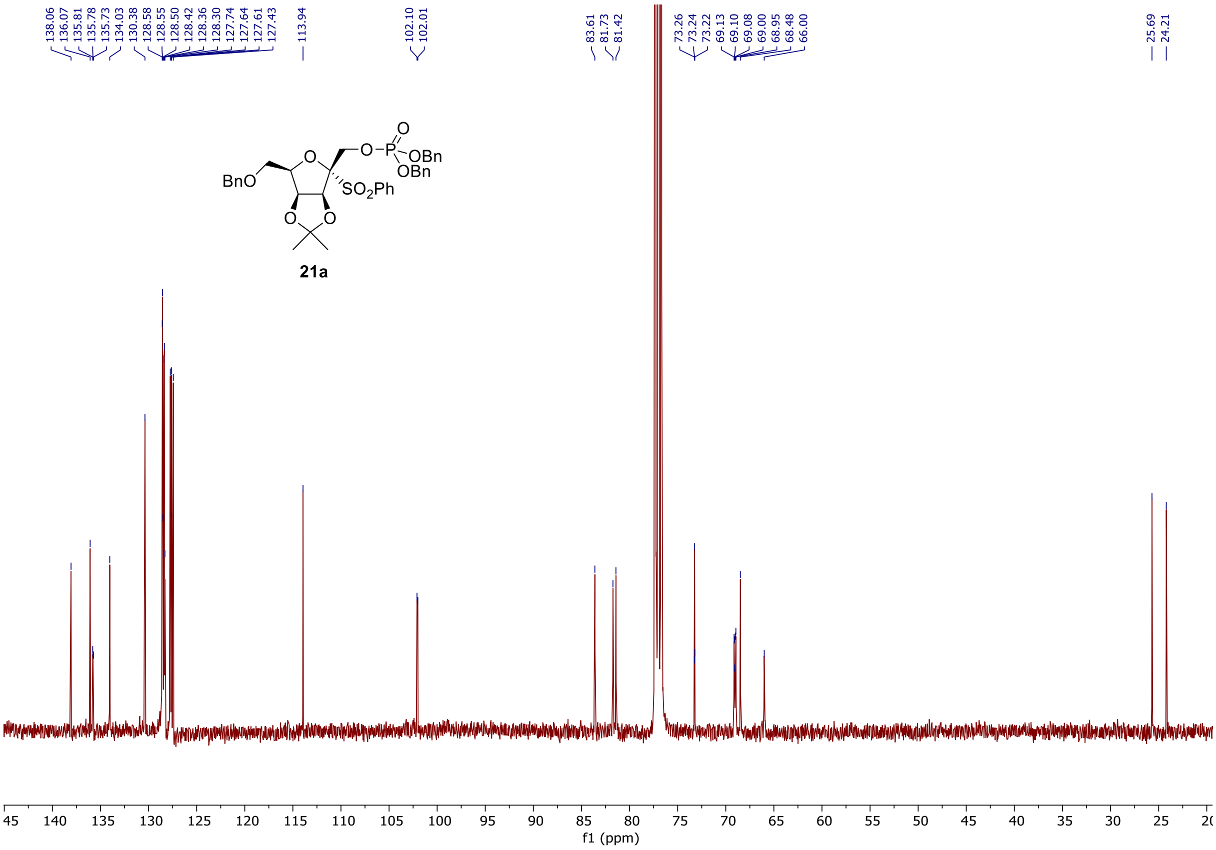

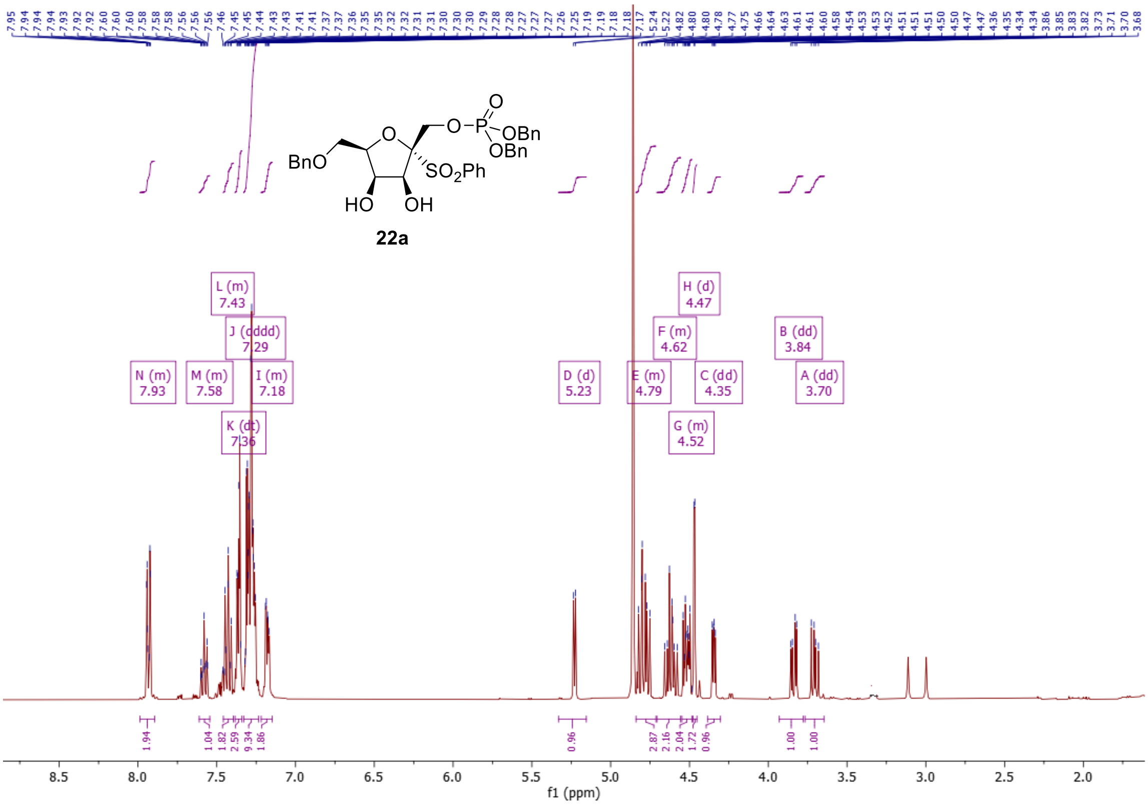

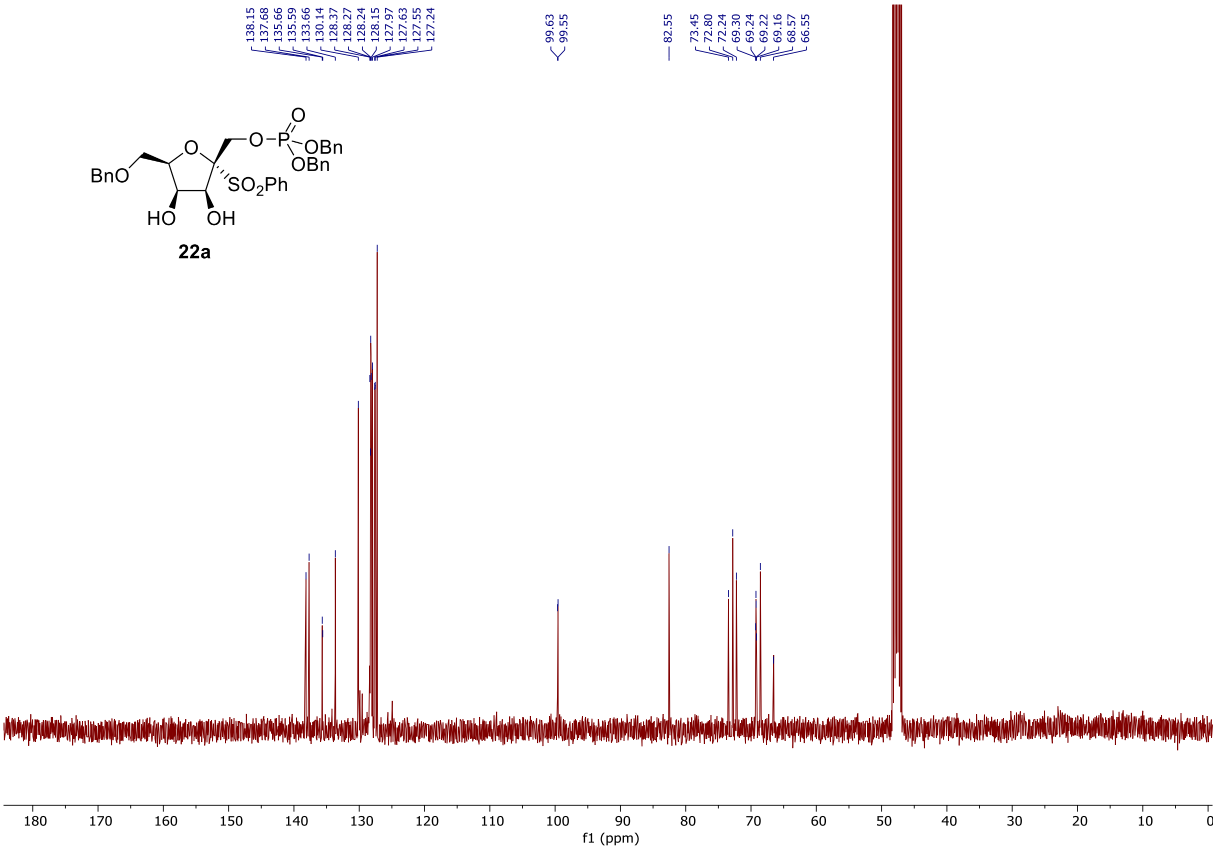

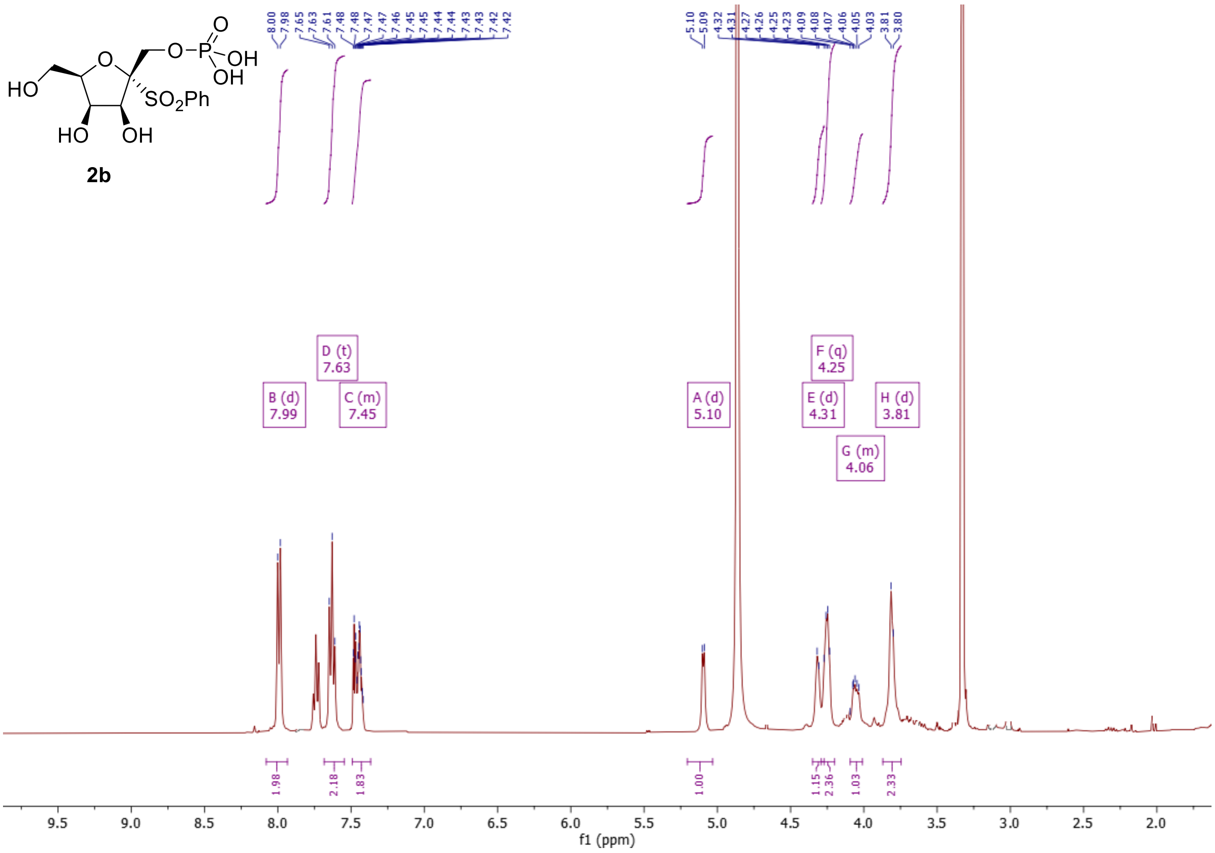
HO
O
O–P
O
OH
OH
SO2Ph
HO
OH
2b
B (d)
7.99
D (t)
7.63
C (m)
7.45
A (d)
5.10
E (d)
4.31
F (q)
4.25
G (m)
4.06
H (d)
3.81
1.98
2.18
1.83
1.00
1.15
2.36
1.03
2.33
f1 (ppm)

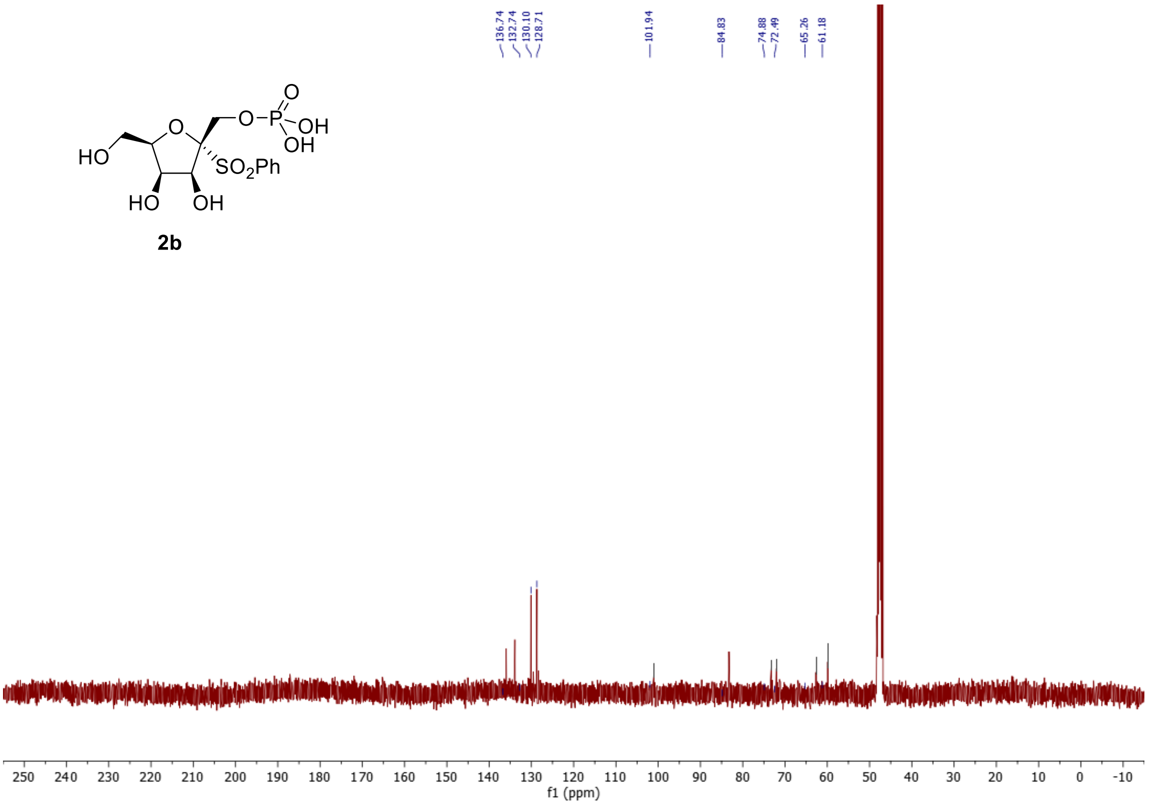
136.74
132.74
130.10
128.71
101.94
84.83
74.88
72.49
65.26
61.18
HO
O
O–P
O
OH
OH
SO2Ph
HO
OH
2b
f1 (ppm)

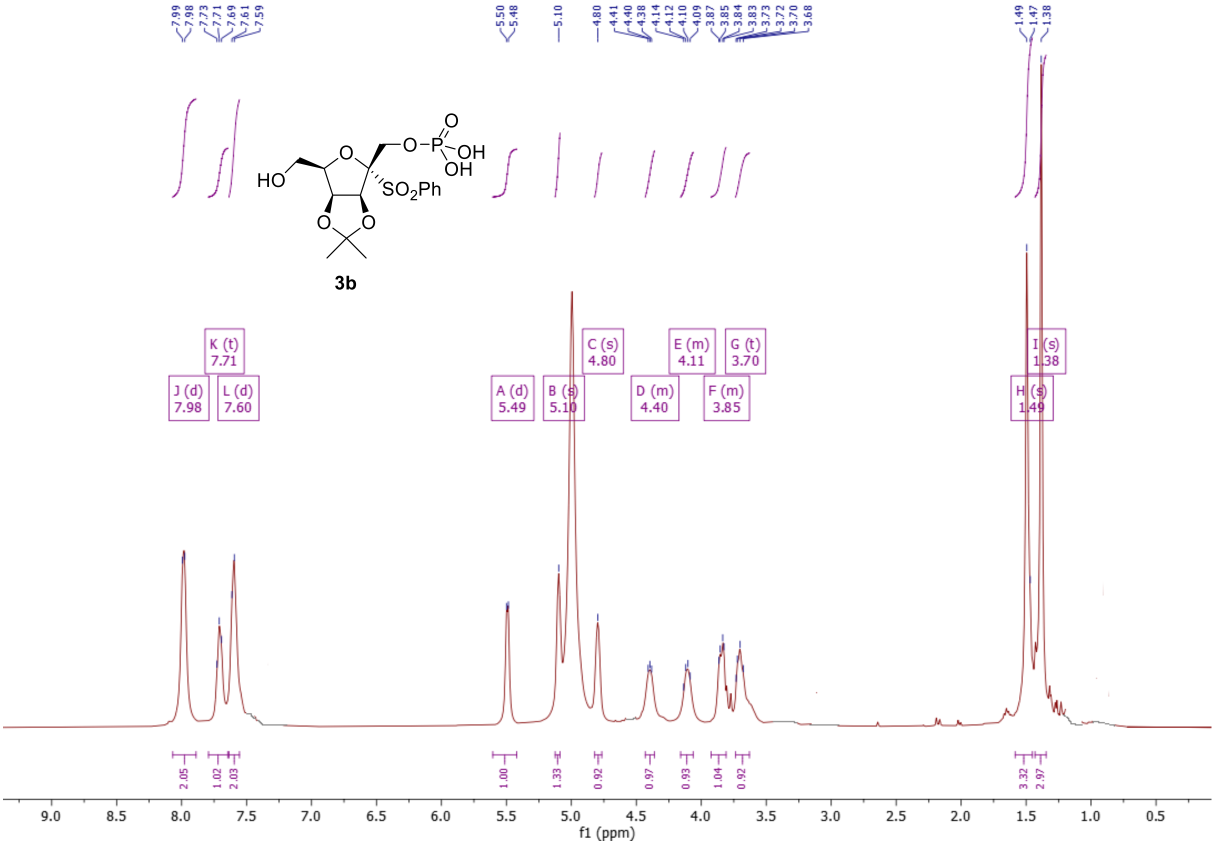
7.99 7.98 7.73 7.71 7.69 7.61 7.59
5.50 5.48
5.10
4.80 4.41 4.40 4.38 4.14 4.12 4.10 4.09 3.87 3.85 3.84 3.83 3.73 3.72 3.70 3.68
1.49 1.47 1.38
HO
O
O
P
OH
OH
SO2Ph
3b
J (d) 7.98
K (t) 7.71
L (d) 7.60
A (d) 5.49
B (s) 5.10
C (s) 4.80
D (m) 4.40
E (m) 4.11
F (m) 3.85
G (t) 3.70
H (s) 1.49
I (s) 1.38
2.05 1.02 2.03 1.00 1.33 0.92 0.97 0.93 1.04 0.92 3.32 2.97
f1 (ppm)

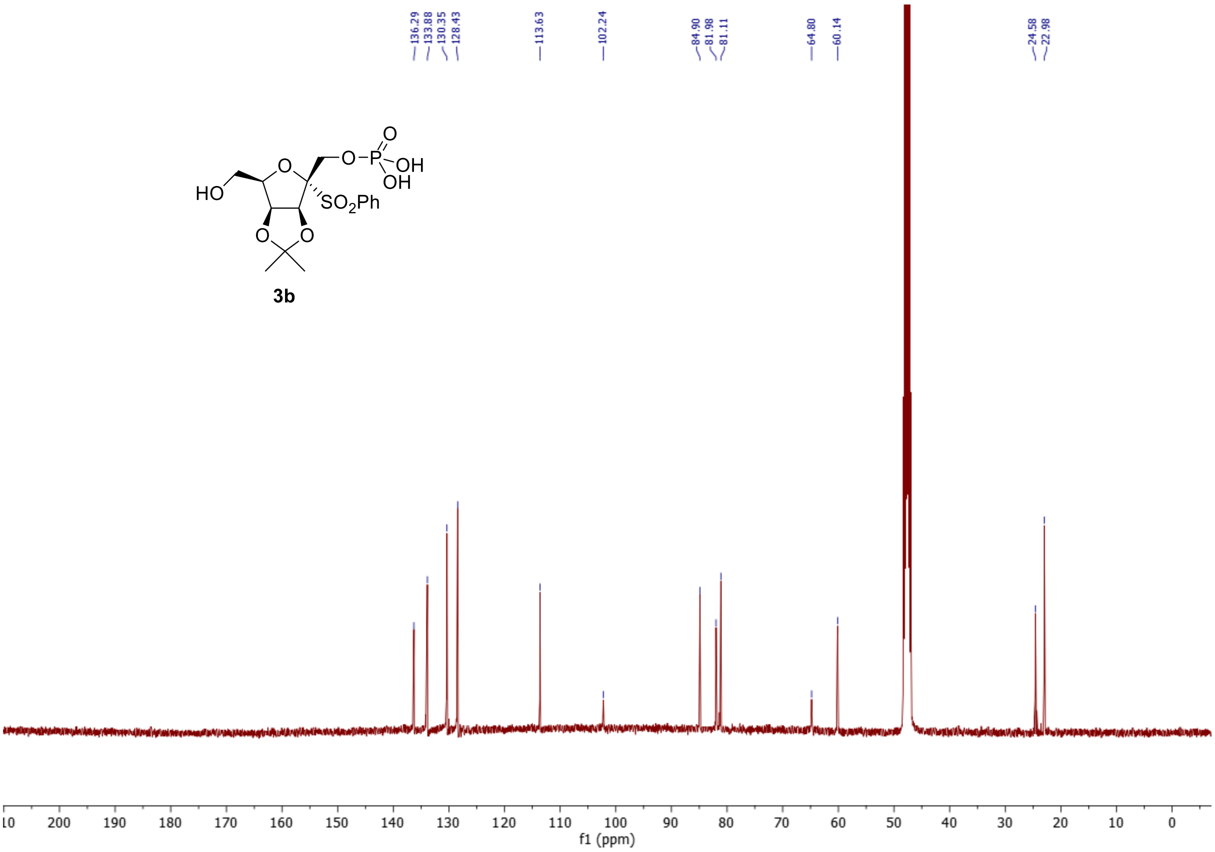
136.29 133.88 130.35 128.43
113.63
102.24
84.90 81.98 81.11
64.80
60.14
24.58 22.98
HO
O
O
P
OH
OH
SO2Ph
3b
f1 (ppm)

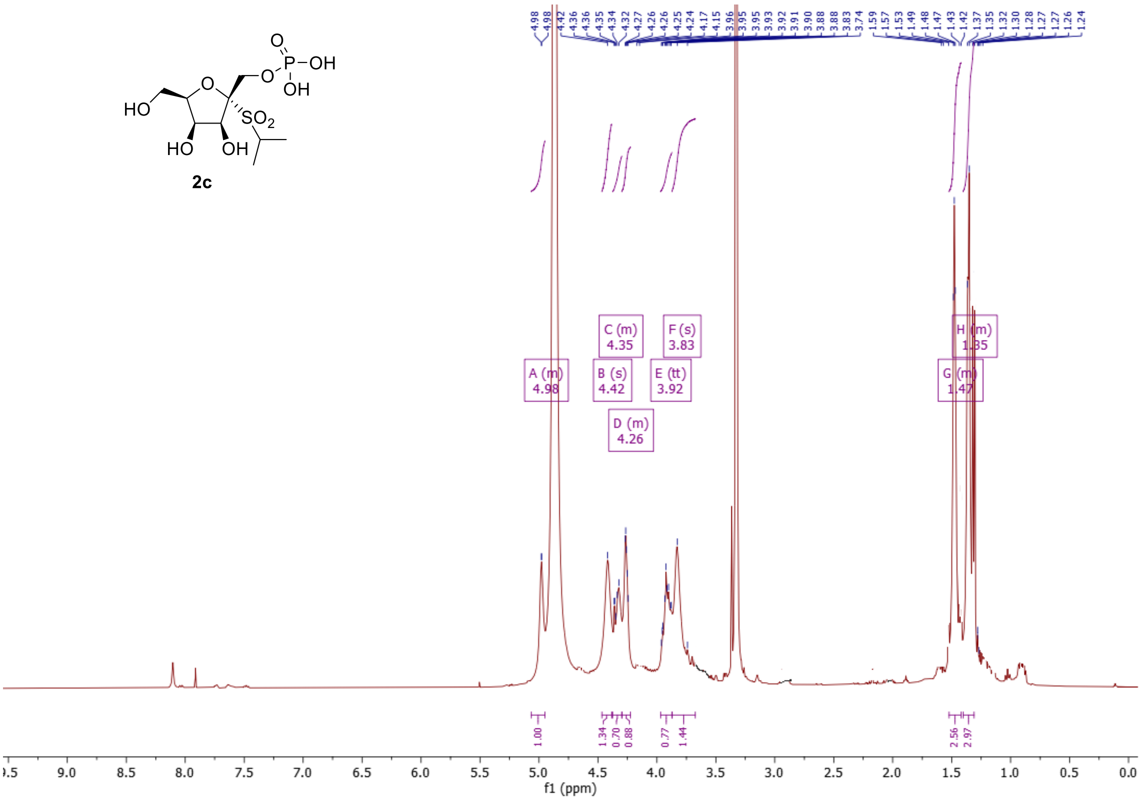
2c
A (m) 4.98
B (s) 4.42
C (m) 4.35
D (m) 4.26
E (tt) 3.92
F (s) 3.83
G (m) 1.47
H (m) 1.35
f1 (ppm)

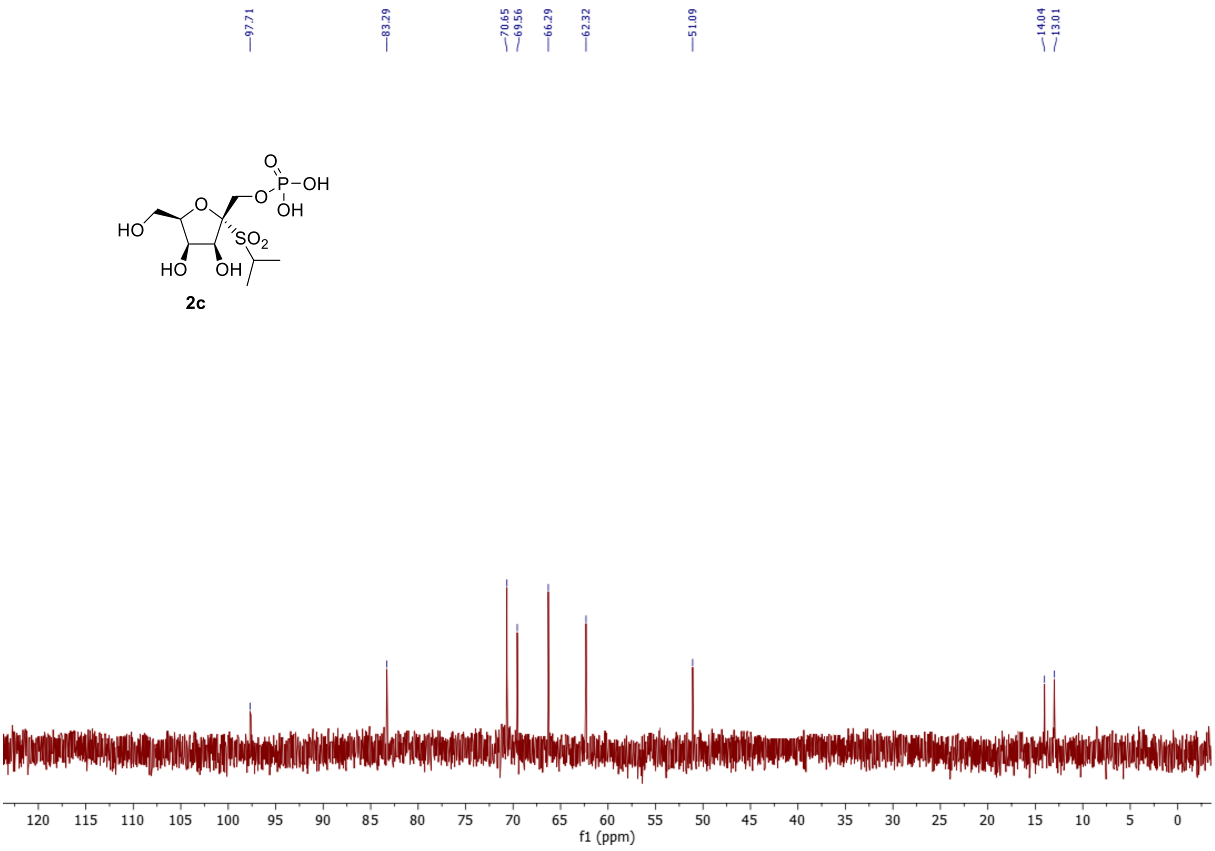
97.71
83.29
70.65
69.56
66.29
62.32
51.09
14.04
13.01
2c
f1 (ppm)

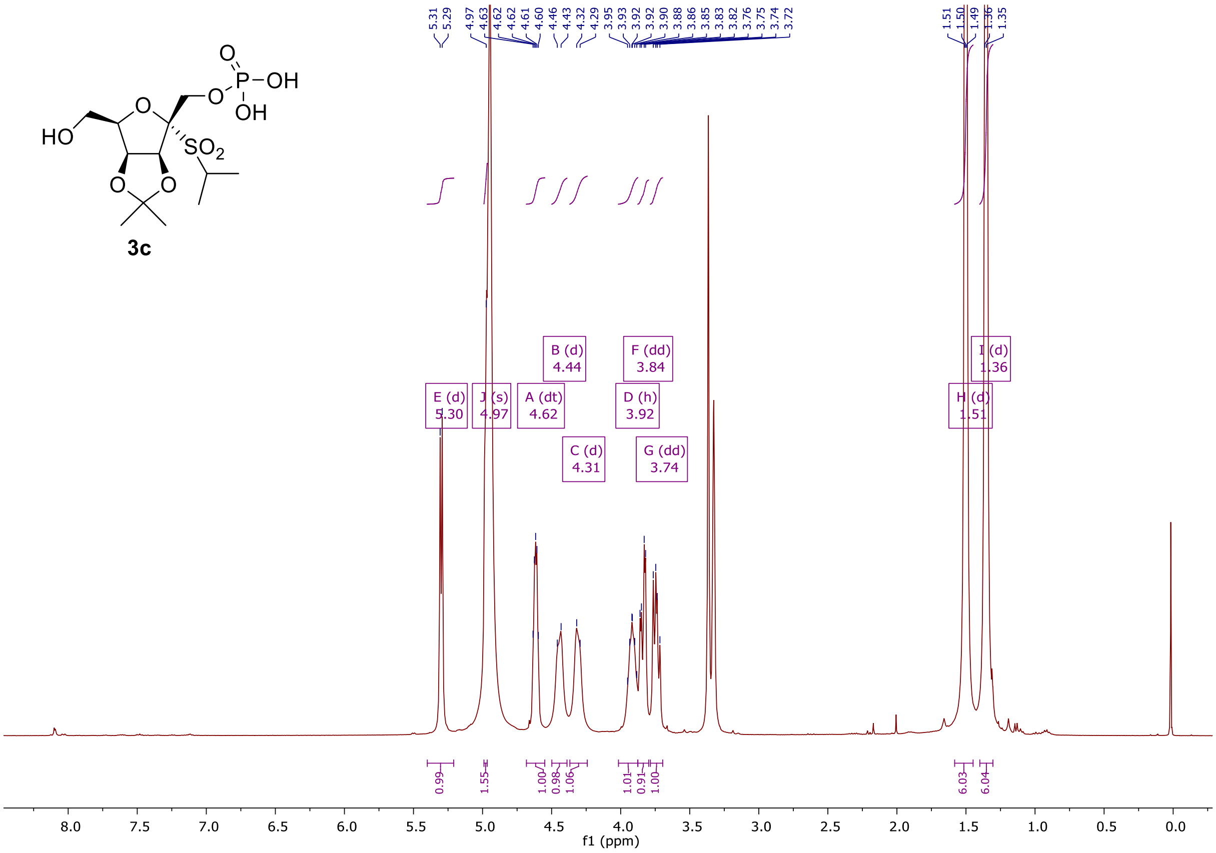
3c
5.31
5.29
4.97
4.63
4.62
4.62
4.61
4.60
4.46
4.43
4.32
4.29
3.95
3.93
3.92
3.92
3.90
3.88
3.86
3.85
3.83
3.82
3.76
3.75
3.74
3.72
1.51
1.50
1.49
1.36
1.35
E (d) 5.30
J (s) 4.97
A (dt) 4.62
B (d) 4.44
C (d) 4.31
D (h) 3.92
F (dd) 3.84
G (dd) 3.74
H (d) 1.51
I (d) 1.36
0.99
1.55
1.00
0.98
1.06
1.01
0.91
1.00
6.03
6.04
f1 (ppm)

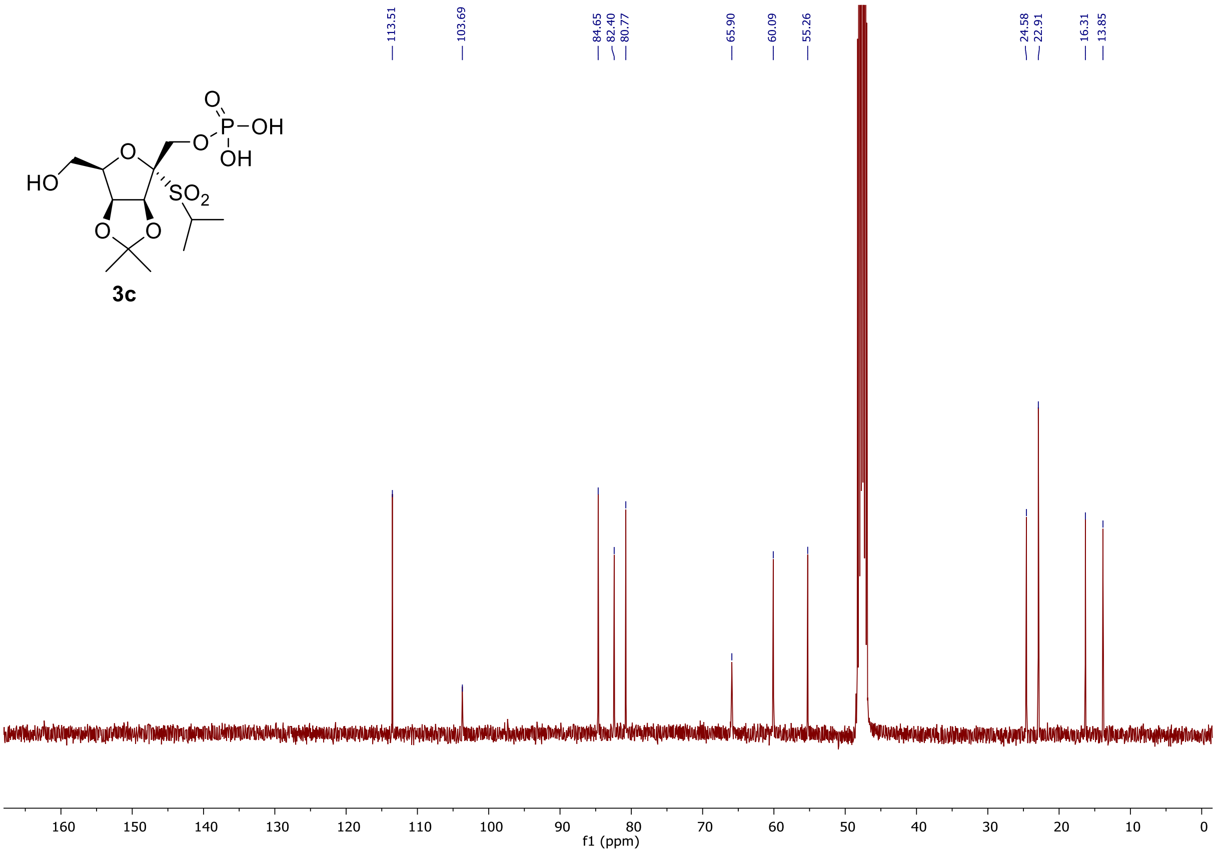
3c
113.51
103.69
84.65
82.40
80.77
65.90
60.09
55.26
24.58
22.91
16.31
13.85
f1 (ppm)

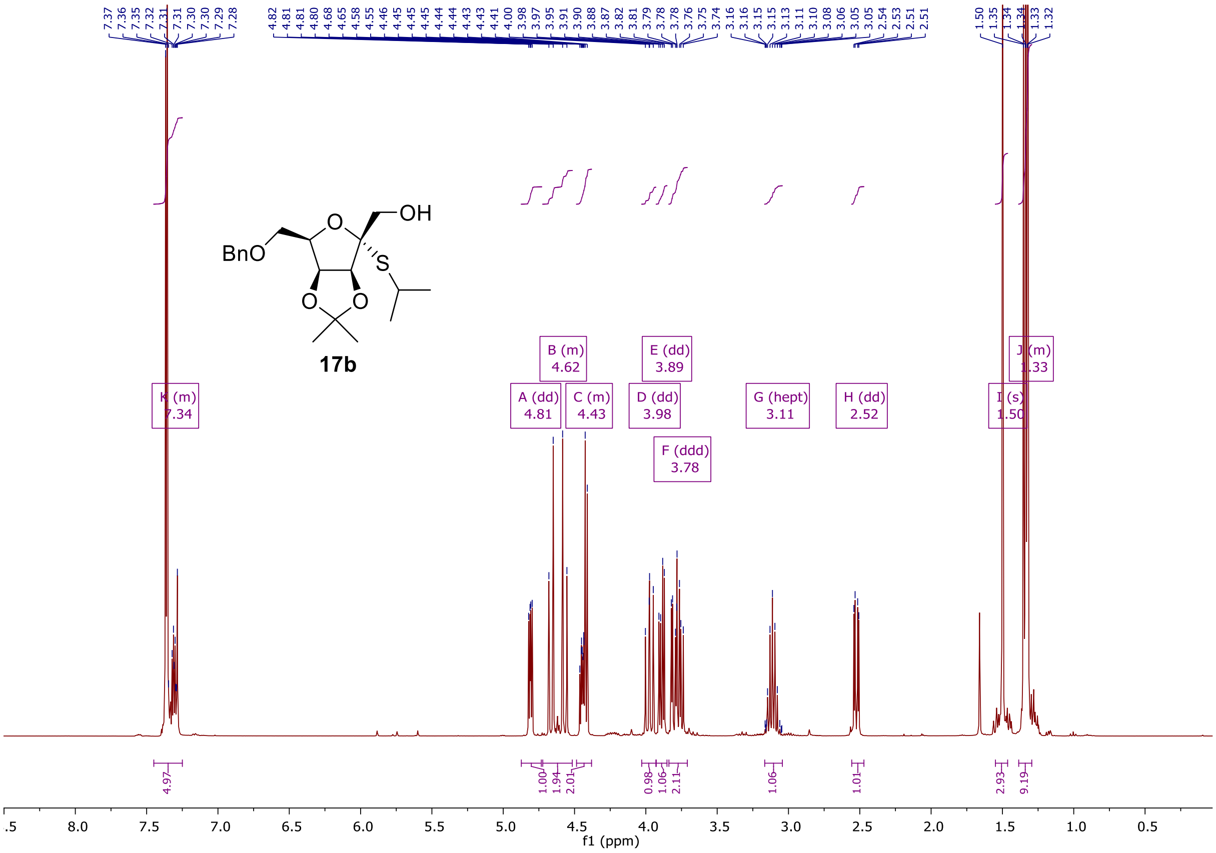
7.37 7.36 7.35 7.32 7.31 7.31 7.30 7.30 7.29 7.28
4.82 4.81 4.81 4.80 4.68 4.65 4.58 4.55 4.46 4.45 4.45 4.45 4.44 4.44 4.43 4.41 4.00 3.98 3.97 3.95 3.91 3.90 3.88 3.87 3.82 3.81 3.79 3.78 3.78 3.76 3.75 3.74 3.16 3.15 3.15 3.13 3.11 3.10 3.08 3.06 3.05 3.05 2.54 2.53 2.51 2.51
1.50 1.35 1.34 1.34 1.33 1.32
BnO
OH
O
S
O
O
17b
K (m) 7.34
A (dd) 4.81
B (m) 4.62
C (m) 4.43
D (dd) 3.98
E (dd) 3.89
F (ddd) 3.78
G (hept) 3.11
H (dd) 2.52
I (s) 1.50
J (m) 1.33
4.97
1.00
1.94
2.01
0.98
1.06
2.11
1.06
1.01
2.93
9.19
f1 (ppm)

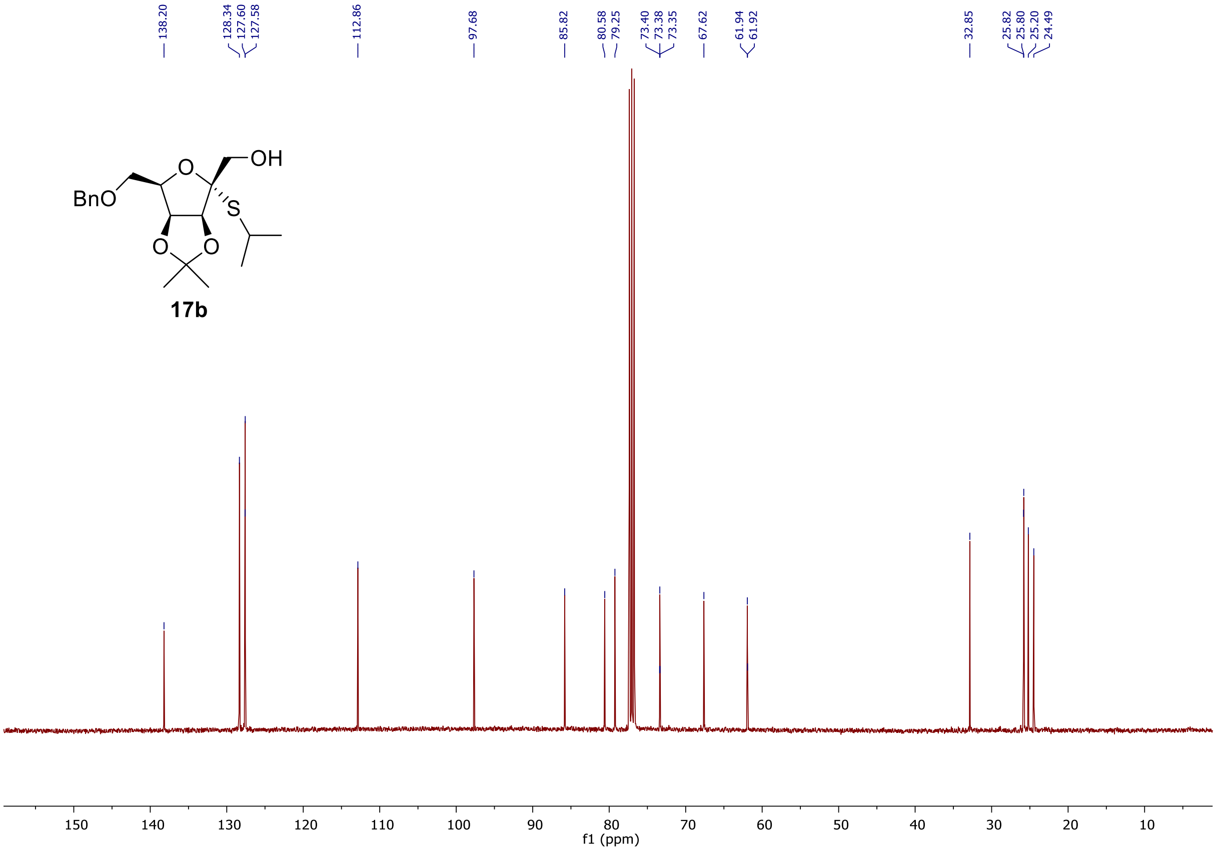
138.20
128.34 127.60 127.58
112.86
97.68
85.82
80.58 79.25
73.40 73.38 73.35
67.62
61.94 61.92
32.85
25.82 25.80 25.20 24.49
BnO
OH
O
S
O
O
17b
f1 (ppm)

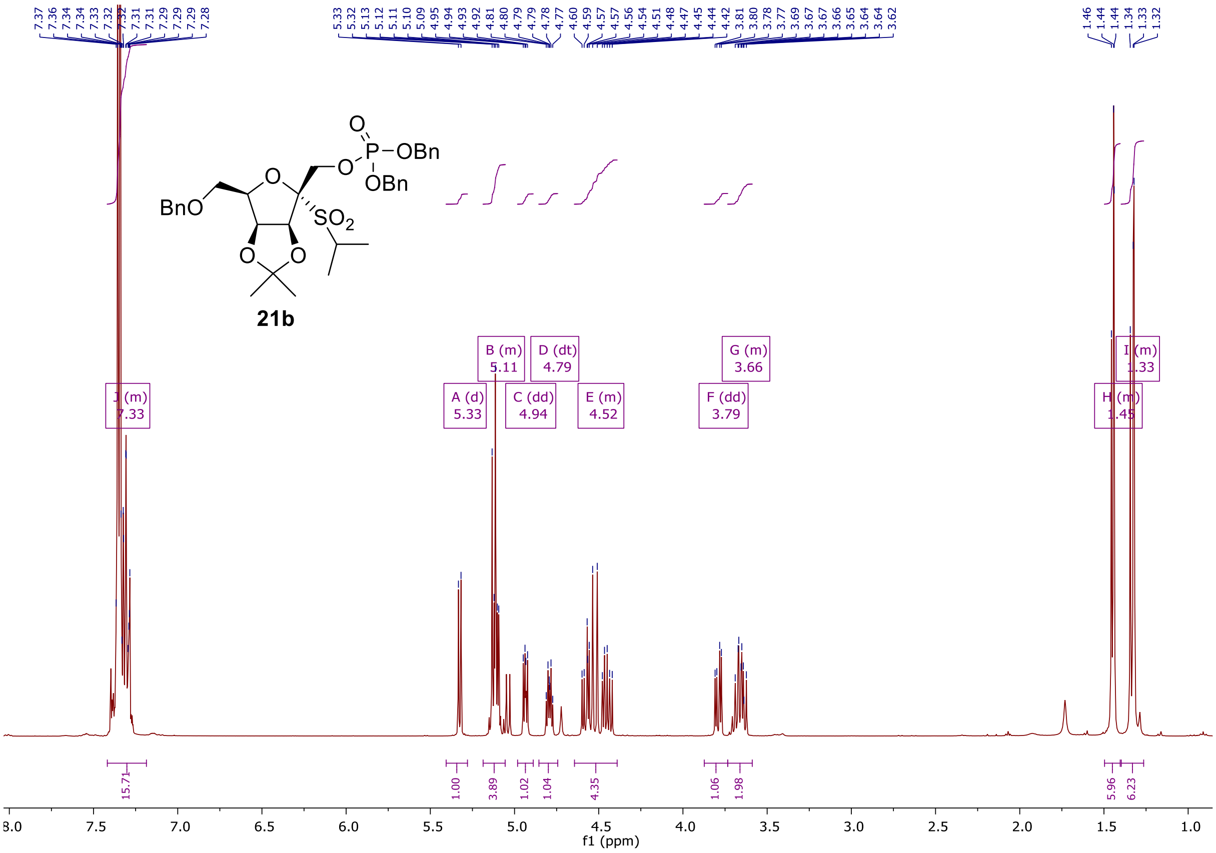
21b
f1 (ppm)

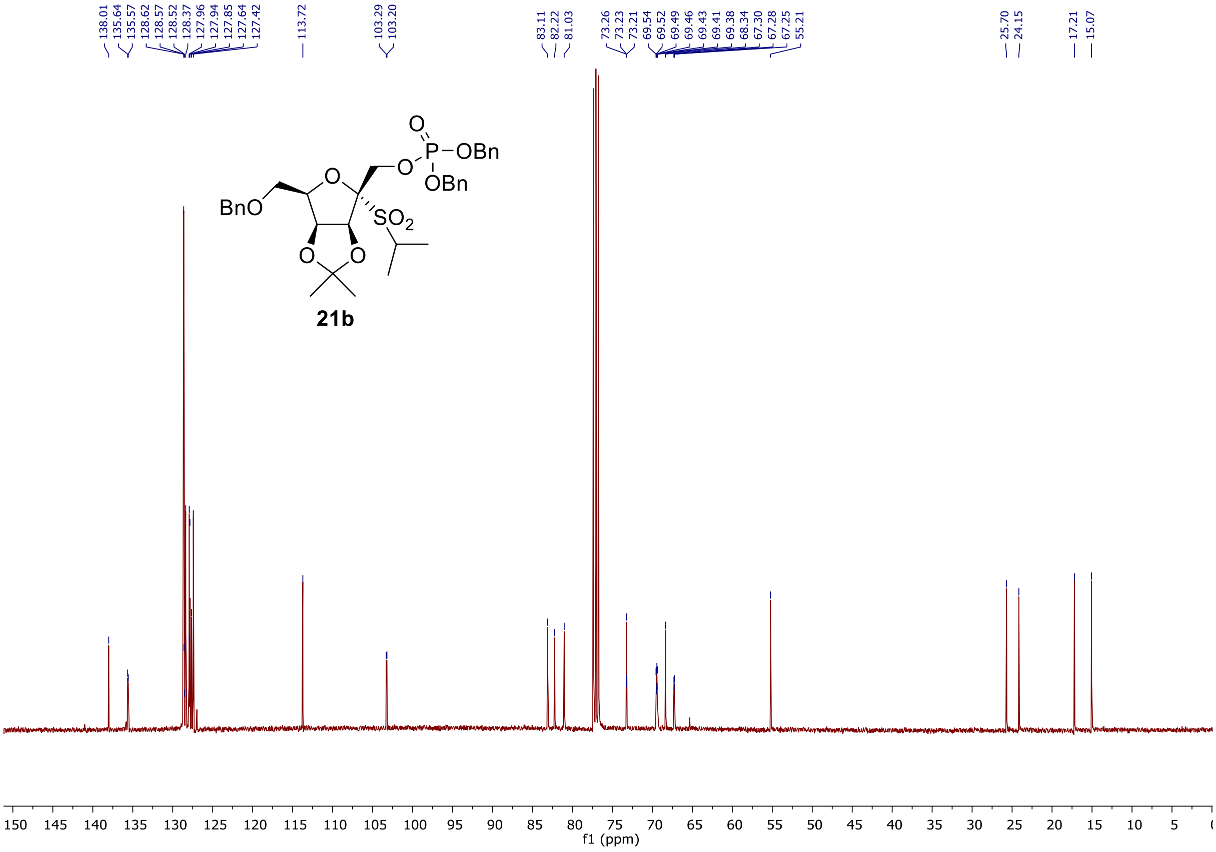
21b
f1 (ppm)

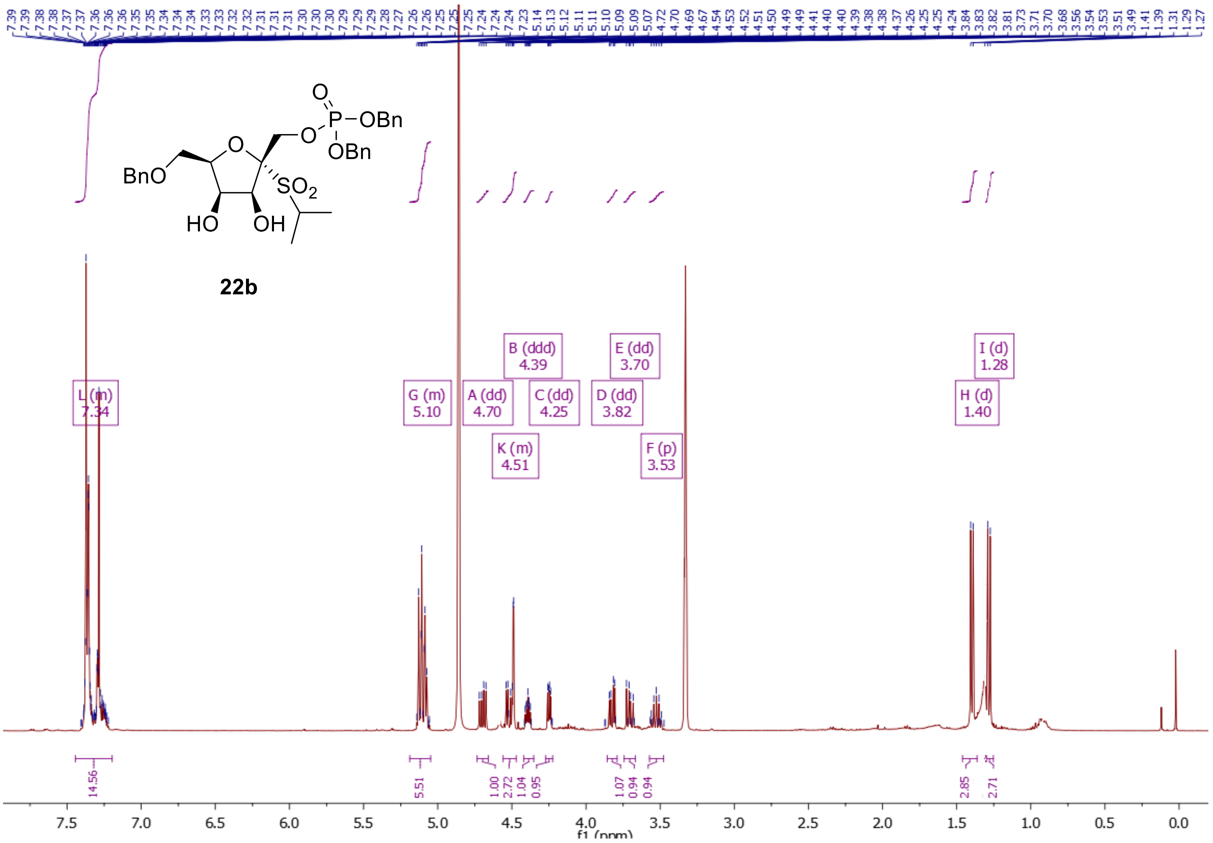

BnO
HO
OH
O
P
OBn
SO2
22b
L (m)
7.34
G (m)
5.10
A (dd)
4.70
B (ddd)
4.39
K (m)
4.51
C (dd)
4.25
D (dd)
3.82
E (dd)
3.70
F (p)
3.53
H (d)
1.40
I (d)
1.28
14.56
5.51
1.00
2.72
1.04
0.95
1.07
0.94
0.94
2.85
2.71
f1 (ppm)

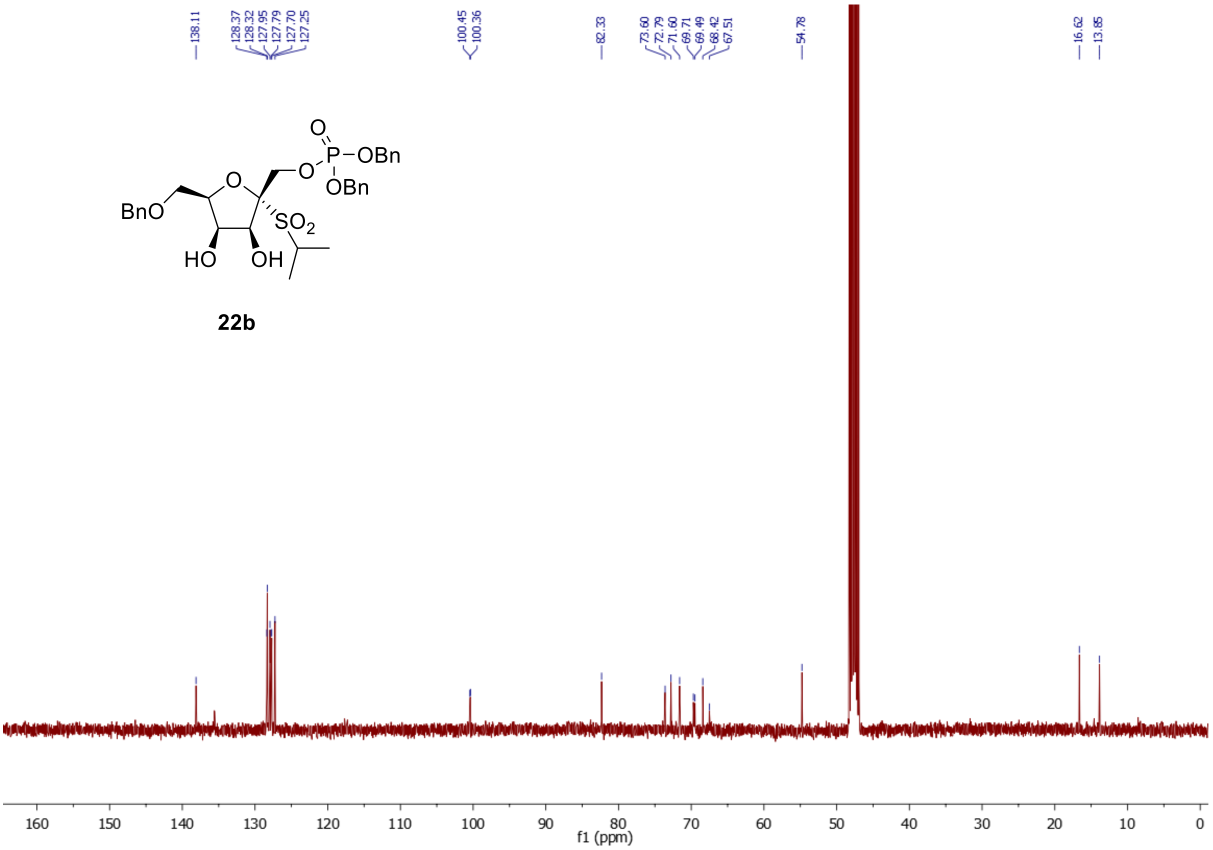

BnO
HO
OH
O
P
OBn
SO2
22b
138.11
128.37
128.32
127.95
127.79
127.70
127.25
100.45
100.36
82.33
73.60
72.79
71.60
69.71
69.49
68.42
67.51
54.78
16.62
13.85
f1 (ppm)
